# Transient bis(carboranyl)boryl anions

**DOI:** 10.1039/d6sc03158g

**Published:** 2026-06-25

**Authors:** Kanika Vashisth, Ragene A. Thornton, Bohak Yoon, Caleb D. Martin

**Affiliations:** a Baylor University, Department of Chemistry and Biochemistry One Bear Place #97348 Waco TX 76798 USA caleb_d_martin@baylor.edu

## Abstract

The reduction of bromoboranes with two *ortho*-carborane substituents results in the generation of transient boryl anions. DFT computations reveal that their ground state is significantly different from previously reported examples as an unusual linear singlet engendered by negative hyperconjugation stabilization by the carboranes. The substitution on the *ortho*-carbon of the carborane is influential on the fate of the anion with intramolecular C–H insertion and cyclization to generate five-membered boracycles occurring with phenyl or trimethylsilyl groups in this position. Gratifyingly, methyl substitution enables intermolecular *in situ* boryl anion reactivity as a nucleophile to generate a copper–boryl complex and in a cycloaddition with tetrachloro-*o*-benzoquinone.

## Abstract 2

The reduction of bromoboranes with two *ortho*-carborane substituents results in the generation of transient boryl anions that underwent intramolecular C–H insertion or *in situ* boryl anion reactivity.

## Graphical abstract

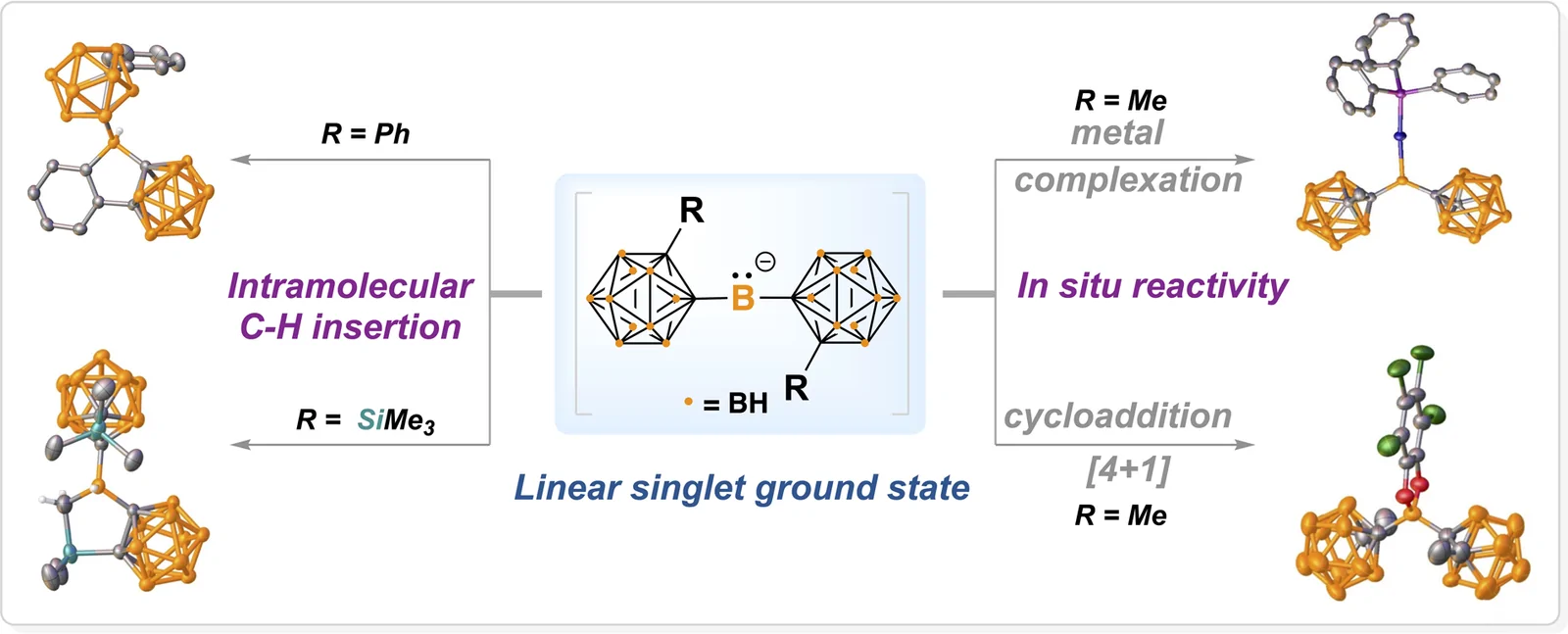

## Introduction

**Fig. 1:**
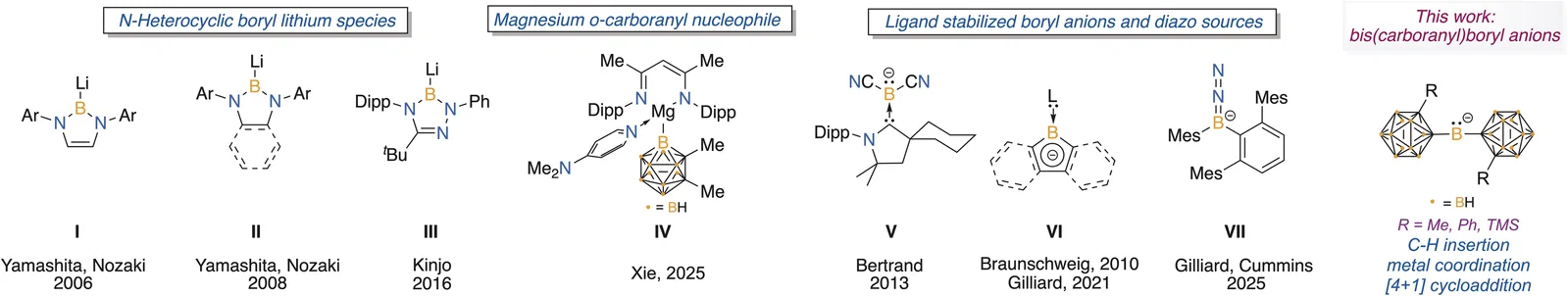
Structure of selected ligand stabilized boryl anions and this work (Dipp = 2,6-diisopropylphenyl), Mes = 2,4,6-trimethylphenyl).

Carbenes are neutral dicoordinate species with a sextet that are critical reactive intermediates in synthesis.^1–5^ Their isoelectronic boron analogues, boryl anions, have been much less studied.^6–8^ Late metal complexes of both carbenes and boryl anions are prevalent as the sextet can be stabilized by metal to ligand π-back-bonding interactions, however species lacking this stabilization are much more scarce.^9–15^ In 2006, Yamashita and Nozaki reported the reduction of an unsaturated *N*-heterocyclic-bromoborane to generate a boryl–lithium complex (NHB, **I**, Fig. 1) that they were able to crystallographically characterize.^16^ This groundbreaking discovery enabled nucleophilic reactivity studies of **I**, an Umpolung for boron chemistry.^17,18^ The backbone of the boryl lithium framework can be modified with a saturated ethylene backbone or a benzo-fused variant (**II**) and a triazole species (N_3_CB, **III**).^17,19,20^ The NHB species are superior sigma donors to NHCs making them very attractive ligands.^21^ Since the pioneering boryl-lithium work, nucleophilic potassium, magnesium, copper and zinc boryl variants have been prepared.^19,22–28^ In 2025, Xie isolated a boron–magnesium complex where the boron is part of an *ortho*-carborane cluster (**IV**) capable of nucleophilic reactivity.^29^

While isolable species are restricted to boryl metal systems of types **I–IV**, ligand stabilization has been very effective for sequestering boryl anions. Bertrand revealed that a carbene is a potent donor to enable isolation of the Lewis base stabilized dicyanoboryl anion (**V**).^30^ In cyclic species, Braunschweig reported that a carbene could stabilize the borolide anion and Gilliard demonstrated that it can be leveraged for the stabilization of species with extended conjugation in polycyclic aromatic hydrocarbons (**VI**).^23,31^ The latter discovery included a carbene ligated borafluorenyl anion that can react as the free boryl anion due to the labile carbene.^31–33^ Kinjo reported the reduction of 9-bromo-9-borafluorene to a trimerized cyclotriborate anion that proceeds *via* the same borafluorenyl anion.^34–36^ Beyond cyano species,^37^ the only other acyclic boryl anion was a diaryl boryl anion reported last year by Gilliard and Cummins generated *in situ via* N_2_ liberation from an anionic diazoborane with a mesityl group and very bulky terphenyl group on boron (**VII**).^38,39^ Inspired by these efforts in using bulky aryl groups and the effectiveness of electron withdrawing cyano groups for boryl anions, we surmised that the tremendous bulk and electron withdrawing ability of *ortho*-carboranes^40–51^ could function as effective substituents for boryl anions. We herein report our efforts to generate a bis(*ortho*-carboranyl)boryl anion.

## Results and discussion

**Scheme 1:**
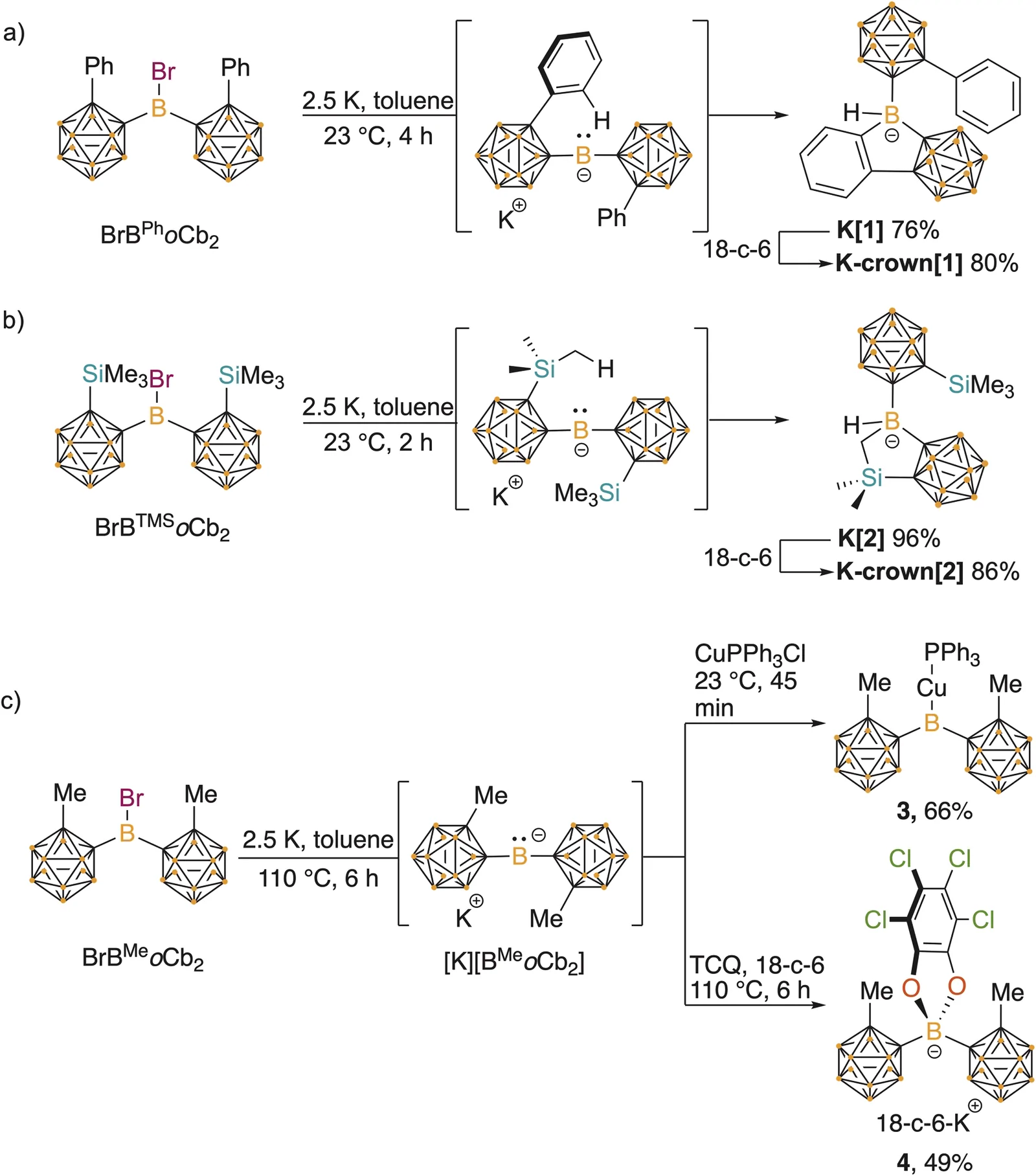
Reduction of BrB^Ph^*o*Cb_2_ (a) and BrB^TMS^*o*Cb_2_ (b) with potassium to generate C–H borylated species. (c) *In situ* generation of [K][B^Me^*o*Cb_2_] and reactivity with CuPPh_3_Cl and tetrachloro-*o*-benzoquinone (TCQ).

**Fig. 2:**
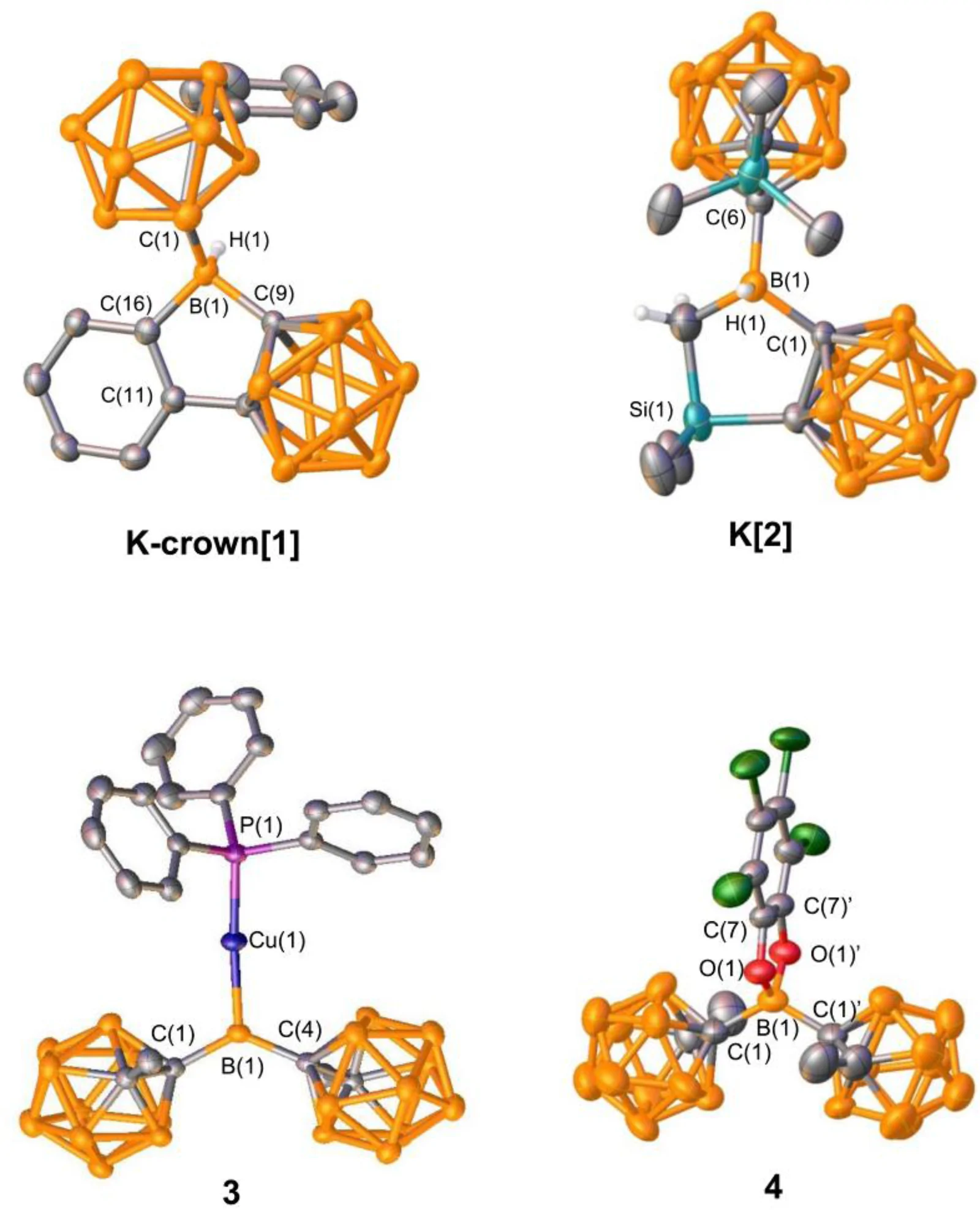
Solid state structures of K-crown[1], K[2], 3 and 4. Ellipsoids depicted at the 50% probability level. Cations and hydrogen atoms (except on the central boron atom) are omitted for clarity. Selected bond lengths (Å) and angles (°): K-crown[1]: B(1)–C(1) 1.633(4), B(1)–C(9) 1.672(3), B(1)–C(16) 1.629(4), C(11)–C(16) 1.395(4), C(1)–B(1)–C(9) 117.5(2), C(16)–B(1)–C(9) 100.91(19); K[2]: B(1)–C(1) 1.659(5), B(1)–C(6) 1.670(4), C(6)–B(1)–C(1) 116.8(2). 3: B(1)–C(1) 1.633(3), B(1)–C(4) 1.624(3), B(1)–Cu(1) 2.153(2), P(1)–Cu(1) 2.180(5), B(1)–Cu(1)–P(1) 164.06(6), C(1)–B(1)–C(4) 125.30(15); 4: B(1)–C(1) 1.656(6), B(1)–O(1) 1.514(5), C(1)–B(1)–C(1)′ 120.6(3).

The reduction of BrB^Ph^*o*Cb_2_ with 2.5 equivalents of potassium in toluene at 23 °C was monitored by ^11^B NMR spectroscopy (Scheme 1a).^52^ After 4 h, the peak at 64.1 ppm for BrB^Ph^*o*Cb_2_ is consumed and a white precipitate generated. Adding 18-crown-6 solubilized the material to enable acquisition of multinuclear NMR spectra and growth of single crystals. The identity was determined by single crystal X-ray diffraction as a five membered BC_4_ heterocycle composed of the central boron, two carbons of a phenyl group, and two carbons from the *ortho*-carborane with a pendent ^Ph^*o*Cb substituent on boron (K-crown[1], Fig. 2). The borylation product is consistent with boryl anion generation and insertion into the aryl *ortho* C–H bond. In the ^13^C{^1^H} NMR spectrum, two peaks for the *ortho*-carbons of the carborane at 88.1 and 86.8 ppm indicate chemical inequivalence of the carborane groups.

Since C_sp^3^_–H bonds are less reactive than C_sp^2^_–H bonds, we attempted an analogue of BrB^Ph^*o*Cb_2_ bearing trimethylsilyl (TMS) groups in place of the phenyl substituents. The requisite bromoborane, BrB^TMS^*o*Cb_2_, was accessed by reaction of 1-trimethylsilyl-*ortho*-carborane (^TMS^*o*Cb) with an equivalent of *n*BuLi and addition of half an equivalent of BBr_3_ at −78 °C. The identity of BrB^TMS^*o*Cb_2_ was confirmed by single crystal X-ray diffraction and the ^11^B{^1^H} NMR spectrum featured a downfield resonance for the central boron at 64.1 ppm. The reduction of BrB^TMS^*o*Cb_2_ with potassium in toluene at 23 °C was monitored by ^11^B{^1^H} NMR spectroscopy (Scheme 1b). After 2 h, the resonance for BrB^TMS^*o*Cb_2_ at 64.1 ppm was consumed. In the ^1^H NMR spectrum, three singlets in the silyl region were observed at 0.50, 0.37, and 0.31 ppm integrating in a 9 : 3 : 3 ratio and two doublets, each integrating to one at 0.56 and 0.54 ppm, with matching coupling constants of 6 Hz. The ^29^Si{^1^H} NMR spectrum revealed resonances at 20.4 and 6.9 ppm, contrasting the spectrum of BrB^TMS^*o*Cb_2_ that has a single peak at 11.6 ppm, indicating a break in symmetry. The identity was determined by single crystal X-ray diffraction as the five-membered boracycle K[2] resulting from C_sp^3^_–H insertion of the boron atom into a methyl group of the TMS substituent. Sequestering the potassium ion by 18-crown-6 at room temperature enabled isolation of the crowned species, K-crown[2].

The two intramolecular C–H borylation products imply generation of a transient boryl anion intermediate that reacts with the *ortho*-substituent. To circumvent five-membered boracycle formation, we chose a methyl substituted variant, BrB^Me^*o*Cb_2_,^53^ as the putative C–H insertion to a four-membered boracycle should be less favorable due to ring strain. Reduction attempts of BrB^Me^*o*Cb_2_ with potassium at 23 °C for 24 h in toluene did not result in any reaction. However, heating at 110 °C for 6 h resulted in the disappearance of the tricoordinate peak for BrB^Me^*o*Cb_2_ at 65.0 ppm in the ^11^B NMR spectrum. Over time, decomposition occurred to multiple species with [K][H_2_B^Me^*o*Cb_2_] being a species identified in the mixture by single crystal X-ray diffraction and by the multinuclear NMR spectroscopic signatures of the anion matching those reported of H_2_B^Me^*o*Cb_2_^–^ paired with phosphonium cations.^54^ Despite significant efforts, we were unable to isolate or characterize a boryl anion species. To determine if a boryl anion was generated, the solution was treated *in situ* with an equivalent of Ph_3_PCuCl at 23 °C (Scheme 1c). The identity was confirmed as the copper–boryl complex 3 by single crystal X-ray diffraction (Fig. 2). Upon work up, 3 was isolated in 66% yield. The resonance for the central boron in the ^11^B NMR spectrum is at 23.5 ppm and the ^31^P{^1^H} NMR spectrum features a singlet at −4.2 ppm. The B–Cu distance is 2.153(2) Å, longer than in reported NHCCuBpin or NHCCuBcat complexes that range 1.97 to 2.03 Å, attributed to the tremendous bulk of the carboranyl substituents.^24,55,56^

Boryl anions are isostructural and isoelectronic to carbenes, that readily undergo cycloaddition reactions. Reaction of *in situ* generated [K][B^Me^*o*Cb_2_] with an equivalent of 18-crown-6 and tetrachloro-*o*-benzoquinone (TCQ) in toluene resulted in a new peak at 14.4 ppm in the ^11^B NMR spectrum after 6 h of heating at 110 °C. The product was identified by single crystal X-ray diffraction as the boryl anion [4 + 1] cycloaddition product 4 with the central boron in a pseudo-tetrahedral geometry with two new B–O bonds. The C–C bond of the quinone carbons is considerably reaction shortened compared to that in TCQ [1.379(11) Å *c.f.* 1.553(5) Å] and the C–O bonds lengthened [1.344(5) Å *c.f.* 1.201(4) Å],^57^ consistent with a pericyclic reaction with the boryl anion. It is notable that NHB species have not been reported to undergo cycloaddition reactions, suggesting B^Me^*o*Cb_2_^−^ has a considerably different electronic structure.

**Fig. 3:**
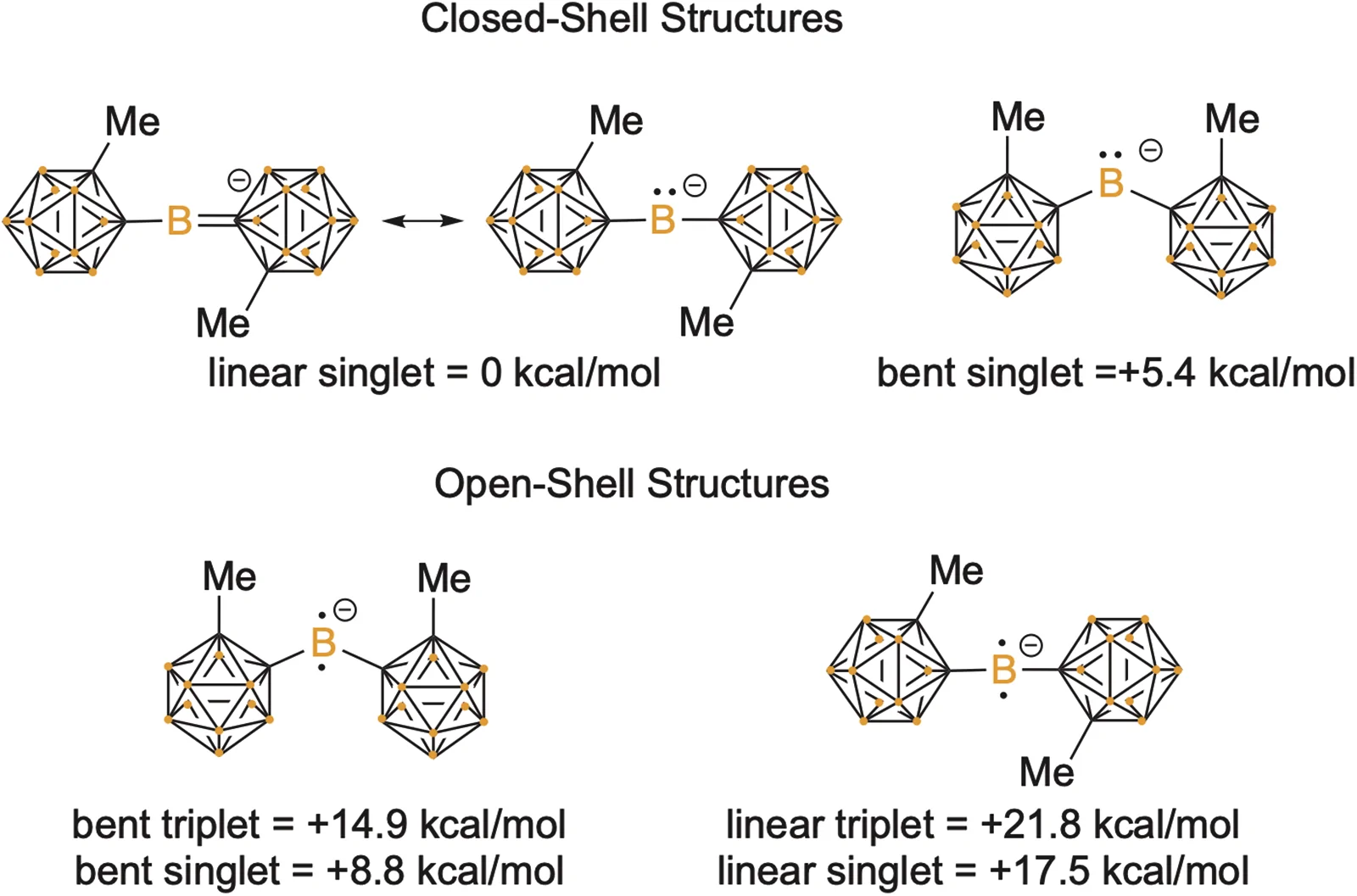
Computed structures for B^Me^*o*Cb_2_^−^ with energies relative to the linear singlet. In the open shell configurations, the triplet is where the two electrons have the same spin while the singlet is that with opposite spins.

To provide insight into the electronic structures of the bis(*ortho*-carboranyl)boryl anions, B^Me^*o*Cb_2_^−^, B^Ph^*o*Cb_2_^−^, and B^TMS^*o*Cb_2_^−^, comparative density functional theory (DFT) calculations at the ωB97X-D/def2-TZVPP level using the implicit solvation model with toluene as the dielectric medium based on density (SMD) were conducted.^58–61^ The values were computed for bent and linear geometries in the singlet and triplet states for B^Me^*o*Cb_2_^−^. Interestingly, the lowest in energy is the linear singlet where multiple bonding exists between boron and one of the carbon atoms of the carborane but with considerable electron density on boron, reflected in the two canonical representations (Fig. 3). The next lowest energetic state is the bent singlet (+5.4 kcal mol^−1^) while all of the open shell configurations are computed to be higher in energy. For the open shell structures, the bent and linear geometries are possible where the electrons have the opposite spin (singlet) or same spin (triplet). The bent triplet is at +14.9 kcal mol^−1^ and the linear triplet at +21.8 kcal mol^−1^. The open shell triplet states are slightly lower in energy with the bent structure at +8.8 kcal mol^−1^ and the linear geometry at +17.5 kcal mol^−1^. The free energy differences between the bent and linear singlet states for B^Ph^*o*Cb_2_^−^, and B^TMS^*o*Cb_2_^−^ are less pronounced at 2.8 and 1.9 kcal mol^−1^, respectively, but confirm that the linear-singlet is the ground state in all three variants.

Examining the bonding of the linear singlet of B^Me^*o*Cb_2_^−^ by natural bond orbital (NBO) analysis reveal the central boron atom is best described as sp-hybridized with one π and two σ bonds. The π bond between boron and one of the ipso carbons features a negative hyperconjugative interaction with the carborane and the bond polarized toward boron. This is consistent with the computed partial atomic charge of −0.42 e on the boron atom based on population analyses. The computed C–B bond lengths are non-symmetric at 1.37 and 1.51 Å, with corresponding Wiberg bond indices (WBIs) of 1.39 and 0.77, respectively. The shorter C–B bond and higher WBI are consistent with multiple bonding, whereas the longer C–B bond is described as a single bond that is close to reported tricoordinate boron compounds with ^Me^*o*Cb substituents [HB^Me^*o*Cb_2_ = 1.557(5) and 1.567(5) Å, BrB^Me^*o*Cb_2_ = 1.596(7) and 1.601(7) Å].^53^ Notably, the adjacent cage C–C bond between the *ortho* carbons is elongated to 1.76 Å on the side bearing the shorter C–B bond indicative of weakening the C–C linkage, compared to 1.66 Å on the longer C–B bonded side. The lengthening of the C–C bond of *ortho*-carborane species from negative hyperconjugative interactions with an electron rich atom is consistent with literature precedent.^62–67^ Together, the geometric metrics and WBI/NBO analysis support that one C–B bond is π-reinforced and engages in negative hyperconjugative donation with the carborane, while the other does not have multiple bond character. We have labeled the geometry as linear as the optimized angle only deviates slightly from perfect linearity with a C–B–C angle of 176.2°. This bonding description is consistent for the other two anions B^Ph^*o*Cb_2_^−^ and B^TMS^*o*Cb_2_^−^.

**Fig. 4:**
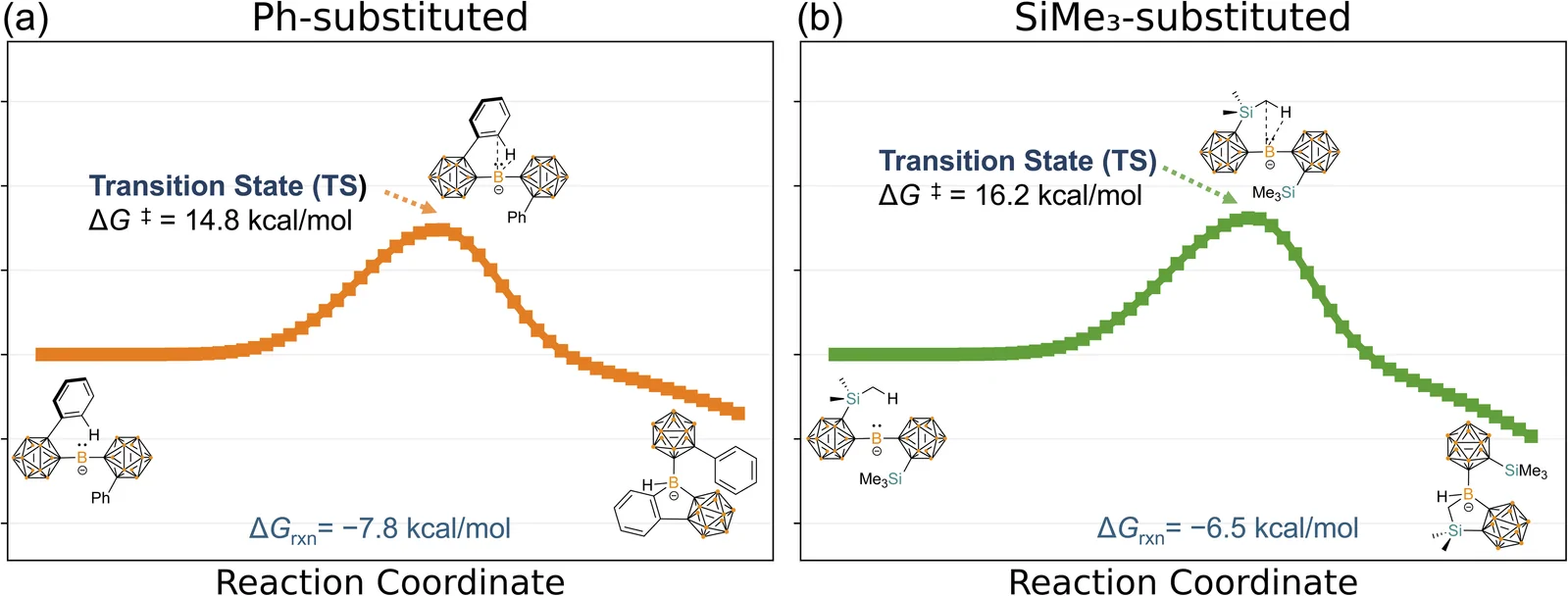
Free-energy diagrams for intramolecular C–H borylation of (a) B^Ph^*o*Cb_2_^−^ and (b) B^TMS^*o*Cb_2_^−^.

To evaluate the C–H borylation reactivity pathways of B^Ph^*o*Cb_2_^−^ and B^TMS^*o*Cb_2_^−^, we computed the five-membered transition states (TS1) from the ground state linear singlet electronic configurations (Fig. 4). Both proceed *via* a concerted insertion in which the boron lone pair engages a proximal C–H unit, forging a B–C and a B–H bond with a Δ*G*^‡^ of 14.8 for Ph and 16.2 kcal mol^−1^ for SiMe_3_. The reactions are exergonic with Δ*G*_rxn_ of −7.8 (Ph) and −6.5 kcal mol^−1^ (SiMe_3_). Thus, the kinetic barriers support the singlet boryl anion pathways for intramolecular C–H insertion.

**Fig. 5:**
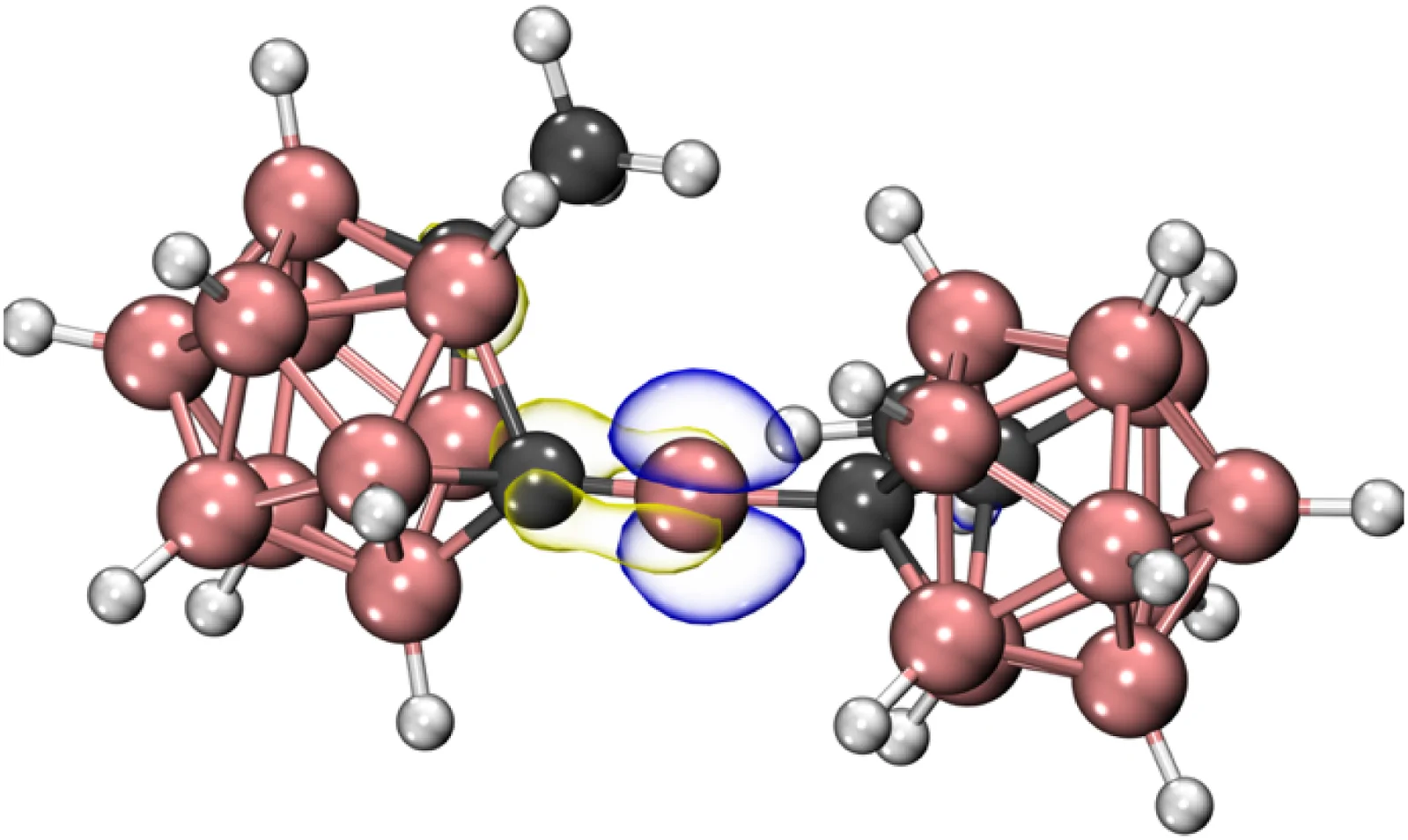
Computed condensed Fukui functions of B^Me^*o*Cb_2_^−^. The isosurfaces of *f*_B_^+^(*r*) = *ρ*(*N* + 1) − *ρ*(*N*) and *f*_B_^−^(*r*) = *ρ*(*N*) − *ρ*(*N* − 1) represented by blue and yellow lobes, respectively, are calculated following the reaction pathway with tetrachloroquinone (isosurface value = 0.01), highlighting boron-centered acceptor and donor response relevant to O → B and B → π* interactions in the cycloaddition pathway. The B, C, H atoms are represented as the pink, grey, white balls, respectively.

The B^Me^*o*Cb_2_^−^ anion engages in [4 + 1] cycloaddition reactivity with tetrachloroquinone while cycloaddition reactivity of the *N*-heterocyclic boryl (NHB) species have not been reported. To rationalize this difference, we compared frontier orbital energetics and condensed Fukui indices at boron to assess the relative nucleophilicity and electrophilicity. Computing frontier orbital energetics and condensed Fukui indices reveal the HOMO primarily lies on the boron of B^Me^*o*Cb_2_^−^ at 1.8 eV (*E*_HOMO_) while the lowest-energy unoccupied molecular orbital with dominant boron acceptor character lies at 3.7 eV (*E*_acc_, LUMO+2), leading to an ambiphilicity gap of 1.9 eV (Δ*E* = *E*_acc_ − *E*_HOMO_). In contrast, the computed five-membered NHB boryl anion with an unsaturated backbone (NHB1) and the saturated NHB variant (NHB2) exhibit comparable σ-donor HOMO energies (*E*_HOMO_ = 1.4 eV for NHB1 and 1.5 eV NHB2) but substantially higher boron-centered acceptor orbitals (*E*_acc_ = 5.8 eV, NHB1, LUMO+4; 5.4 eV NHB2, LUMO+2), resulting in much larger donor–acceptor separations (Δ*E* = 4.4 NHB1, 3.9 NHB2*c.f.* B^Me^*o*Cb_2_^−^ 1.9 eV). Consistent with the boron centered frontier molecular orbitals, condensed Fukui functions at boron indicate that B^Me^*o*Cb_2_^−^ retains appreciable acceptor character (*f*_B_^+^ = 0.10) in addition to nucleophilicity (*f*_B_^−^ = 0.35), yielding an ambiphilic boron center based on a negative Δ*f*_B_ (Δ*f*_B_ = *f*_B_^+^ − *f*_B_^−^ = −0.25). The visual representation of the computed condensed phase Fukui functions with *f*_B_^+^ and *f*_B_^−^ are illustrated in Fig. 5. On the other hand, the NHB analogues exhibit markedly smaller acceptor character (*f*_B_^+^ = 0.01 NHB1, 0.03 NHB2) and a strongly nucleophile-biased dual descriptor (*f*_B_^−^ = 0.35 NHB1, 0.36 NHB2; Δ*f*_B_ = −0.34 NHB1, −0.33 NHB2), consistent with the high-lying boron acceptor orbitals. Collectively, these descriptors indicate enhanced donor/acceptor accessibility at boron in B^Me^*o*Cb_2_^−^, enabling paired donor (B → π*) and acceptor (O → B) interactions required for a cheletropic-type [4 + 1] closure, while NHB systems remain predominantly nucleophilic reagents with inaccessible boron acceptor orbitals, thereby suppressing analogous cycloaddition pathways.

## Conclusions

In summary, the reduction of bis(*ortho*-carboranyl)bromoboranes with potassium resulted in the isolation of anionic five-membered boracycles by boryl anion insertion into C_sp^2^_–H and C_sp^3^_–H bonds for *ortho*-phenyl and *ortho*-trimethylsilyl substituted species. For the methyl substituted species, boryl anion reactivity could be harnessed to access a copper–boryl complex and cycloaddition with a diketone. DFT analyses reveal the ground state is a linear singlet with considerable density at boron and multiple bonding with one of the carbons of the carborane through negative hyperconjugation. The phenyl and trimethylsilyl analogues undergo facile C–H insertion precluding reactivity studies. The methyl-substituted species faces a much higher C–H insertion barrier that enabled boryl anion reactivity studies. These results indicate that the unique electron withdrawing nature and bulk of carboranes make them effective substituents for the generation of the reactive boryl anions with an electronic structure that is considerably different from reported counterparts.

## Author contributions

The manuscript was written through contributions of all authors. All authors have given approval to the final version of the manuscript. K. V. and R. A. T. carried out the synthetic experiments and single crystal X-ray diffraction analyses. B. Y. performed the DFT calculations and composed the related text. C. D. M. supervised the project. C. D. M., K. V., and R. A. T. wrote the manuscript with edits from B. Y.

## Conflicts of interest

There are no conflicts to declare.

## Supplementary Material

SC-017-D6SC03158G-s001

SC-017-D6SC03158G-s002

SC-017-D6SC03158G-s003

## Data Availability

CCDC 2482457, 2482458, 2482460, 2482461, 2504475 and 2520596 contain the supplementary crystallographic data for this paper.^68*a*–*f*^ The data supporting the results of this study are available in the supplementary information (SI) of this article. Supplementary information: experimental procedures, NMR spectra, computational details, and X-ray crystallographic data. See DOI: https://doi.org/10.1039/d6sc03158g.

## References

[cit1] Herrmann W. A., Köcher C. (1997). Angew. Chem., Int. Ed..

[cit2] Hahn F. E., Jahnke M. C. (2008). Angew. Chem. Int. Ed..

[cit3] Melaimi M., Soleilhavoup M., Bertrand G. (2010). Angew. Chem. Int. Ed..

[cit4] Schuster O., Yang L., Raubenheimer H. G., Albrecht M. (2009). Chem. Rev..

[cit5] Hopkinson M. N., Richter C., Schedler M., Glorius F. (2014). Nature.

[cit6] Zhao Q., Dewhurst R. D., Braunschweig H., Chen X. (2019). Angew. Chem. Int. Ed..

[cit7] Terabayashi T., Kajiwara T., Yamashita M., Nozaki K. (2009). J. Am. Chem. Soc..

[cit8] Dettenrieder N., Schädle C., Maichle-Mössmer C. C., Sirsch P., Anwander R. (2014). J. Am. Chem. Soc..

[cit9] Cardin D., Cetinkaya B., Lappert M. (1972). Chem. Rev..

[cit10] Herrmann W. A., Elison M., Fischer J., Köcher C., Artus G. R. (1995). Angew. Chem., Int. Ed..

[cit11] Gandelman M., Rybtchinski B., Ashkenazi N., Gauvin R. M., Milstein D. (2001). J. Am. Chem. Soc..

[cit12] Aldridge S., Coombs D. L. (2004). Coord. Chem. Rev..

[cit13] Guo X., Lin Z. (2024). Chem. Sci..

[cit14] Waltz K. M., Hartwig J. F. (1997). Science.

[cit15] Irvine G. J., Lesley M. G., Marder T. B., Norman N. C., Rice C. R., Robins E. G., Roper W. R., Whittell G. R., Wright L. J. (1998). Chem. Rev..

[cit16] Segawa Y., Yamashita M., Nozaki K. (2006). Science.

[cit17] Segawa Y., Suzuki Y., Yamashita M., Nozaki K. (2008). J. Am. Chem. Soc..

[cit18] Segawa Y., Yamashita M., Nozaki K. (2007). Angew. Chem., Int. Ed..

[cit19] Lu W., Hu H., Li Y., Ganguly R., Kinjo R. (2016). J. Am. Chem. Soc..

[cit20] Yamashita M. (2011). Bull. Chem. Soc. Jpn..

[cit21] Romeo L. J., Kaur A., Wilson D. J., Martin C. D., Dutton J. L. (2019). Inorg. Chem..

[cit22] Kajiwara T., Terabayashi T., Yamashita M., Nozaki K. (2008). Angew. Chem..

[cit23] Braunschweig H., Chiu C.-W., Radacki K., Kupfer T. (2010). Angew. Chem. Int. Ed..

[cit24] Babula D. J., Charman R. S., Jerome T. H., Horsley Downie T. M., Mahon M. F., Liptrot D. J. (2023). Eur. J. Inorg. Chem..

[cit25] Protchenko A. V., Vasko P., Fuentes M. Á., Hicks J., Vidovic D., Aldridge S. (2021). Angew. Chem. Int. Ed..

[cit26] Campos J., Aldridge S. (2015). Angew. Chem. Int. Ed..

[cit27] Yamashita M., Suzuki Y., Segawa Y., Nozaki K. (2007). J. Am. Chem. Soc..

[cit28] Okuno Y., Yamashita M., Nozaki K. (2011). Angew. Chem..

[cit29] Liu Y., Zhang J., Xie Z. (2025). Nat. Commun..

[cit30] Ruiz D. A., Ung G., Melaimi M., Bertrand G. (2013). Angew. Chem., Int. Ed..

[cit31] Wentz K. E., Molino A., Freeman L. A., Dickie D. A., Wilson D. J., Gilliard Jr R. J. (2021). Inorg. Chem..

[cit32] Wentz K. E., Molino A., Freeman L. A., Dickie D. A., Wilson D. J., Gilliard Jr R. J. (2022). J. Am. Chem. Soc..

[cit33] Wentz K. E., Molino A., Freeman L. A., Dickie D. A., Wilson D. J., Gilliard Jr R. J. (2022). Inorg. Chem..

[cit34] Feng Z., Kinjo R. (2025). Chem.

[cit35] Akram M. O., Martin C. D. (2025). Chem.

[cit36] Hollister K. K., Yang W., Mondol R., Wentz K. E., Molino A., Kaur A., Dickie D. A., Frenking G., Pan S., Wilson D. J., Gilliard Jr R. J. (2022). Angew. Chem..

[cit37] Gärtner A., Marek M., Arrowsmith M., Auerhammer D., Radacki K., Prieschl D., Dewhurst R. D., Braunschweig H. (2021). Chem.–Eur. J..

[cit38] Zhang C., Wang J., Zhang X., Dabringhaus P., Shi W., Qian K., Deng C.-L., Cummins C. C., Gilliard Jr R. J. (2025). J. Am. Chem. Soc..

[cit39] Grigsby W. J., Power P. P. (1996). J. Am. Chem. Soc..

[cit40] Akram M. O., French K. A., Shuford K. L., Martin C. D. (2025). J. Am. Chem. Soc..

[cit41] Akram M. O., Martin C. D., Dutton J. L. (2023). Inorg. Chem..

[cit42] Akram M. O., Tidwell J. R., Dutton J. L., Martin C. D. (2022). Angew. Chem..

[cit43] Fisher S. P., Tomich A. W., Lovera S., Kleinsasser J. F., Guo J., Asay M., Nelson H., Lavallo V. (2019). Chem. Rev..

[cit44] Grimes R. N. (2015). Dalton Trans..

[cit45] Huh J. O., Kim H., Lee K. M., Lee Y. S., Do Y., Lee M. H. (2010). Chem. Commun..

[cit46] Liu Y., Dong W., Li Z. H., Wang H. (2021). Chem.

[cit47] Ranasinghe S., Li Y., Andrews M. E., Akram M. O., Thornton R. A., Martin C. D. (2025). Chem. Commun..

[cit48] Tan X., Wang X., Li Z. H., Wang H. (2022). J. Am. Chem. Soc..

[cit49] Xiang L., Wang J., Matler A., Ye Q. (2024). Chem. Sci..

[cit50] Zhang C., Wang J., Lin Z., Ye Q. (2022). Inorg. Chem..

[cit51] Zhang C., Wang J., Su W., Lin Z., Ye Q. (2021). J. Am. Chem. Soc..

[cit52] Li Y., Tamizmani M., Akram M. O., Martin C. D. (2024). Chem. Sci..

[cit53] Akram M. O., Tidwell J. R., Dutton J. L., Martin C. D. (2023). Angew. Chem. Int. Ed..

[cit54] Vashisth K., Martin C. D. (2025). Inorg. Chem..

[cit55] Laitar D. S., Müller P., Sadighi J. P. (2005). J. Am. Chem. Soc..

[cit56] Silva Valverde M. F., Schweyen P., Gisinger D., Bannenberg T., Freytag M., Kleeberg C., Tamm M. (2017). Angew. Chem. Int. Ed..

[cit57] Rosokha S., Dibrov S., Rosokha T., Kochi J. (2006). Photochem. Photobiol. Sci..

[cit58] Weigend F., Ahlrichs R. (2005). Phys. Chem. Chem. Phys..

[cit59] Weigend F. (2006). Phys. Chem. Chem. Phys..

[cit60] Chai J.-D., Head-Gordon M. (2008). Phys. Chem. Chem. Phys..

[cit61] Marenich A. V., Cramer C. J., Truhlar D. G. (2009). J. Phys. Chem. B.

[cit62] Li J., Pang R., Li Z., Lai G., Xiao X. Q., Müller T. (2019). Angew. Chem. Int. Ed..

[cit63] Krebs J., Brändler L., Krummenacher I., Friedrich A., Braunschweig H., Finze M., Curchod B. F., Marder T. B. (2024). Chem.–Eur. J..

[cit64] Krebs J., Haehnel M., Krummenacher I., Friedrich A., Braunschweig H., Finze M., Ji L., Marder T. B. (2021). Chem.–Eur. J..

[cit65] Gao Y., Szathmári B., Buzsáki D., Kelemen Z. (2025). Chem.–Eur. J..

[cit66] Humphries A. L., Tellier G. A., Smith M. D., Chianese A. R., Peryshkov D. V. (2024). J. Am. Chem. Soc..

[cit67] Riffle J. R., Hemingway T. M., Smith M. D., Peryshkov D. V. (2024). Dalton Trans..

[cit68] (a) CCDC 2482457: Experimental Crystal Structure Determination, 2026, 10.5517/ccdc.csd.cc2pb68j

