# Supplementary material for: Transient bis(carboranyl)boryl anions
Source: Chem Sci. 2026 Jun 25;17(33):15762–7. doi: 10.1039/d6sc03158g (PMC13356875; doi:10.1039/d6sc03158g)
Supplement: Supplementary file 1 [file SC-017-D6SC03158G-s001.pdf]

Supplementary Information (SI) for Chemical Science.
This journal is © The Royal Society of Chemistry 2026

Supporting Information for:

Transient Bis(Carboranyl)boryl Anions

Kanika Vashisth[a,‡], Ragene A. Thornton[a,‡], Bohak Yoon[a,], and Caleb D. Martin[a,*]*

Baylor University, Department of Chemistry and Biochemistry, One Bear Place #97348, Waco, TX 76798, USA.

Author to address correspondence: Caleb D. Martin (caleb_d_martin@baylor.edu)

Table of Contents

1. *General Considerations* *S3*
2. *Experimental Section* *S4*
    NMR spectra *S9*
    X-ray Crystallographic details *S30*
3. *Theoretical Calculations* *S31*
4. *References* *S53*

1. General Considerations:

All manipulations were performed under an inert atmosphere in a nitrogen filled MBraun Unilab glove box or using standard Schlenk techniques. $CDCl_3$ and C_6D_6 for NMR spectroscopy were purchased from Cambridge Isotope Laboratories, Inc., dried by stirring for 5 days over CaH_2, distilled, and stored over 3 Å molecular sieves. All other solvents were purchased from commercial sources as anhydrous grade, dried further using a JC Meyer Solvent System with dual columns packed with solvent-appropriate drying agents, and stored over 3 or 4 Å molecular sieves. Toluene was dried further by distilling over Na/benzophenone and stored in a vessel with a potassium mirror. Trimethylsilyl-*ortho*-carborane (^{TMS}oCb), bis(1-phenyl-*ortho*-carboranyl)boron bromide (BrB^{Ph}oCb$_2$), bis(1-methyl-*ortho*-carboranyl)boron bromide (BrB^{Me}oCb$_2$), and $CuPPh_3Cl$ were prepared by the literature procedures.[1-4] Potassium metal, *n*BuLi (2.5 M in hexanes), BBr_3, 18-crown-6, and tetrachloro-quinone were purchased from commercial sources and used without further purification. Multinuclear NMR data (^{1}H, ^{13}C{^{1}H}, ^{29}Si{^{1}H}, ^{11}B, ^{11}B{^{1}H}, ^{31}P{^{1}H}) were recorded on a Bruker Avance III HD 400 MHz or 600 MHz instrument. High Resolution mass spectra (HRMS) were obtained in the Baylor University Mass Spectrometry Center on a Thermo Scientific LTQ Orbitrap Discovery spectrometer using +ESI and −ESI. Melting points were measured with a Laboratory device MEL-TEMP II capillary melting point apparatus and are uncorrected. FT-IR spectra were recorded on a Bruker Alpha ATR FT-IR spectrometer on solid samples. Single crystal X-ray diffraction data were collected on a Bruker Apex III-CCD detector using Mo-Kα radiation (λ = 0.71073 Å) or a Rigaku XtaLAB Synergy-S system using Cu radiation (λ = 1.54184 Å). Crystals were selected under paratone oil, mounted on MiTeGen micromounts, and immediately placed in a cold stream of N_2. Structures were solved and refined using SHELXTL and figures produced using OLEX2.[5,6]

2. Experimental Section

K[1]: Potassium metal (8.5 mg, 0.22 mmol) was added to a toluene (3 mL) solution of BrB^{Ph}*o*Cb_2 (45.9 mg, 0.0860 mmol) at 23 °C and stirred for 4 h. The mixture was passed through a pad of celite and the solvent removed under reduced pressure. The residue was washed with 1:2 toluene/*n*-pentane (2 × 1 mL) to obtain a beige solid. Due to the insolubility of the product, it could not be characterized by solution NMR spectroscopy. Yield: 76%, 32 mg; mp: 112-113 °C. HRMS (–ESI): calculated for $[C_{16}B_{21}H_{30}]^{-}[M]^{-}$ 449.4449 found 449.4410.

K-crown[1]: 18-Crown-6 (20.5 mg, 0.0780 mmol) was added to a toluene (1 mL) solution of **K[1]** (38.1 mg, 0.0780 mmol) and stirred for an hour at 23 °C. The volatiles were removed in vacuo. The residue was washed with 1:2 toluene/*n*-pentane (2 × 1 mL) to obtain a white solid. Single crystals for X-ray diffraction studies were grown from a concentrated toluene/*n*-pentane solution of **K-crown[1]** at –35 °C. Yield: 59%, 47 mg; mp: 128-130 °C. ^{1}H NMR (600 MHz, $CDCl_3$): δ = 7.69-7.60 (m, 3H), 7.25-7.21 (m, 3H), 7.08 (td, *J* = 12, 2 Hz, 1H), 7.05-7.01 (m, 1H), 6.91 (t, *J* = 9 Hz, 1H), 3.57-1.18 (m, 69H) ppm; $^{13}C\{^{1}H\}$ NMR (151 MHz, $CDCl_3$): δ = 143.6, 135.2, 132.2, 131.6, 129.2, 128.9, 127.7, 126.3, 124.2, 120.2, 88.1, 86.8, 70.3 ppm; $^{11}B\{^{1}H\}$ NMR (193 MHz, $CDCl_3$): δ = –2.1 (s), –4.9 to –12.8 (m, cage *o*CbH-B) ppm; ^{11}B NMR (193 MHz, $CDCl_3$): δ = –2.1 (d, *J* = 74 Hz), –5.3 to –13.0 (m, cage *o*CbH-B) ppm; HRMS (+ESI): calculated for $[C_{12}H_{24}O_6K]^{+}[M]^{+}$ 303.1202 found 303.1204; (–ESI): calculated for $[C_{16}B_{21}H_{30}]^{-}[M]^{-}$ 449.4449 found 449.4410.

BrB^{TMS}*o*Cb_2: ^{n}BuLi (2.0 mL, 5.0 mmol, 2.5 M in hexanes) was added dropwise to a toluene (15 mL) solution of ^{TMS}*o*Cb (1.1 g, 5.0 mmol) at –78 °C resulting in a yellow solution. The reaction was stirred for 40 minutes at –78 °C giving a white slurry. The cold bath was removed and the

mixture stirred for 4 h. A toluene (5 mL) solution of BBr_3 (0.24 mL, 2.5 mmol) was added via cannula at –78 °C and stirred for 40 minutes. The cold bath was removed and the reaction stirred for 3 days at 23 °C. Toluene (10 mL) was added and the reaction mixture passed through a pad of celite. The volatiles were removed under reduced pressure, the crude solid washed with 1:2 ether/*n*-pentane (3 × 2 mL), and dried in vacuo to obtain a white powder. Single crystals for X-ray diffraction studies were grown from a concentrated *n*-pentane solution of **BrB^{TMS}*o*Cb_2** at –35 °C. Yield: 46%, 0.6263 g; mp: 186-188 °C. ^1H NMR (400 MHz, C_6D_6): δ = 3.68-1.97 (m, 20H), 0.00 (s, 18H) ppm; $^{13}C\{^1H\}$ NMR (101 MHz, C_6D_6): δ = 83.1, 1.7 ppm; $^{11}B\{^1H\}$ NMR (128 MHz, C_6D_6): δ = 64.1 (s, central boron), 5.7 (s), 1.8 (s), –3.8 to –8.8 (m, cage *o*CbH-B) ppm; ^{11}B NMR (128 MHz, C_6D_6): δ = 64.2 (s, central boron), 5.7 (d, *J* = 152 Hz), 1.7 (d, *J* = 147 Hz), –3.8 to –8.7 (m, cage *o*CbH-B) ppm; $^{29}Si\{^1H\}$ NMR (79 MHz, C_6D_6): 11.6 (s) ppm; HRMS (−ESI): calculated for $[C_{10}H_{39}B_{21}Si_2Br]^-[M+H]^-$ 521.3873 found 521.3855.

K[2]: Potassium metal (5.9 mg, 0.15 mmol) was added to a solution of BrB^{TMS}*o*Cb_2 (33.1 mg, 0.0600 mmol) in 3 mL of toluene at 23 °C. The reaction mixture was stirred for 2 h. The mixture was passed through a pad of celite and the solvent removed under reduced pressure. The residue was washed with *n*-pentane (2 × 1 mL) to obtain a yellow solid. Single crystals were grown from storing a mixture of THF and toluene solution of **K[2]** at –35 ºC. Yield: 96%, 29 mg; mp: 80-81 °C. ^1H NMR (600 MHz, C_6D_6): δ = 3.07-2.29 (m, 20H), 0.56 (d, *J* = 6 Hz, 1H), 0.54 (d, *J* = 6 Hz, 1H), 0.50 (s, 9H), 0.37 (s, 3H), 0.31 (s, 3H) ppm; $^{13}C\{^1H\}$ NMR (151 MHz, C_6D_6): δ = 82.6, 82.0, 1.9, –0.7, –1.3, –2.0 ppm; $^{11}B\{^1H\}$ NMR (193 MHz, C_6D_6): δ = 1.1 to –15.0 (m, cage *o*CbH-B) ppm; ^{11}B NMR (193 MHz, C_6D_6): δ = 0.4 to –15.4 (m, cage *o*CbH-B) ppm; $^{29}Si\{^1H\}$ NMR (119

MHz, C_6D_6): 20.4 (s), 6.9 (s) ppm; HRMS (–ESI): calculated for $[C_{10}H_{38}B_{21}Si_2]^-[M]^-$ 441.4606 found 441.4613.

K-crown[2]: 18-crown-6 (0.025 mmol, 6.7 mg) was added to a toluene (1 mL) solution of **K[2]** (0.0250 mmol, 12.3 mg) and stirred for an hour at 23 °C. The volatiles were removed under reduced pressure and the crude washed with *n*-pentane (2 × 1 mL), and dried in vacuo. Yield: 86%, 16 mg; mp: 88-90 °C. ^1H NMR (600 MHz, C_6D_6): δ = 3.40-2.18 (m, 44H), 0.61 (d, *J* = 6 Hz, 1H), 0.59 (d, *J* = 6 Hz, 1H), 0.54 (s, 9H), 0.38 (s, 3H), 0.35 (s, 3H) ppm; $^{13}C\{^1H\}$ NMR (151 MHz, C_6D_6): δ = 81.7, 81.3, 70.0, 2.1, 1.4, –0.5, –1.1, –2.0 ppm; $^{11}B\{^1H\}$ NMR (193 MHz, C_6D_6): δ = 1.1 to –15.0 (m, cage *o*CbH-B) ppm; ^{11}B NMR (193 MHz, C_6D_6): δ = 0.4 to –15.4 (m, cage *o*CbH-B) ppm; $^{29}Si\{^1H\}$ NMR (119 MHz, C_6D_6): 19.8 (s), 6.2 (s) ppm; HRMS (+ESI): calculated for $[C_{12}H_{24}O_6K]^+[M]^+$ 303.1204 found 303.1203; (–ESI): calculated for $[C_{10}B_{21}H_{38}Si_2]^-[M]^-$ 441.4606 found 441.4608.

3: Potassium metal (63.4 mg, 1.62 mmol) was added to a solution of BrB^{Me}*o*Cb_2 (263 mg, 0.649 mmol) in 10 mL of toluene in a pressure tube. The pressure tube was heated to 110 ºC for 6 h. The reaction mixture was passed through a pad of celite to obtain a yellow solution. A toluene (3 mL) solution of Ph_3PCuCl (0.145 mmol, 51.3 mg) was added to 2.2 mL (0.145 mmol) aliquot of the filtrate and the reaction stirred for 45 min at 23 °C. The volatiles were removed in vacuo and the pale yellow solid washed with cold *n*-pentane (3 × 5 mL). Crystals for X-ray diffraction were grown by vapor diffusion of *n*-pentane into toluene solution of **3**. Yield: 66%, 24 mg; dp 141 °C; ^1H NMR (400 MHz, C_6D_6): δ = 7.29-7.18 (m, 5H), 6.99-6.95 (m, 10H), 2.94-2.32 (m, 20H), 1.82 (s, 6H) ppm; $^{13}C\{^1H\}$ NMR (101 MHz, C_6D_6): δ = 134.2 (d, *J* = 13 Hz), 130.8, 129.2, 79.3, 25.3 ppm; $^{11}B\{^1H\}$ NMR (128 MHz, C_6D_6): δ = 23.4 (br, s, central boron), –1.4 (s), –4.7 (s), –6.2 (s),

–8.3 to –12.6 (m, cage *o*CbH-B) ppm; ^{11}B NMR (128 MHz, C_6D_6): δ = 24.0 (br, s, central boron), –1.3 (d, *J* = 111 Hz), –4.6 (d, *J* = 99 Hz), –5.9 to –13.1 (m, cage *o*CbH-B); ^{31}P{^{1}H} NMR (162 MHz, C_6D_6): δ = –4.2 (br, s) ppm. HRMS (+ESI): calculated for $[C_{36}H_{30}CuP_2]^+[M–B_{21}C_6H_{26}]^+$ 587.1119 found 587.1006; HRMS (–ESI): calculated for $[C_{13}H_{34}B_{21}]^–[(M+C_7H_8)–CuPPh_3]^–$ 417.4735 found 417.4760.

4: Potassium metal (24.1 mg, 0.617 mmol) was added to a solution of BrB^{Me}*o*Cb_2 (100 mg, 0.247 mmol) in 4 mL of toluene in a pressure tube. The pressure tube was heated to 110 ºC for 7 h. The reaction mixture was passed through a pad of celite to obtain a yellow solution. 18-crown-6 (86.9 mg, 0.329 mmol) and tetrachloro-*o*-benzoquinone (80.8 mg, 0.329 mmol) were added to a 2.1 mL aliqot of the filtrate (0.329 mmol) that was heated to 110 ºC for 6 h. The reaction mixture was filtered through a pad of celite and the volatiles stripped in vacuo. The residue was washed with *n*-pentane (2 × 1 mL) to obtain a yellow oil. Single crystals for X-ray diffraction studies were grown from a vapor diffusion of concentrated ether solution of **4** into hexane at room temperature. Yield: 49%, 178 mg. ^{1}H NMR (600 MHz, $CDCl_3$): δ = 3.63 (s, 48H), 2.72-1.57 (m, 26H) ppm; ^{13}C{^{1}H} NMR (151 MHz, $CDCl_3$): δ = 148.0, 120.8, 112.3, 70.5, 61.7, 26.1 ppm; ^{11}B{^{1}H} NMR (193 MHz, $CDCl_3$): δ = 14.4 (s, central boron), –1.7 (s), –6.7 (s), –9.1 to –12.7 (m, cage *o*CbH-B) ppm; ^{11}B NMR (193 MHz, $CDCl_3$): δ = 14.4 (s, central boron), –1.7 (d, *J* = 100 Hz), –6.7 (d, *J* = 93 Hz), –8.7 to –13.1 (m, cage *o*CbH-B) ppm. HRMS (+ESI): calculated for $[C_{12}H_{24}O_6K]^+[M]^+$ 303.1202 found 303.1174; (–ESI): calculated for $[C_{12}B_{21}H_{26}O_2Cl_4]^–[M]^–$ 571.2751 found 571.2762.

[K][H_2B^{Me}*o*Cb_2]: Attempted isolation of $[B^{Me}$*o*$Cb_2]^–$ resulted in formation of **[K][H_2B^{Me}*o*Cb_2]**. Single crystals for X-ray diffraction studies were grown from a vapor diffusion of concentrated

dichloromethane solution of **[K][H_2B^{Me}*o*Cb_2]** into toluene at room temperature. $^{11}B\{^1H\}$ NMR (128 MHz, C_6D_6): δ = –1.4 to –12.6 (m), –15.3 (s, central boron) ppm; ^{11}B NMR (128 MHz, C_6D_6): –0.8 to –13.2 (m), –15.4 (t, *J* = 86 Hz, central boron) ppm.

Figure S-1: ^{1}H NMR (600 MHz) spectrum of **K-crown[1]** in $CDCl_3$ (* toluene).

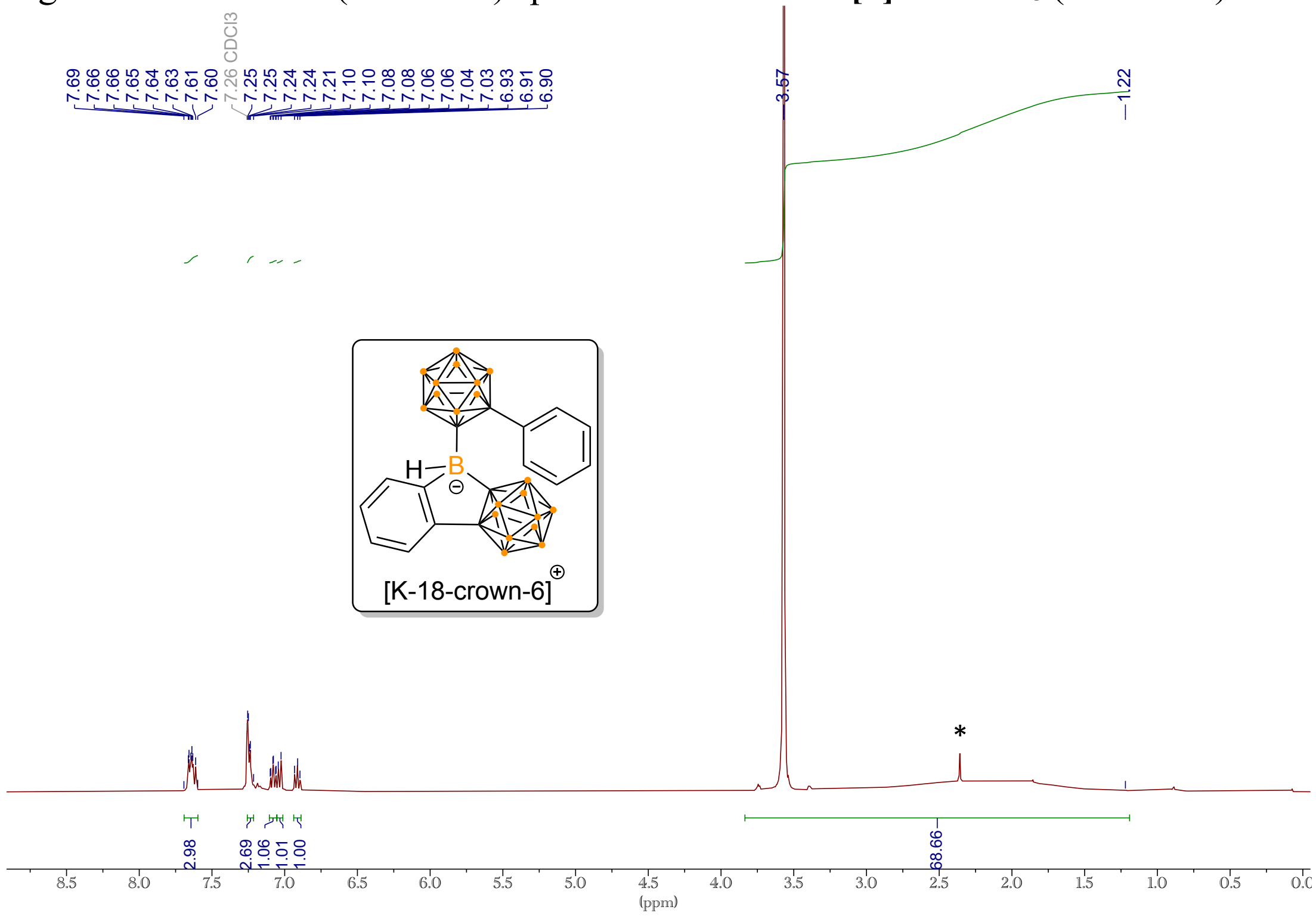

Figure S-2: Expanded aryl region ^{1}H NMR (600 MHz) spectrum of **K-crown[1]** in $CDCl_3$.

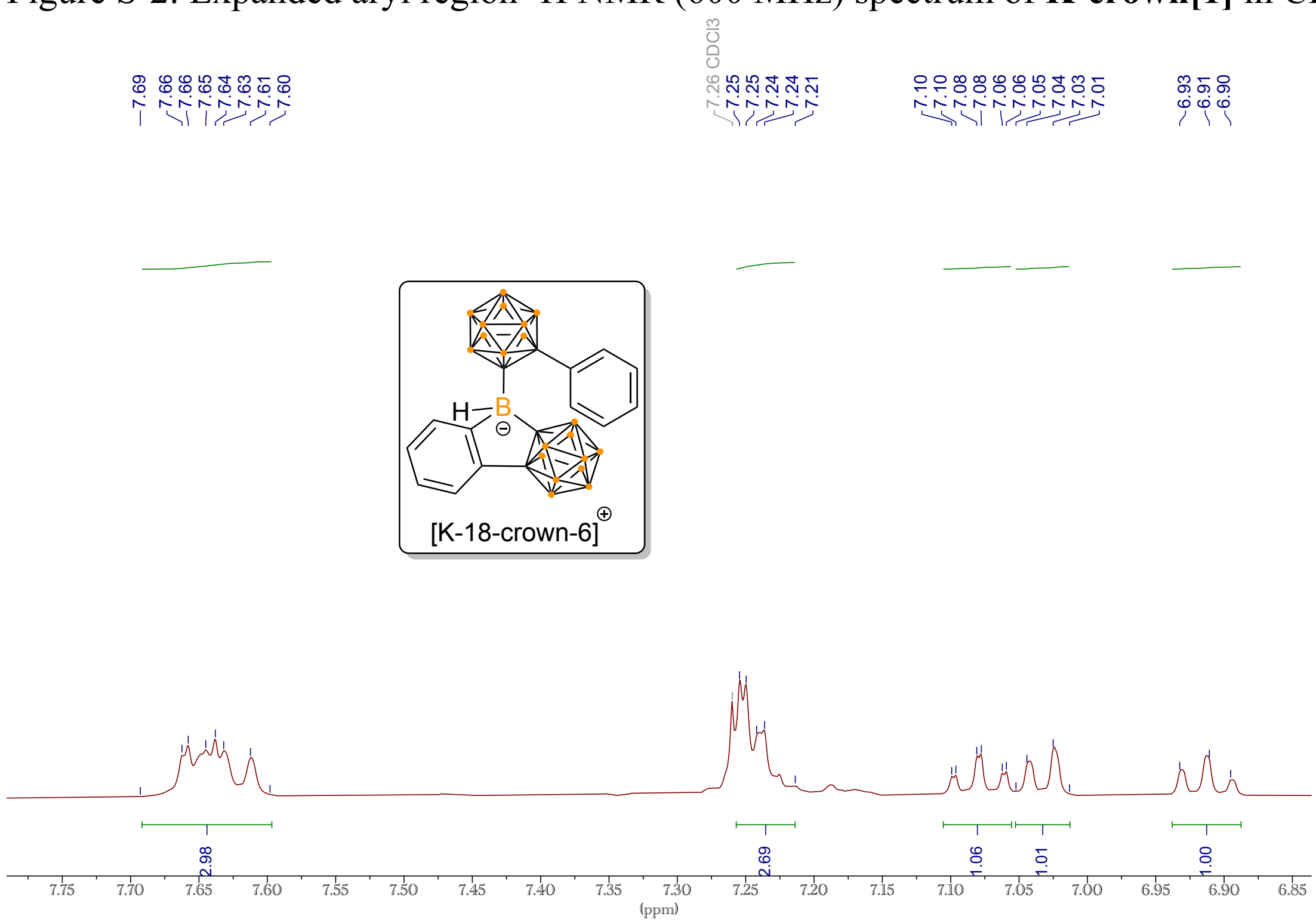

Figure S-3: $^{13}C\{^{1}H\}$ NMR (151 MHz) spectrum of **K-crown[1]** in $CDCl_3$.

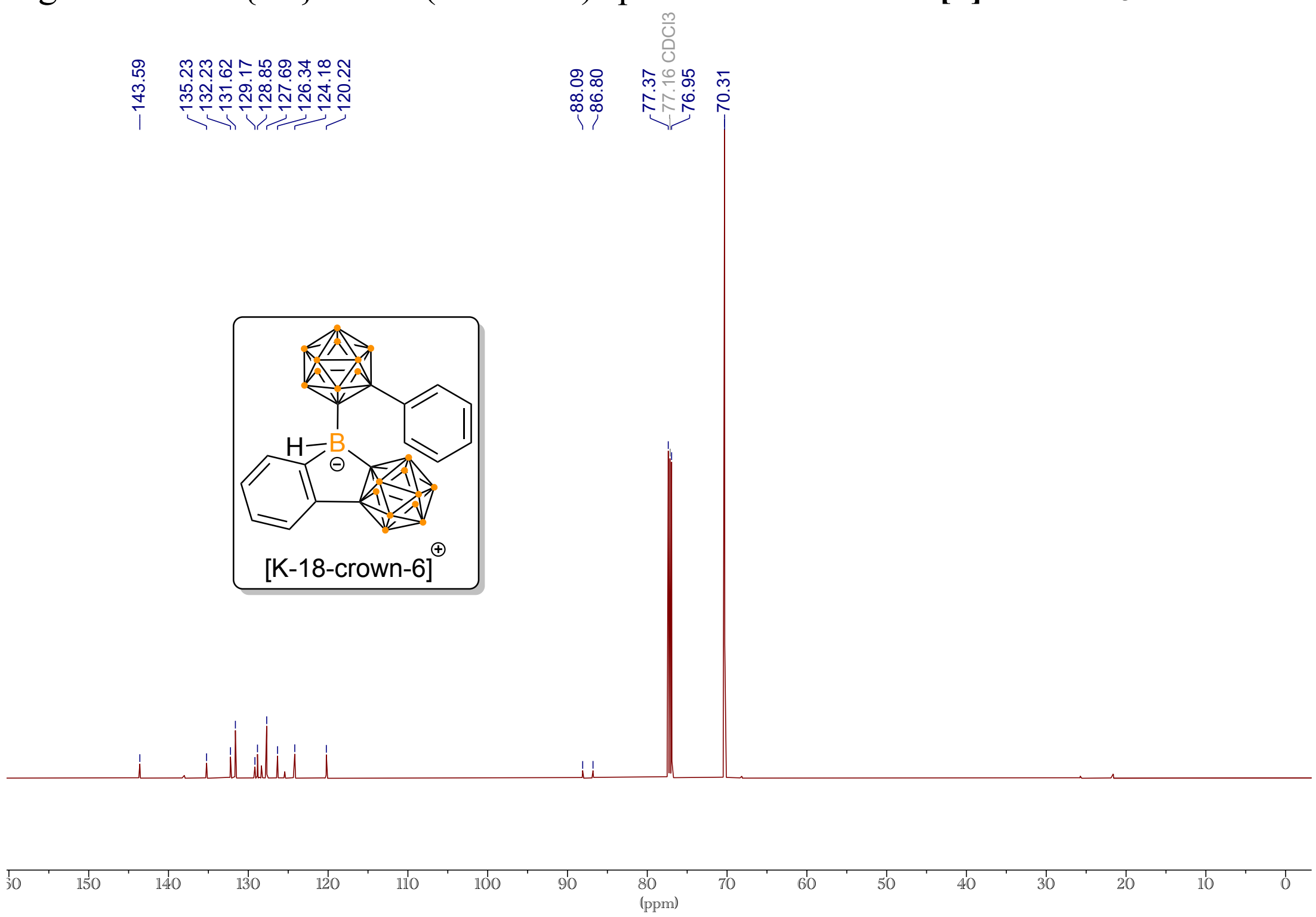

Figure S-4: $^{11}B\{^{1}H\}$ NMR (193 MHz) spectrum of **K-crown[1]** in $CDCl_3$.

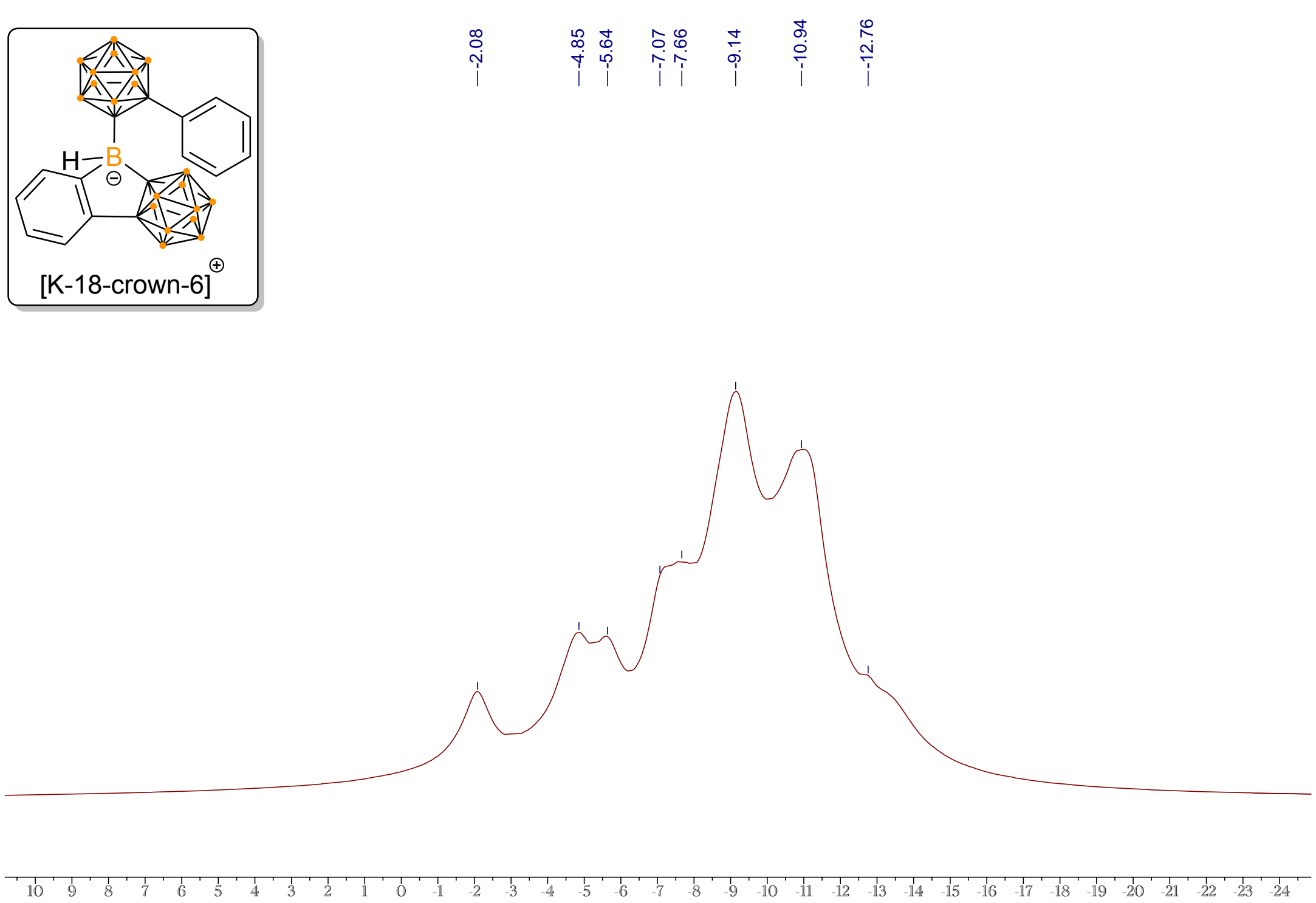

Figure S-5: ^{11}B NMR (193 MHz) spectrum of **K-crown[1]** in $CDCl_3$.

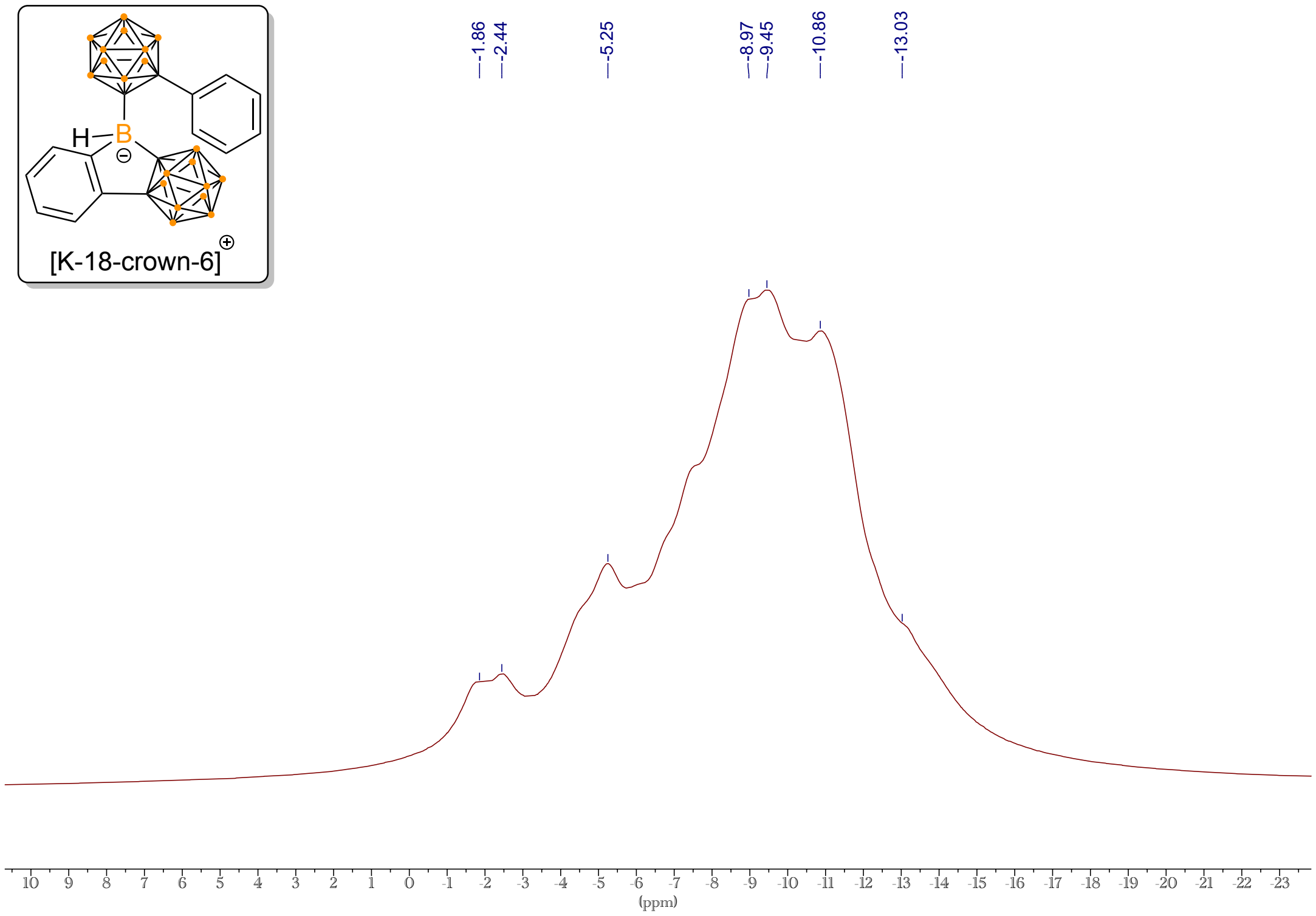

Figure S-6: ^{1}H NMR (400 MHz) spectrum of **BrB^{TMS}*o*Cb$_2$** in C_6D_6.

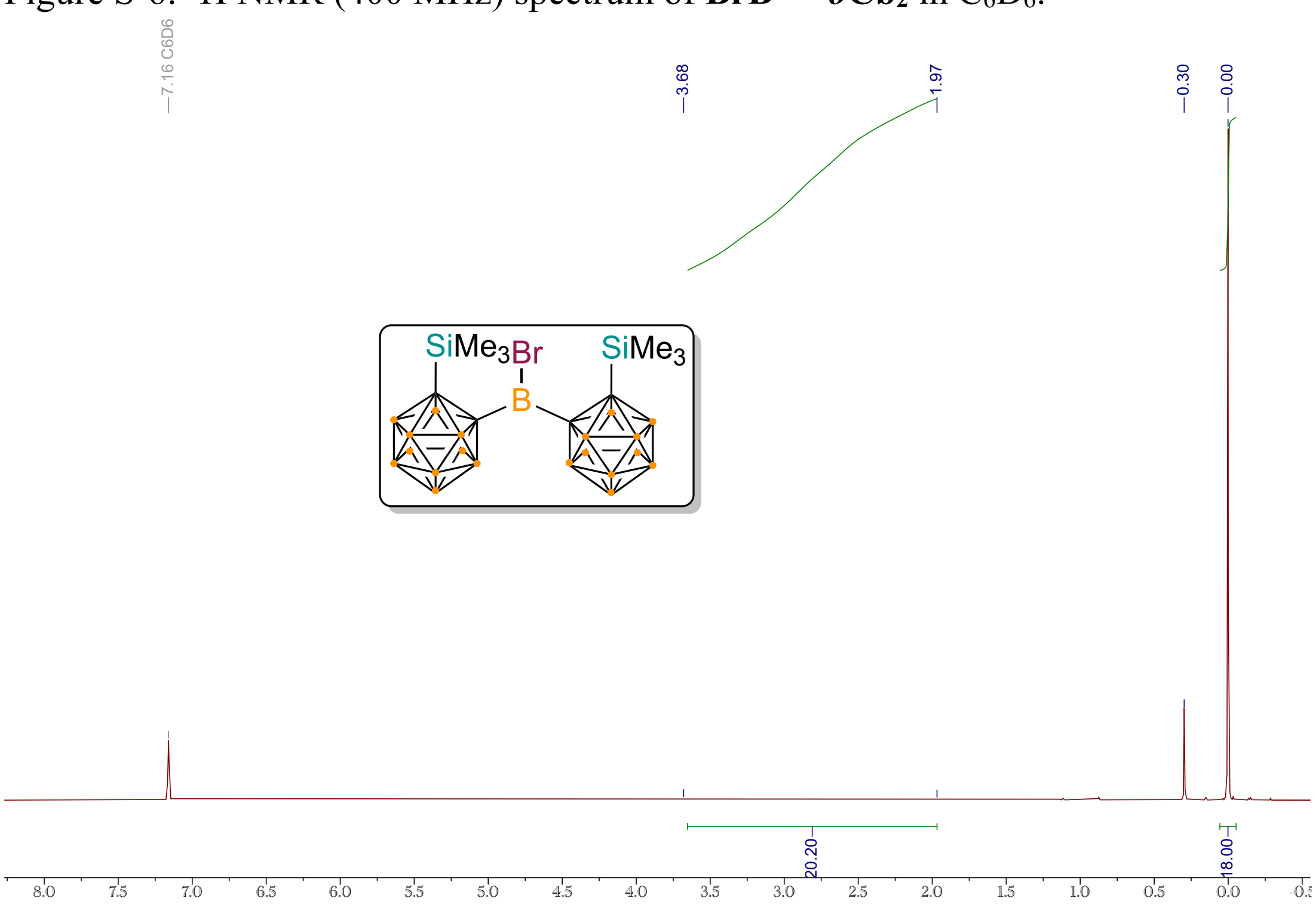

Figure S-7: $^{13}C\{^{1}H\}$ NMR (101 MHz) spectrum of **BrB^{TMS}*o*Cb$_2$** in C_6D_6.

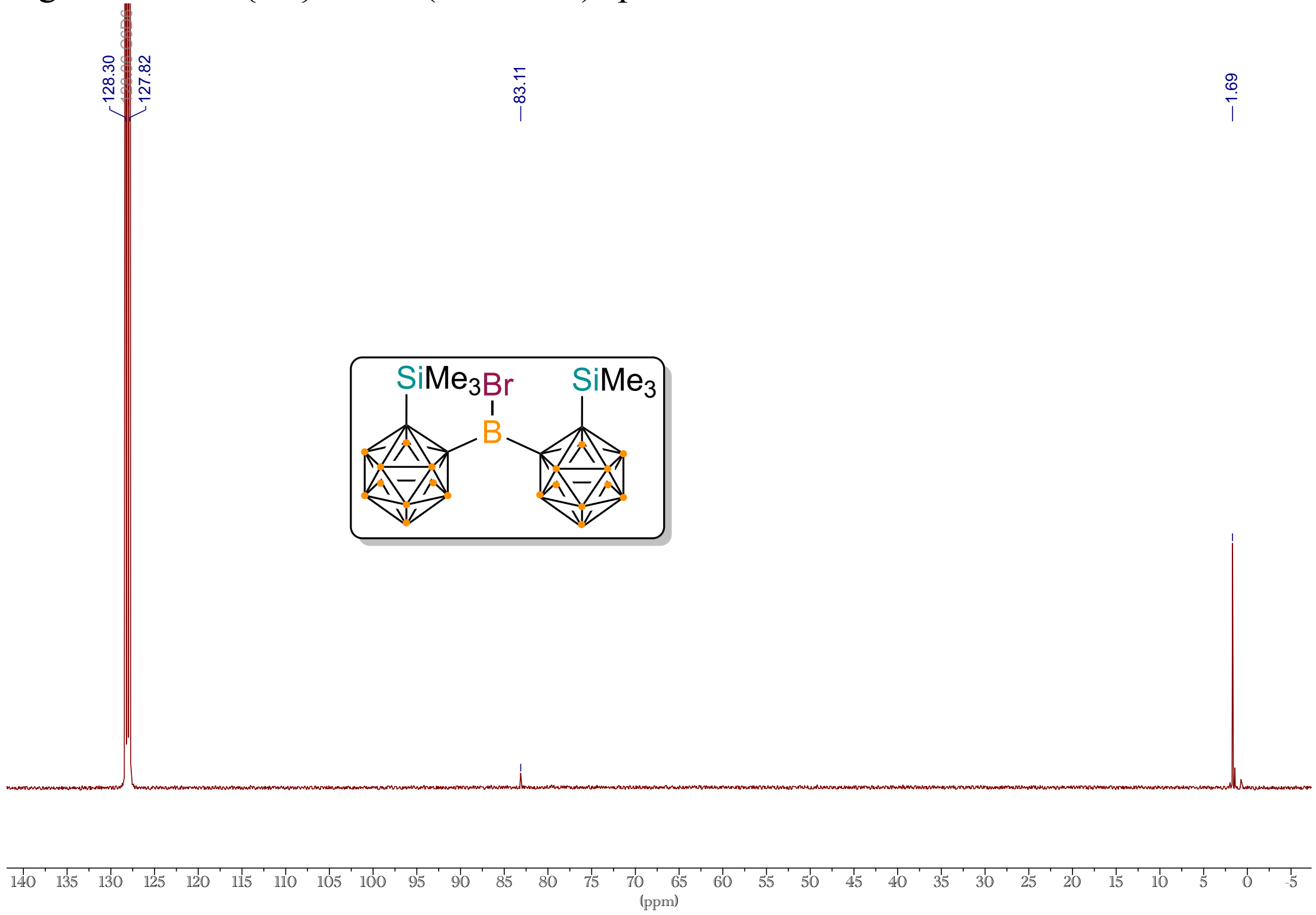

Figure S-8: $^{11}B\{^{1}H)$ NMR (128 MHz) spectrum of **BrB^{TMS}*o*Cb$_2$** in C_6D_6.

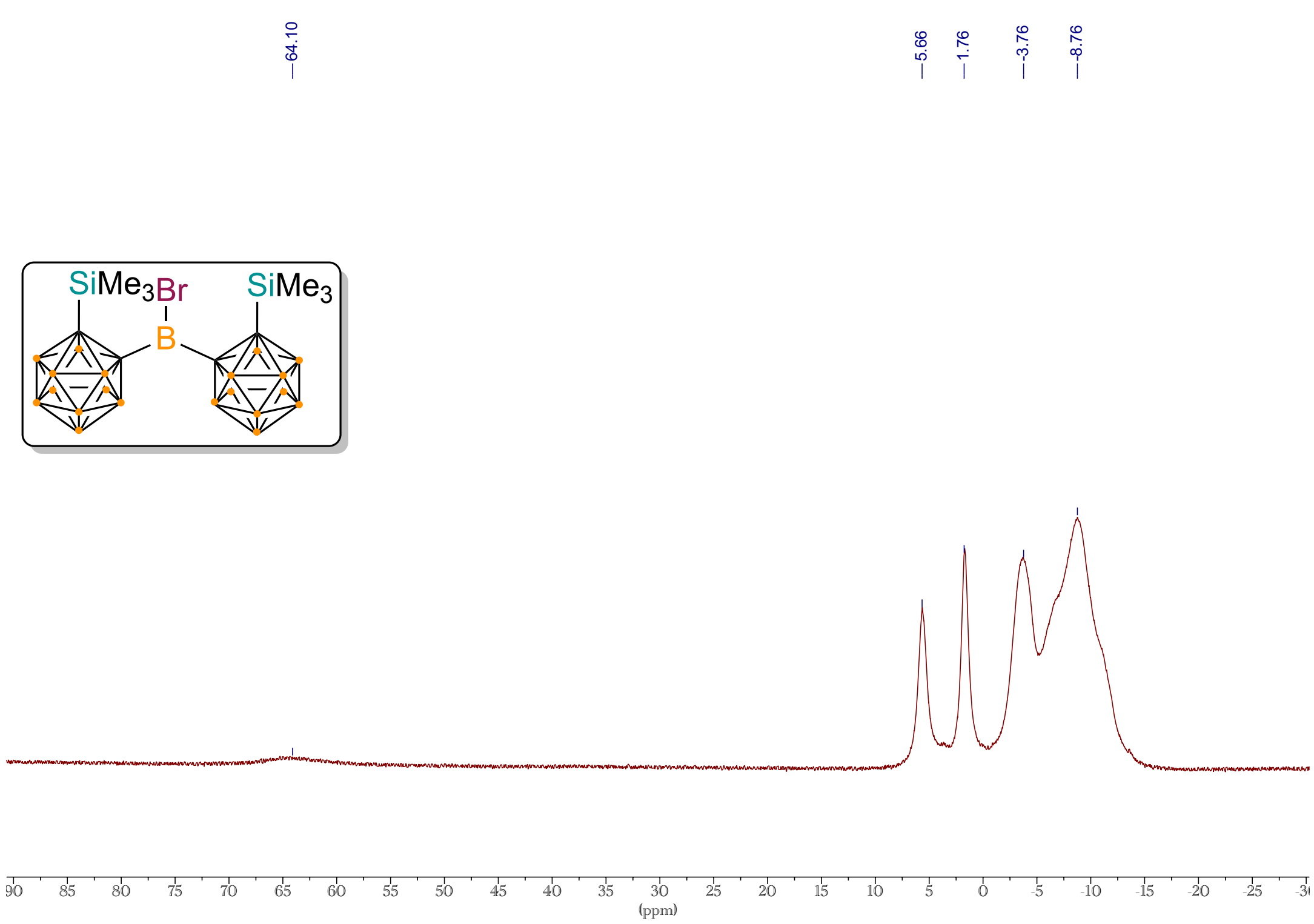

Figure S-9: ^{11}B NMR (128 MHz) spectrum of **BrB$^{\mathbf{TMS}}$*o*Cb$_2$** in C_6D_6.

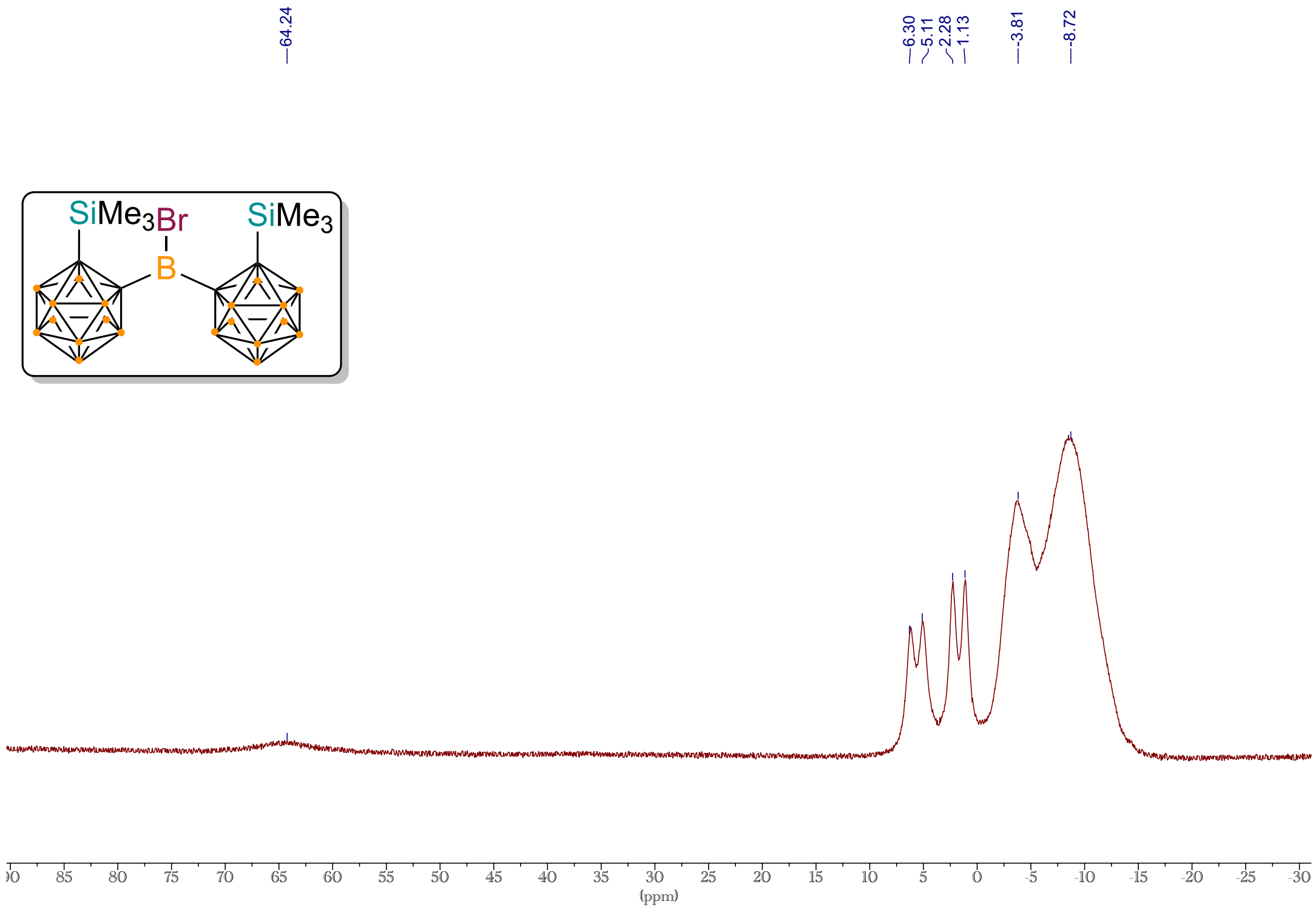

Figure S-10: ^{29}Si{^{1}H} NMR (79 MHz) spectrum of **BrB$^{\mathbf{TMS}}$*o*Cb$_2$** in C_6D_6.

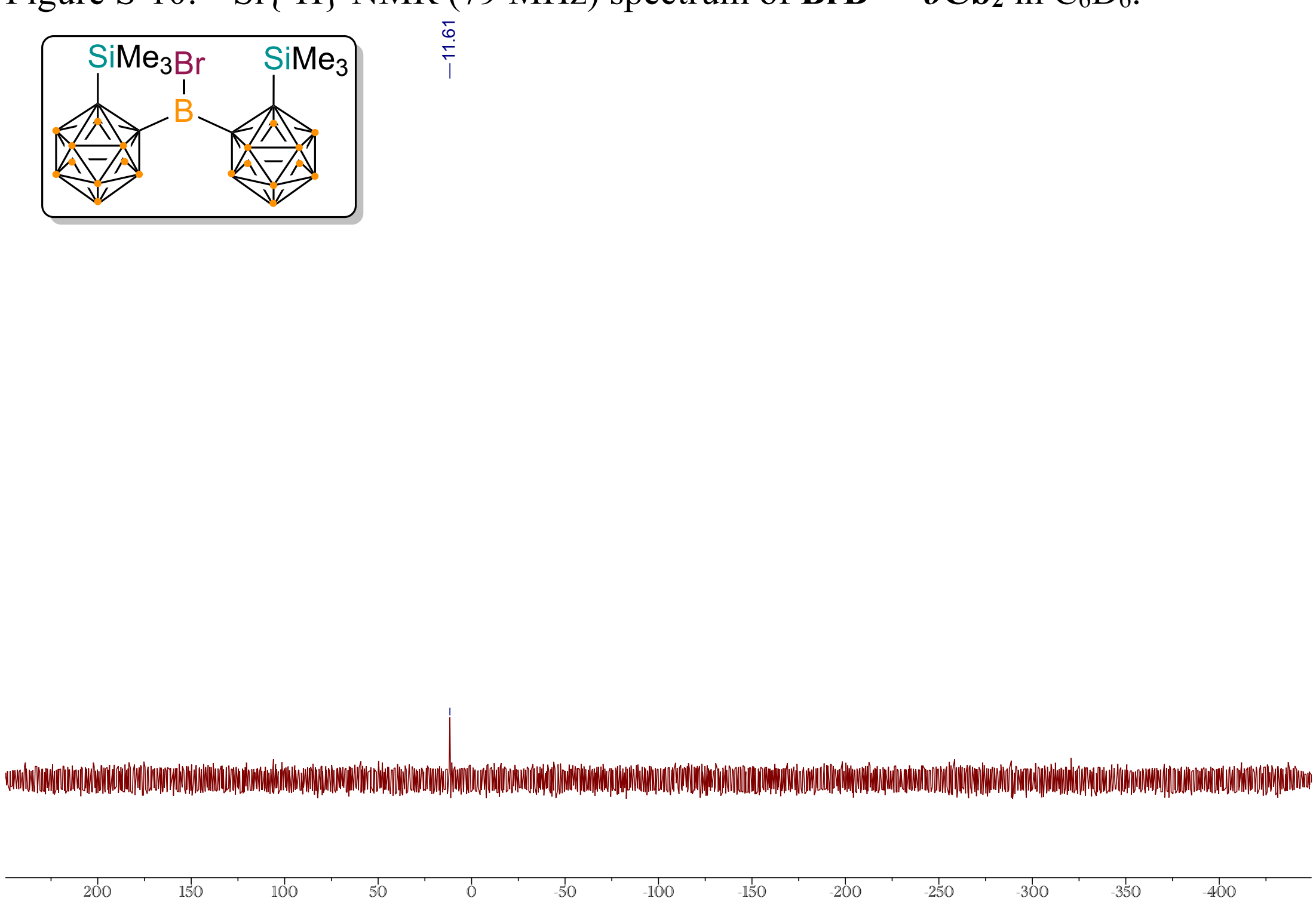

Figure S-11: ^{1}H NMR (600 MHz) spectrum of **K[2]** in C_6D_6 (* toluene).

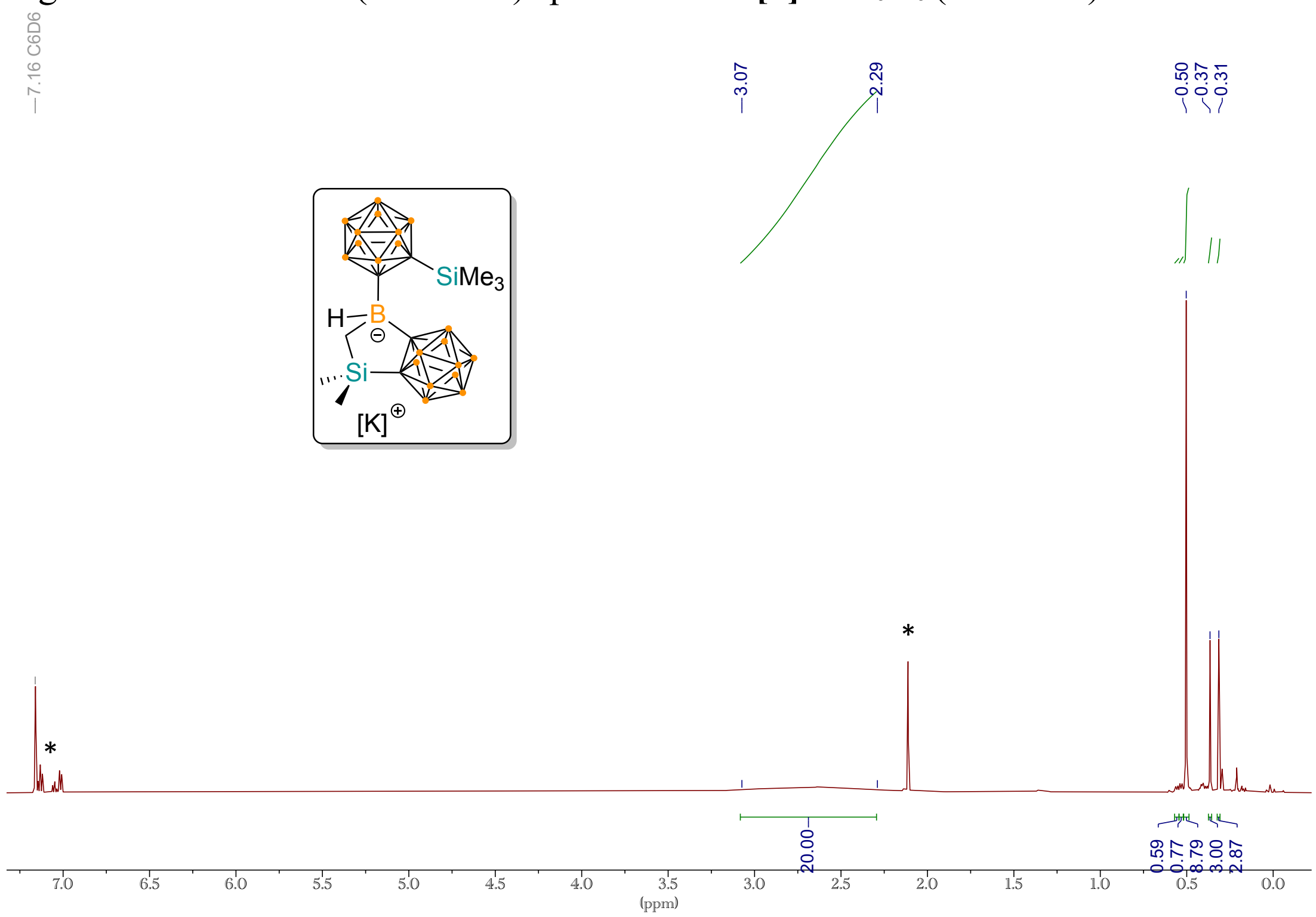

Figure S-12: ^{13}C{^{1}H} NMR (151 MHz) spectrum of **K[2]** in C_6D_6 (* toluene).

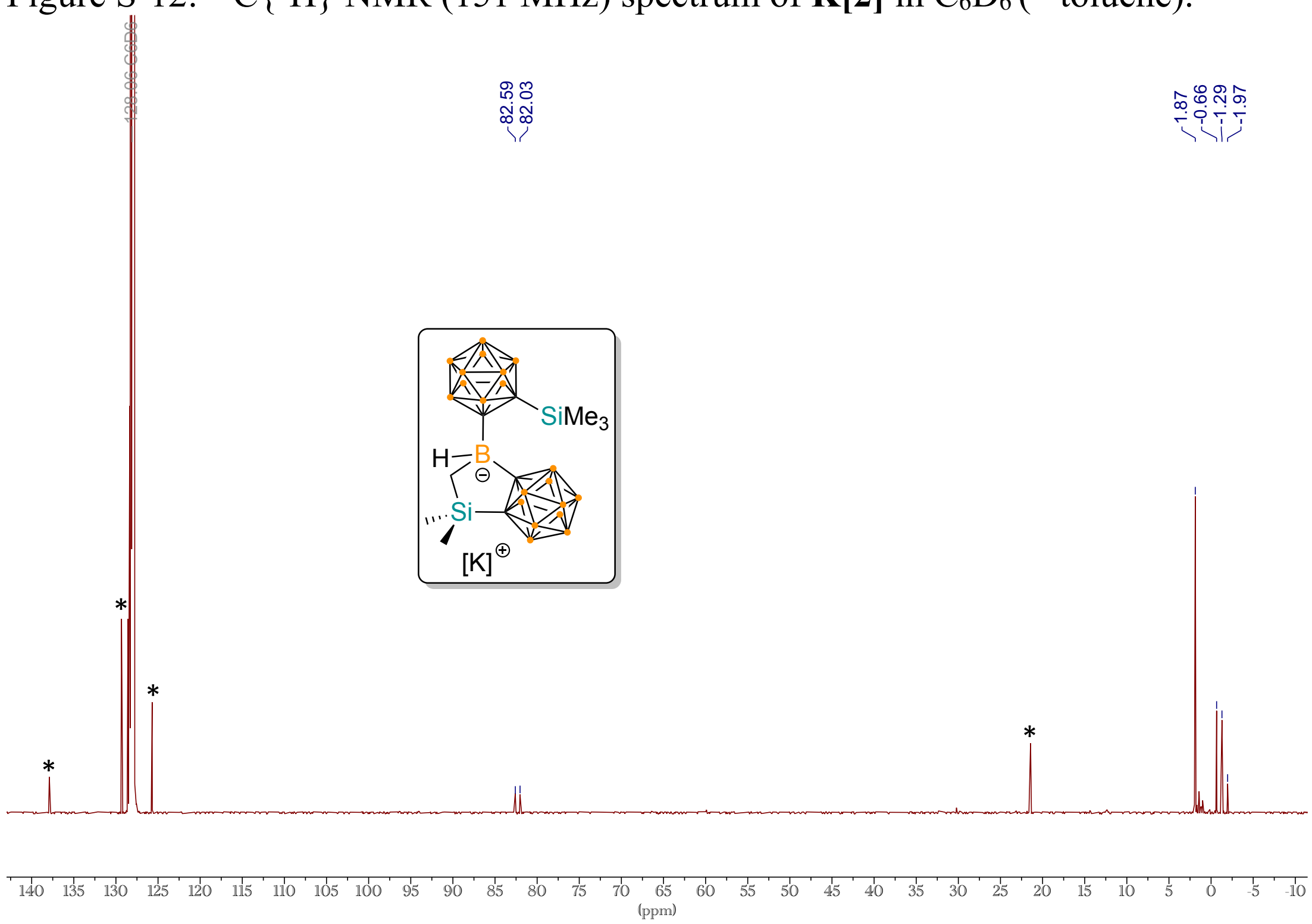

Figure S-13: $^{11}B\{^{1}H)$ NMR (193 MHz) spectrum of **K[2]** in C_6D_6.

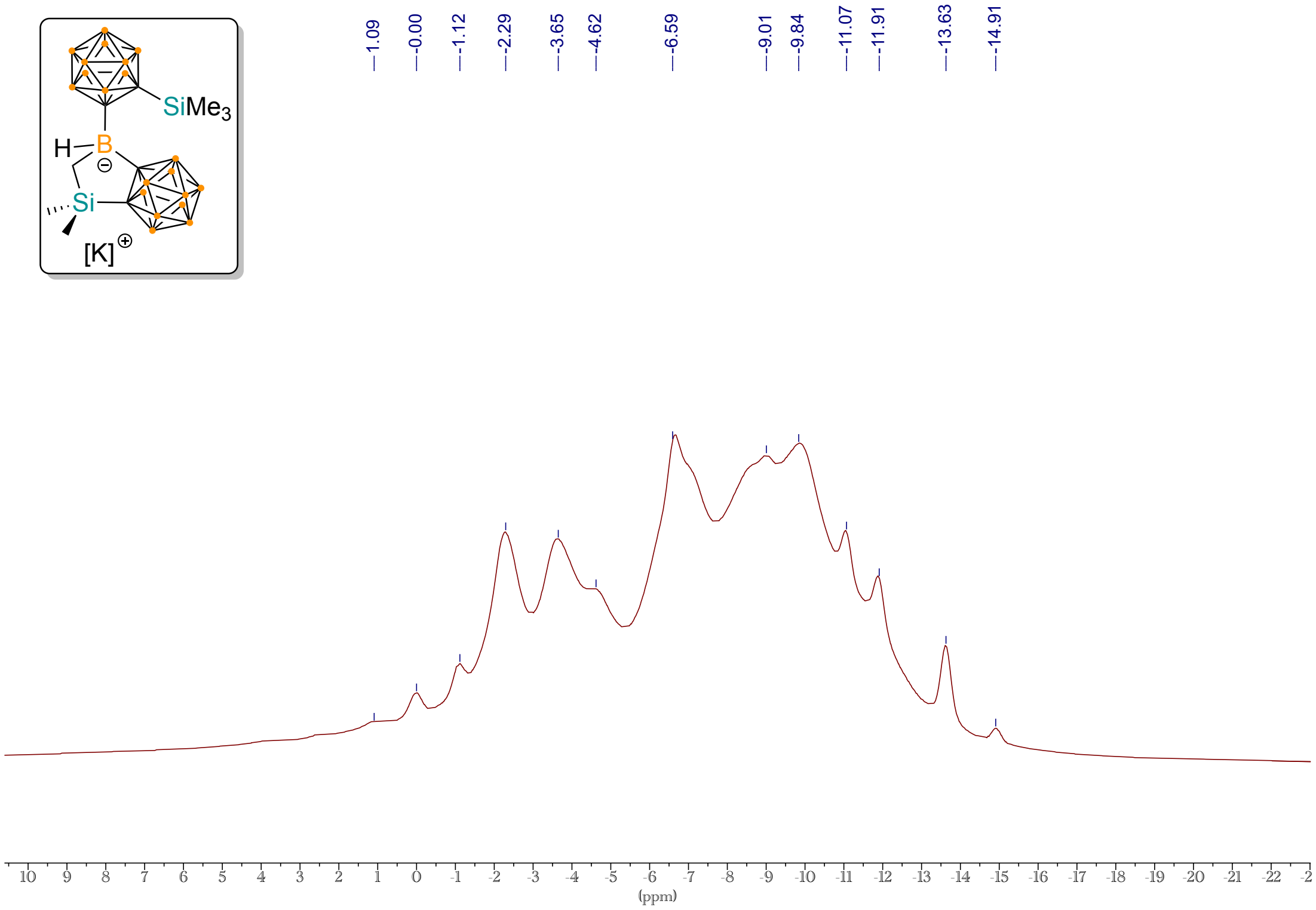

Figure S-14: ^{11}B NMR (193 MHz) spectrum of **K[2]** in C_6D_6.

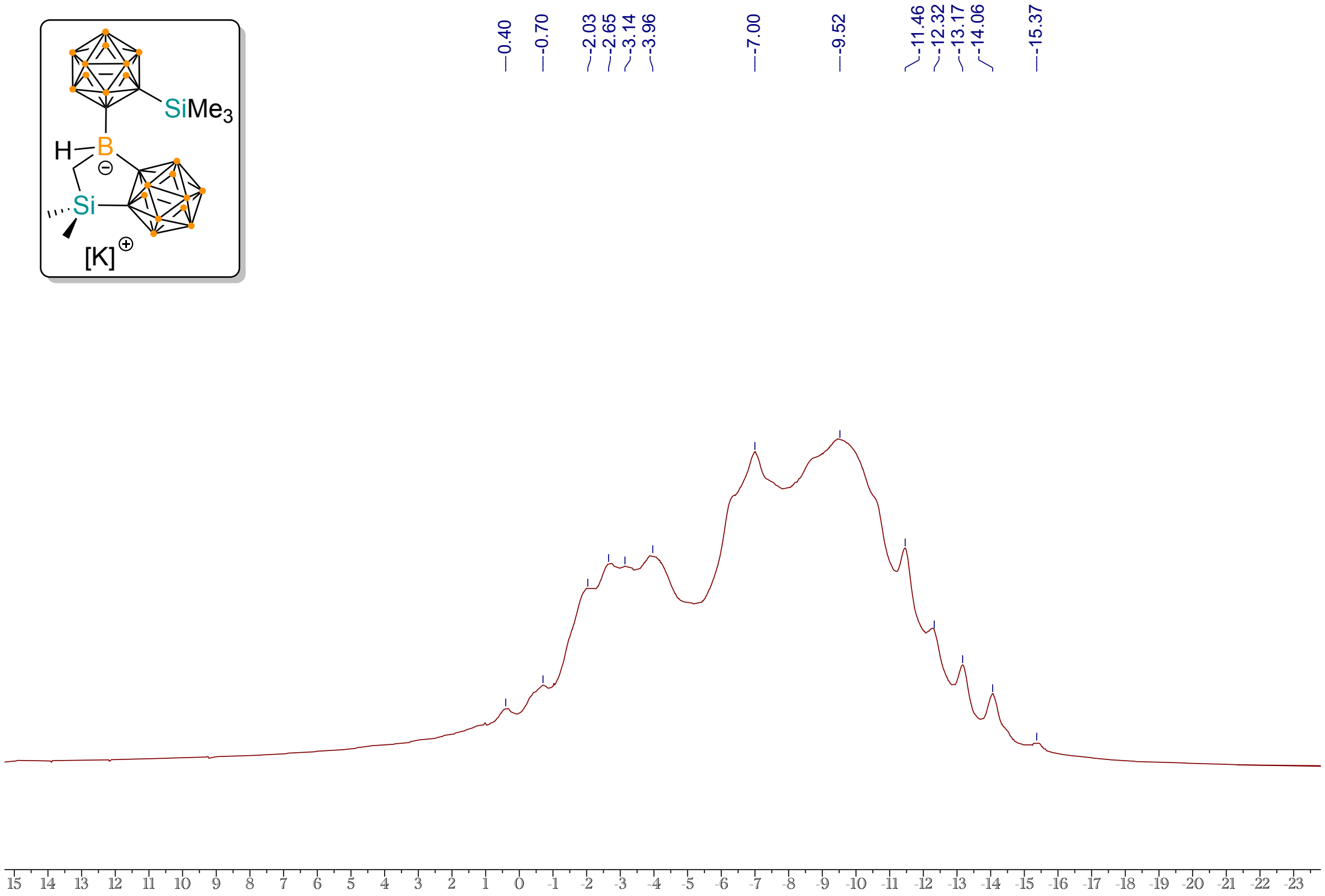

Figure S-15: $^{29}Si\{^{1}H\}$ NMR (119 MHz) spectrum of **K[2]** in C_6D_6.

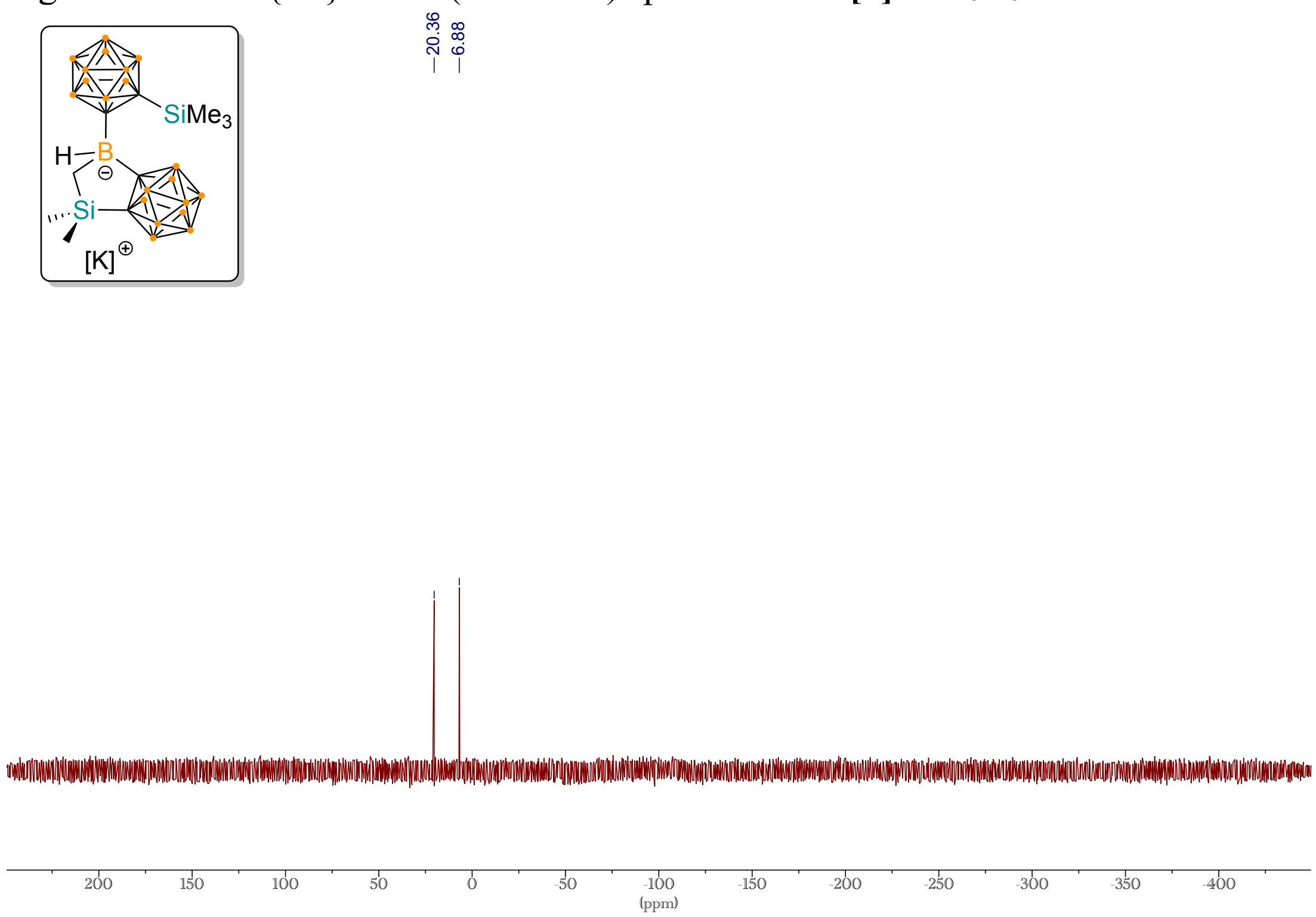

Figure S-16: ^{1}H NMR (600 MHz) spectrum of **K-crown[2]** in C_6D_6 (* toluene).

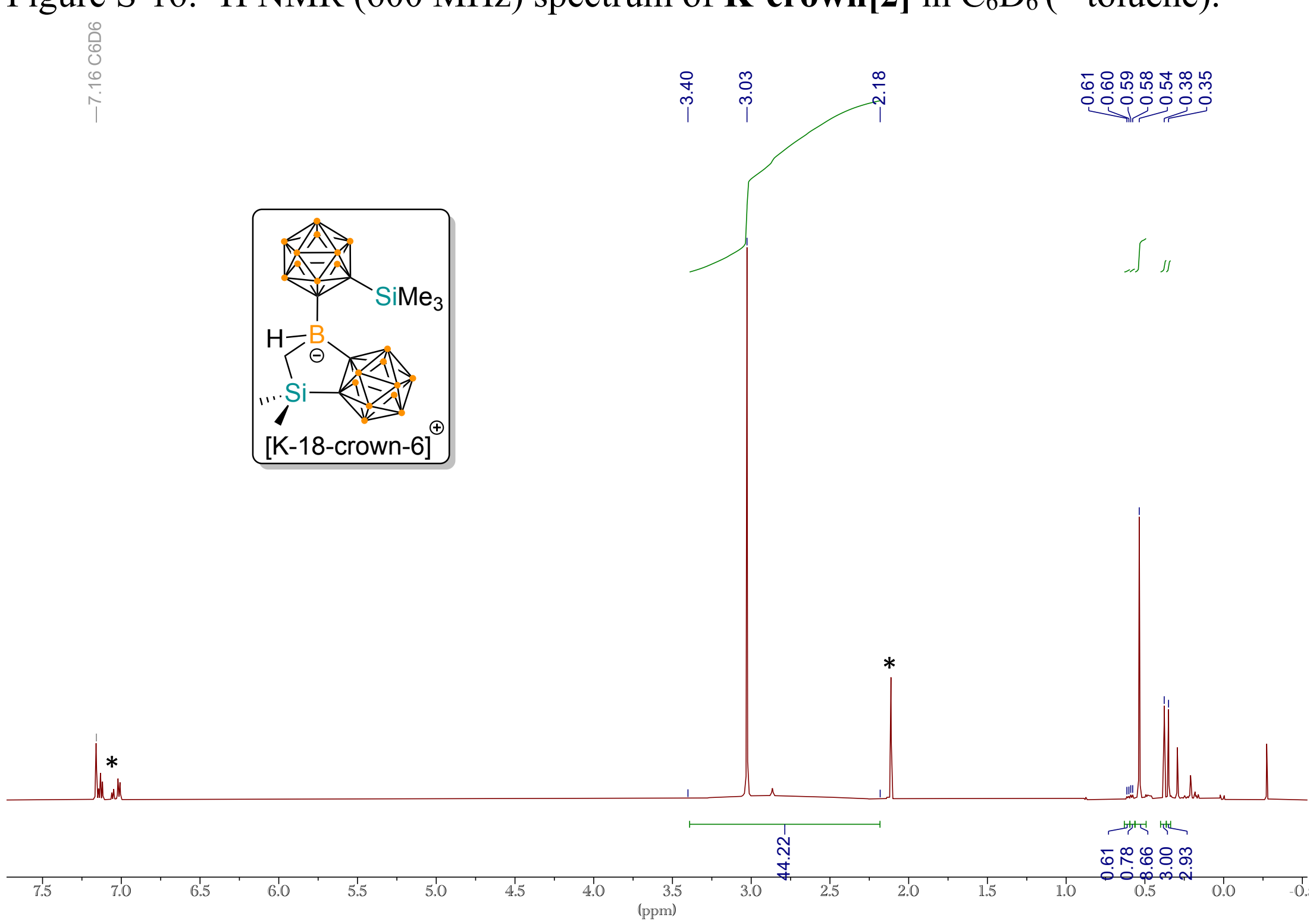

Figure S-17: $^{13}C\{^{1}H\}$ NMR (151 MHz) spectrum of **K-crown[2]** in C_6D_6 (* toluene).

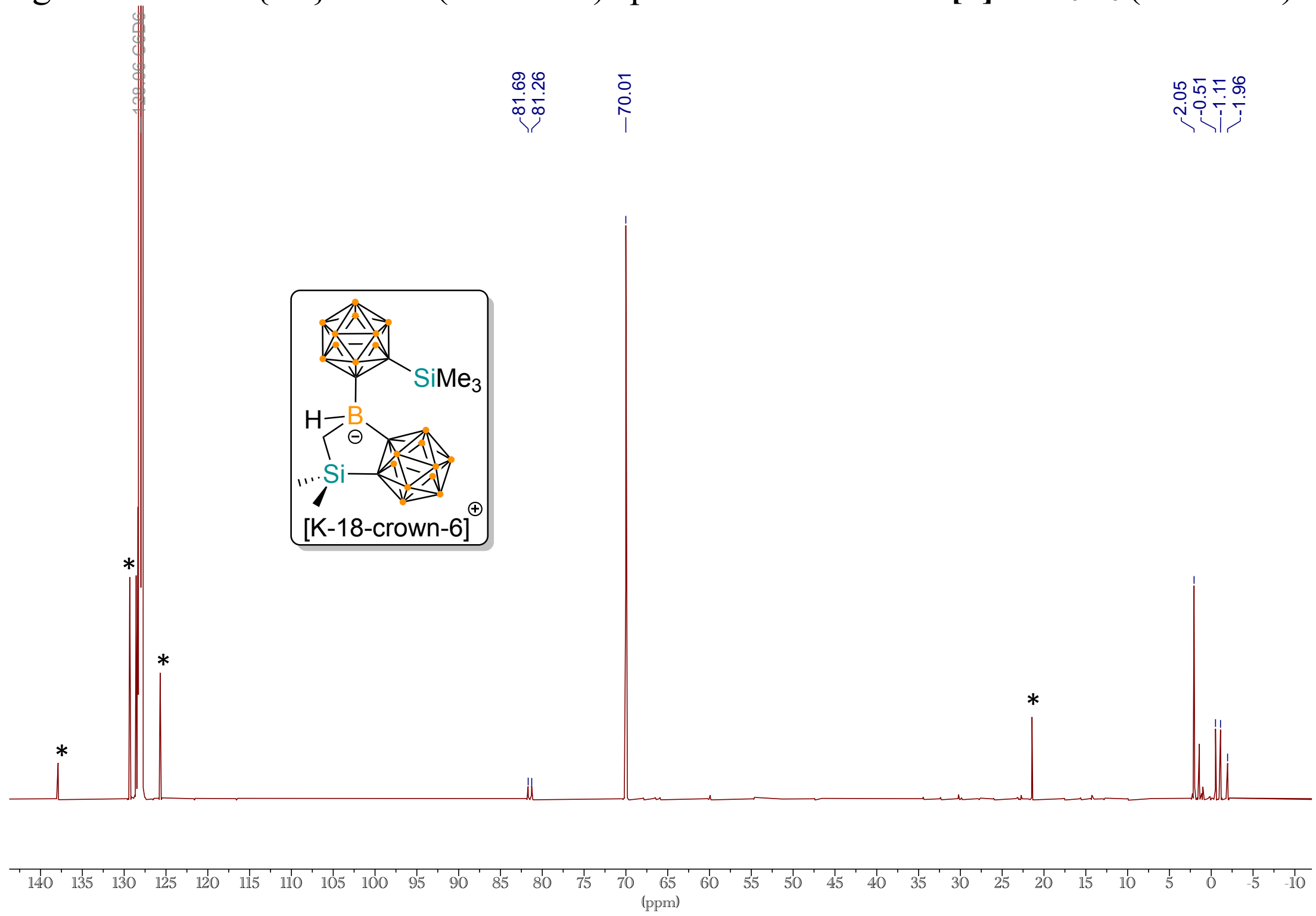

Figure S-18: $^{11}B\{^{1}H\}$ NMR (193 MHz) spectrum of **K-crown[2]** in C_6D_6.

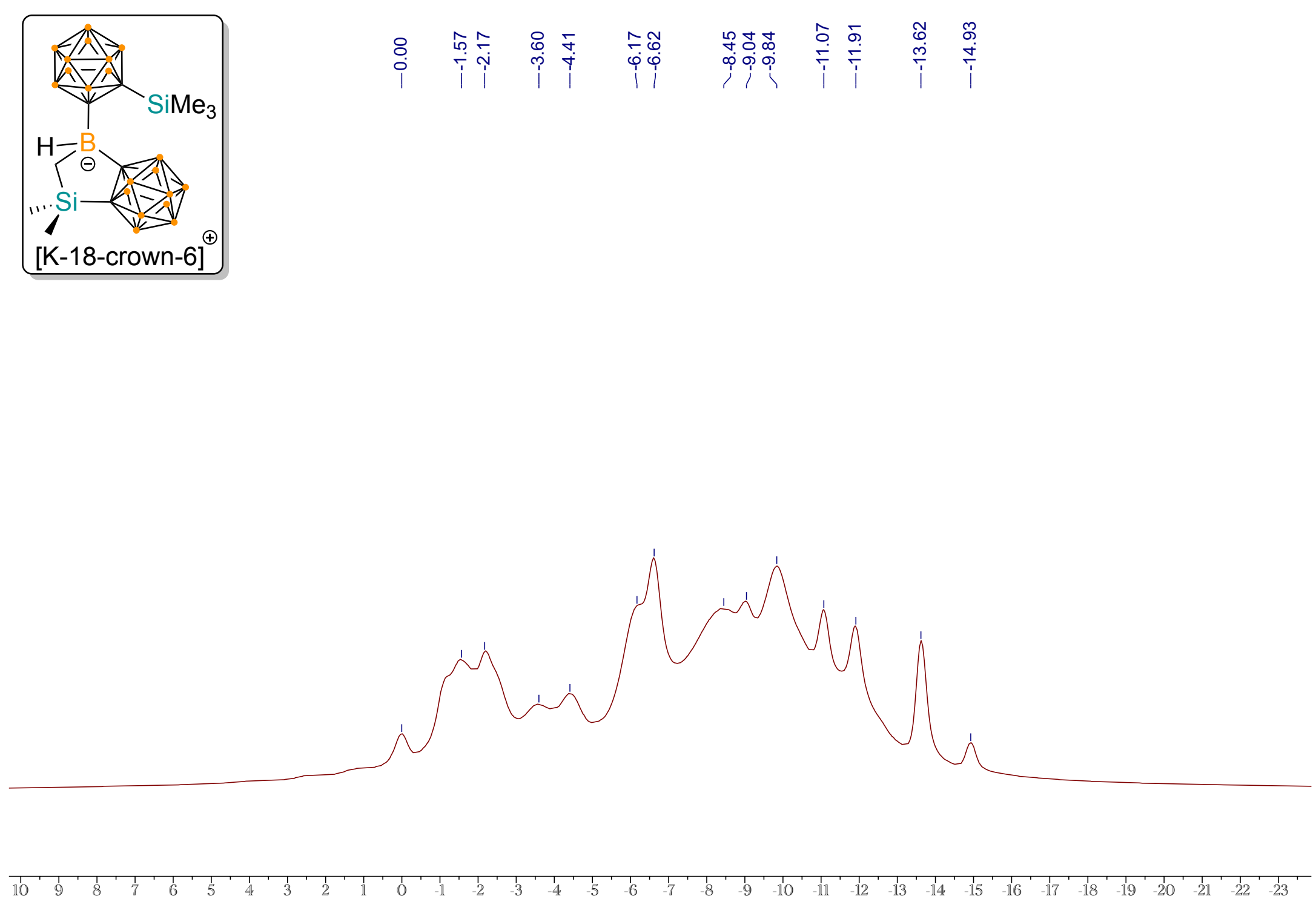

Figure S-19: ^{11}B NMR (193 MHz) spectrum of **K-crown[2]** in C_6D_6.

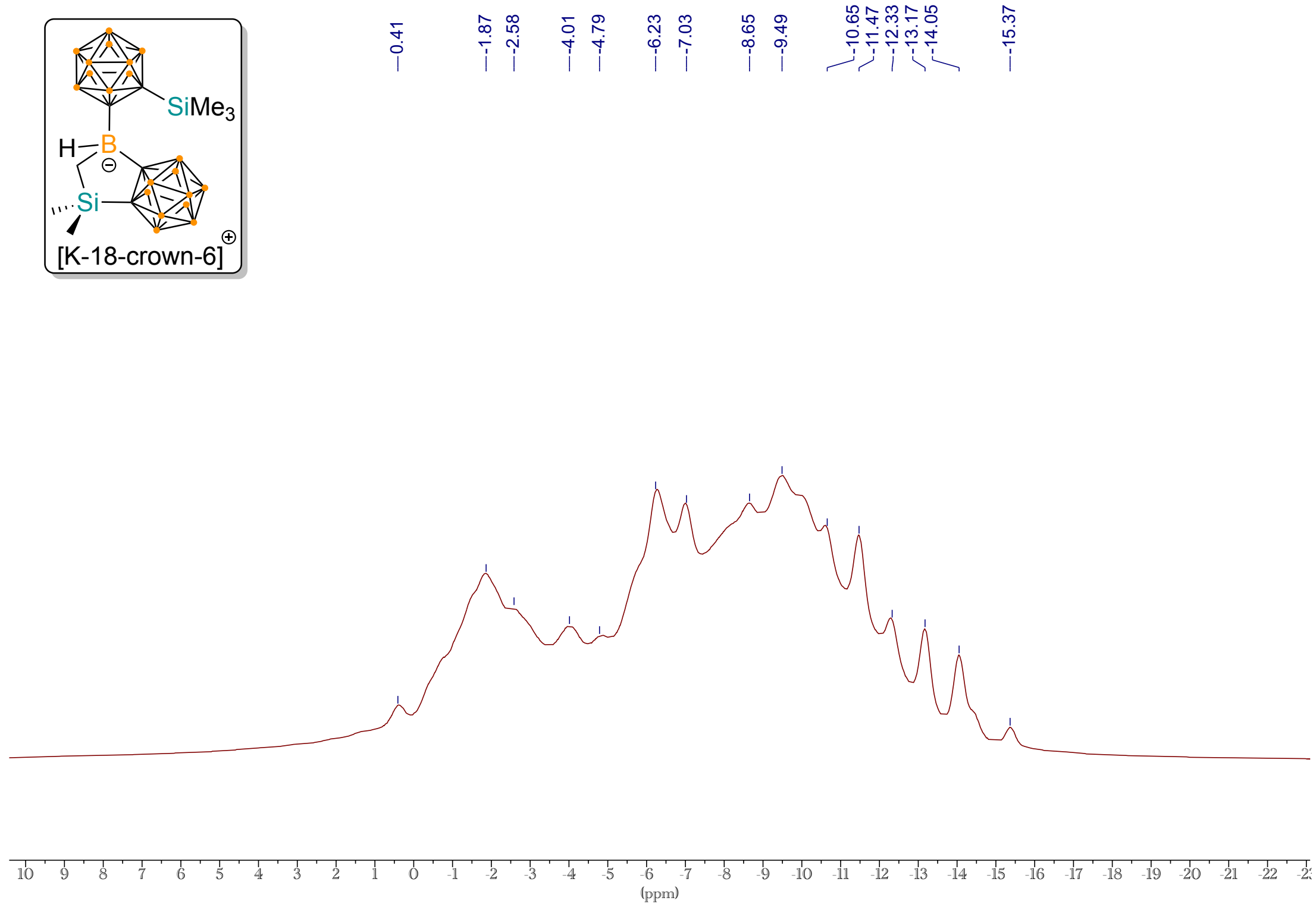

Figure S-20: $^{29}Si\{^1H\}$ NMR (119 MHz) spectrum of **K-crown[2]** in C_6D_6.

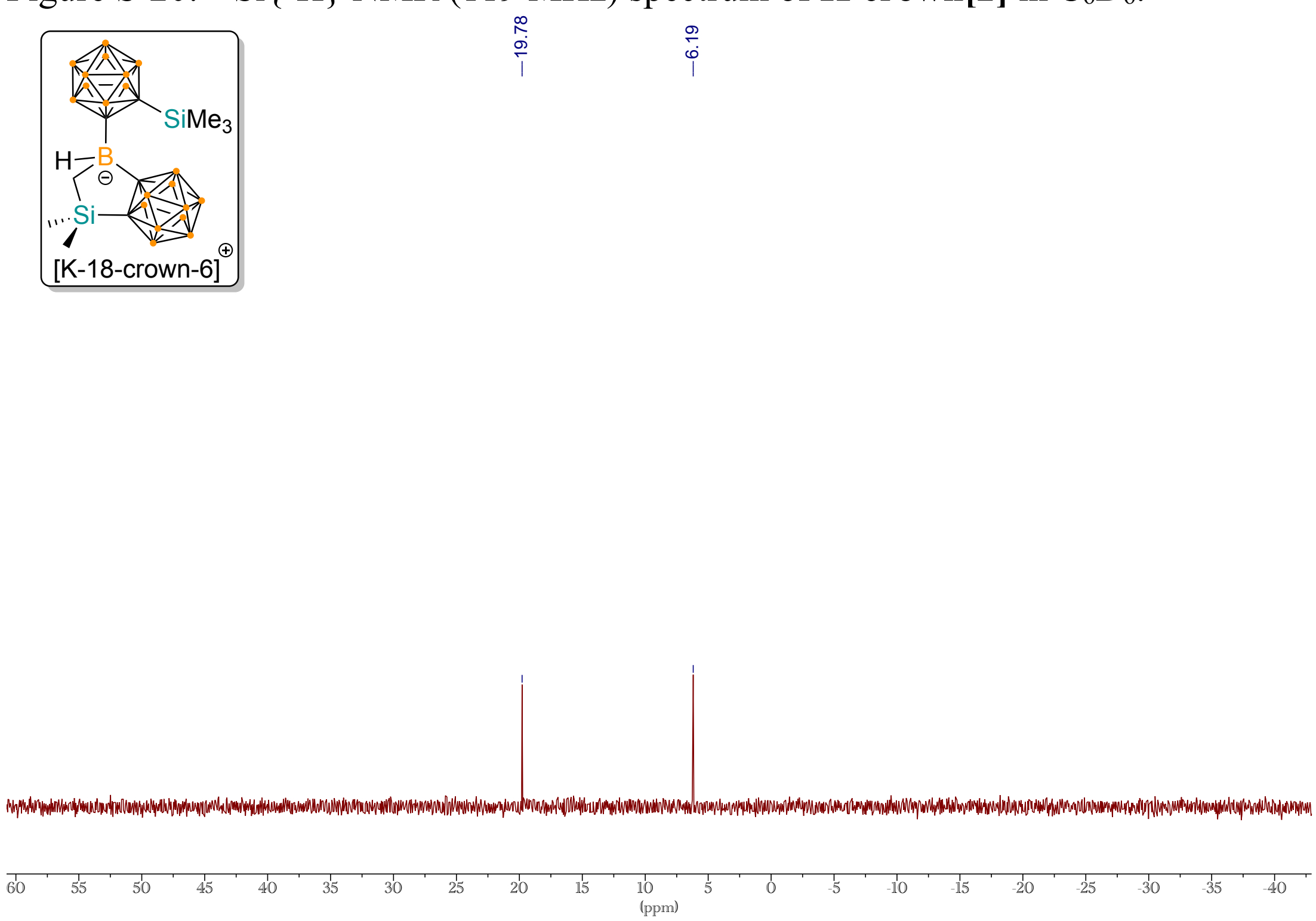

Figure S-21: ^{1}H NMR (400 MHz) spectrum of **3** in C_6D_6.

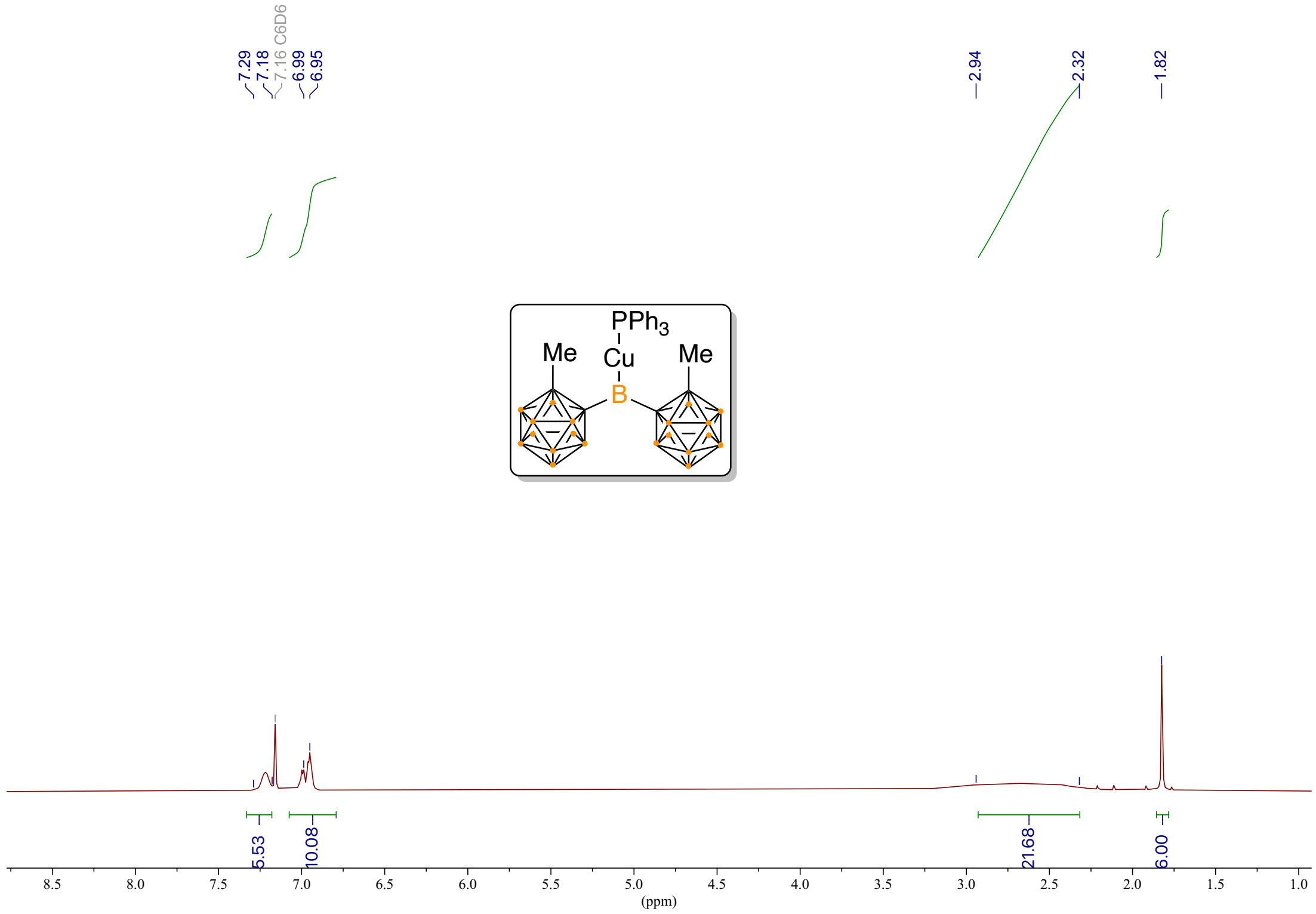

Figure S-22: $^{13}C\{^{1}H\}$ NMR (101 MHz) spectrum of **3** in C_6D_6.

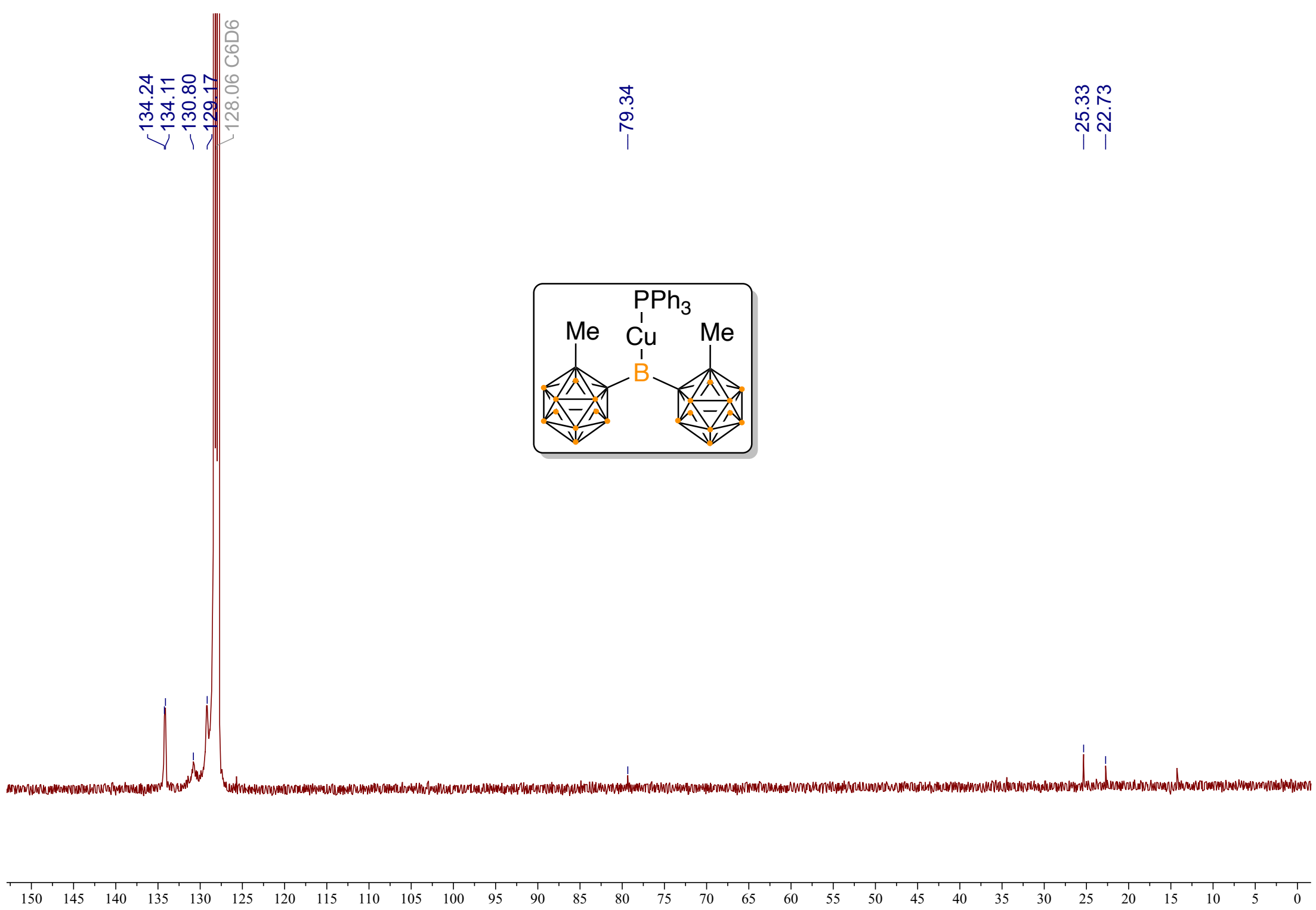

Figure S-23: $^{11}B\{^{1}H\}$ NMR (128 MHz) spectrum of **3** in C_6D_6.

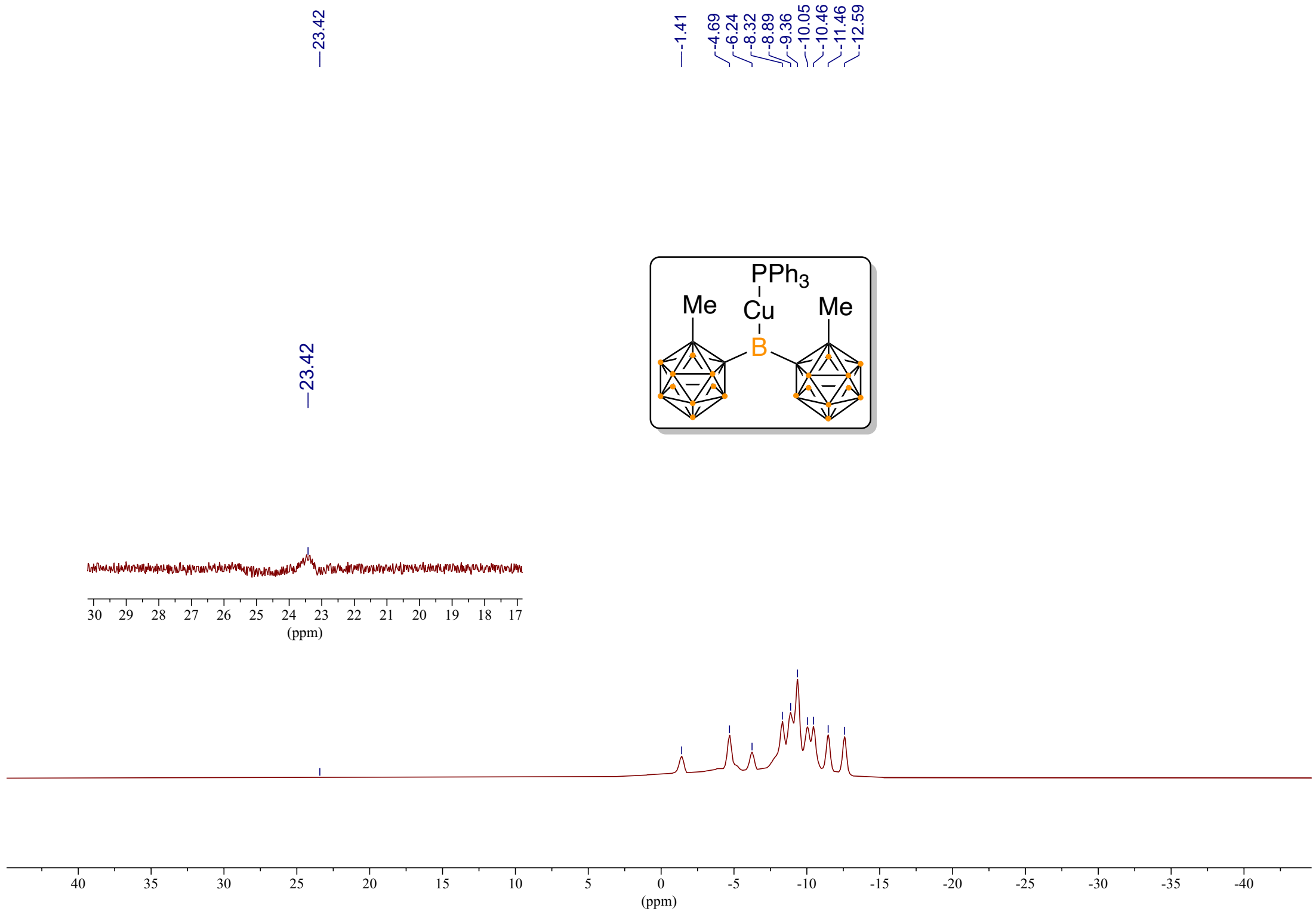

Figure S-24: ^{11}B NMR (128 MHz) spectrum of **3** in C_6D_6.

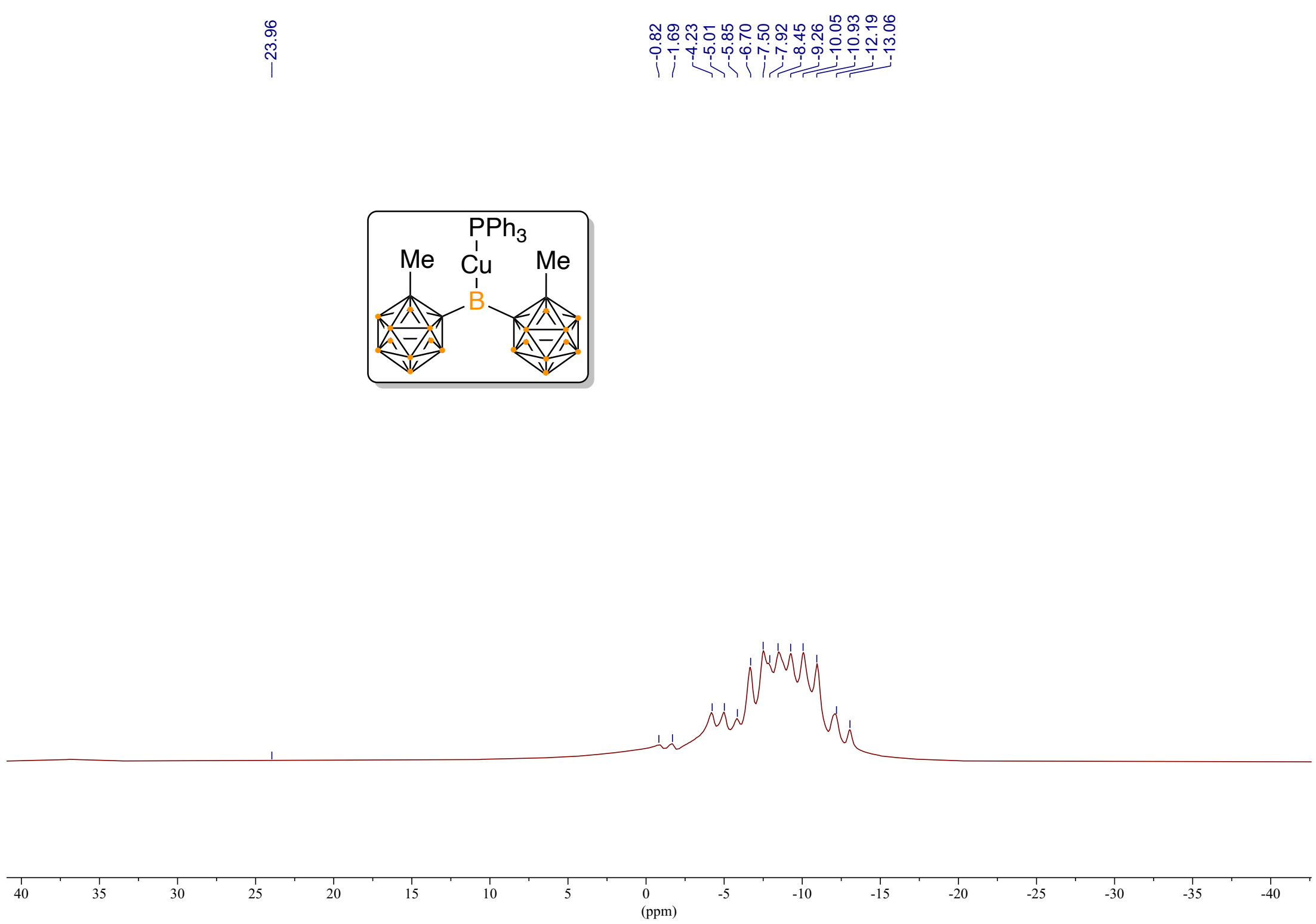

Figure S-25: $^{31}P\{^{1}H\}$ NMR (162 MHz) spectrum of **3** in C_6D_6.

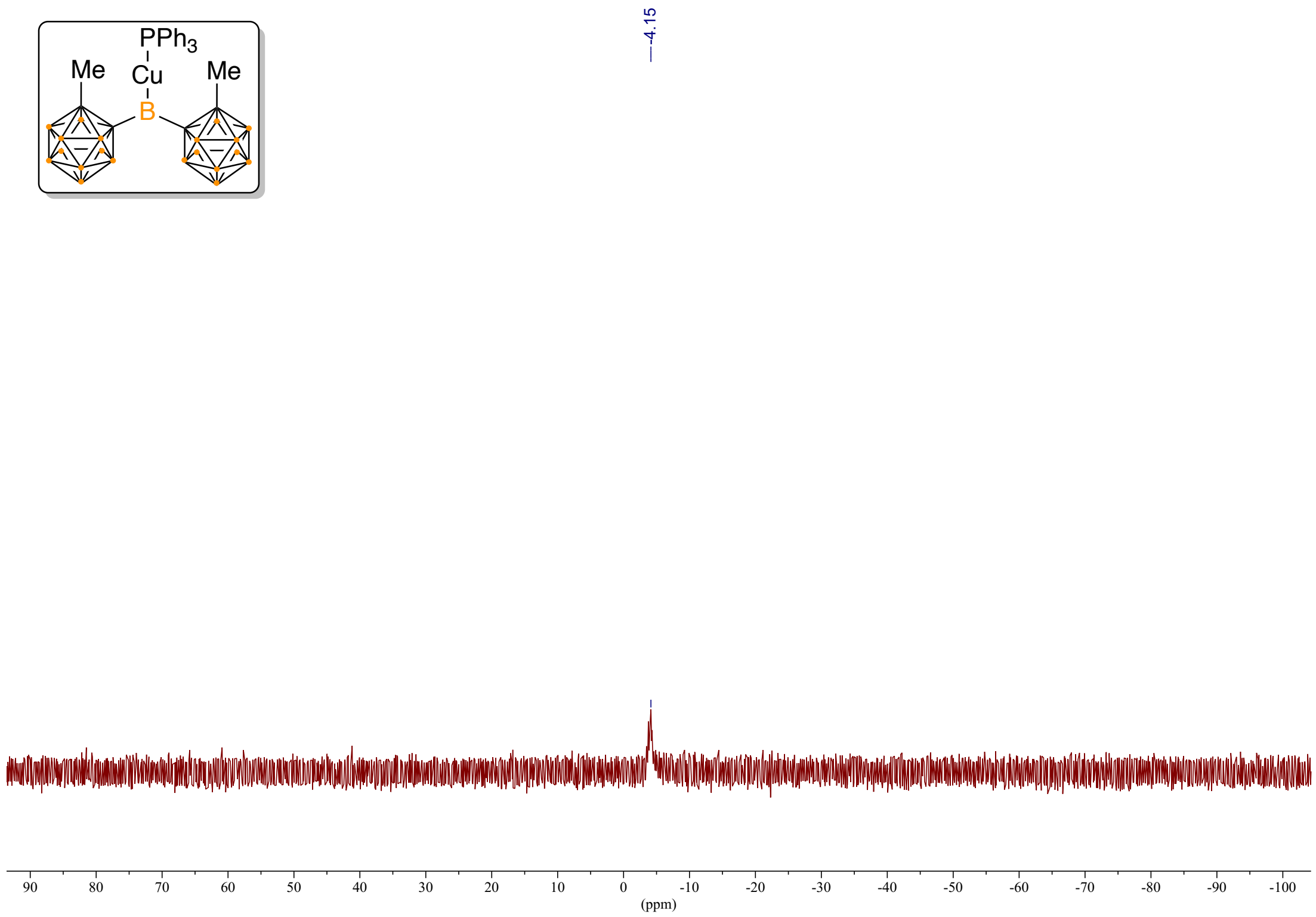

Figure S-26: ^{1}H NMR (600 MHz) spectrum of **4** in $CDCl_3$.

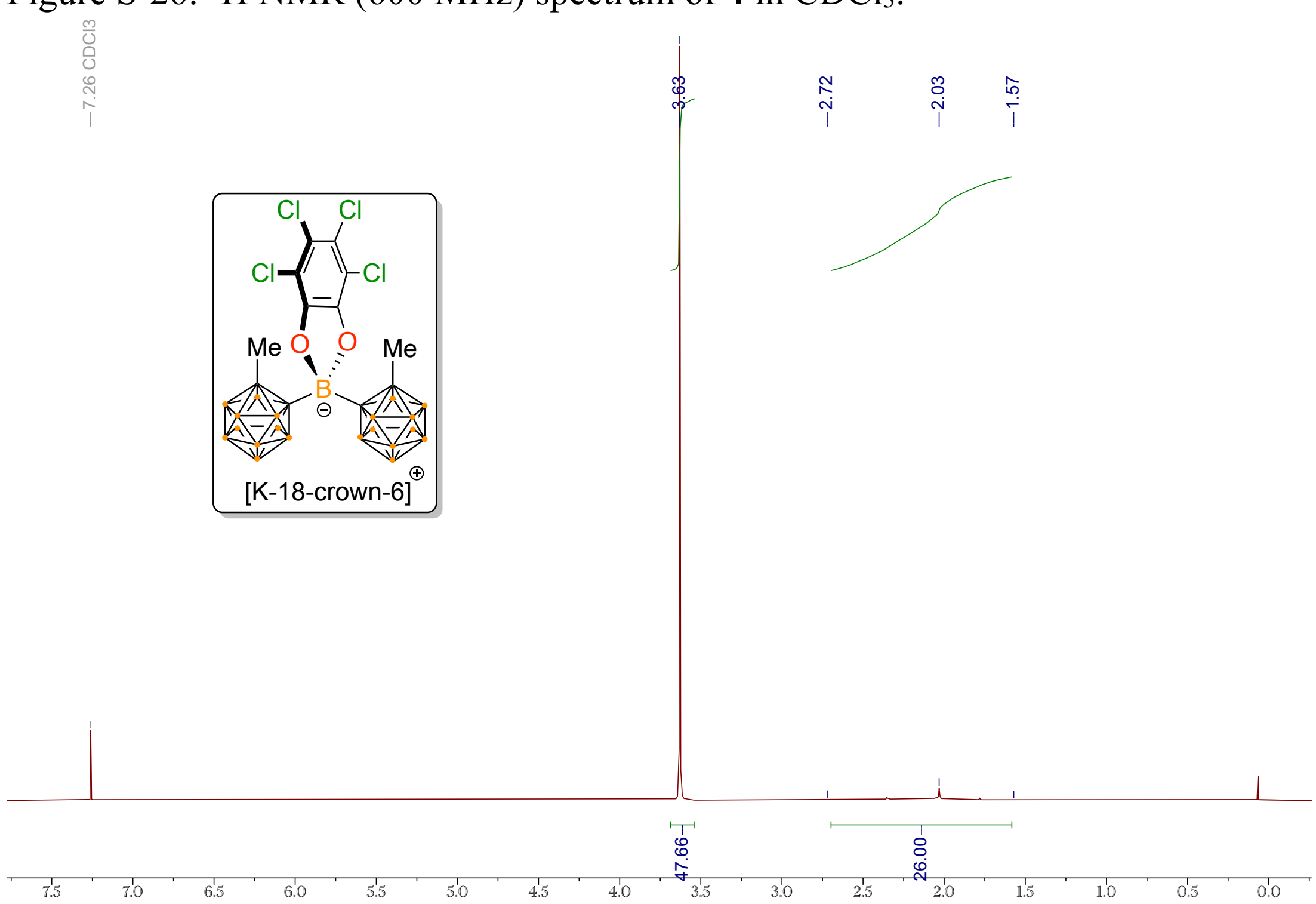

Figure S-27: $^{13}C\{^{1}H\}$ NMR (151 MHz) spectrum of **4** in $CDCl_3$.

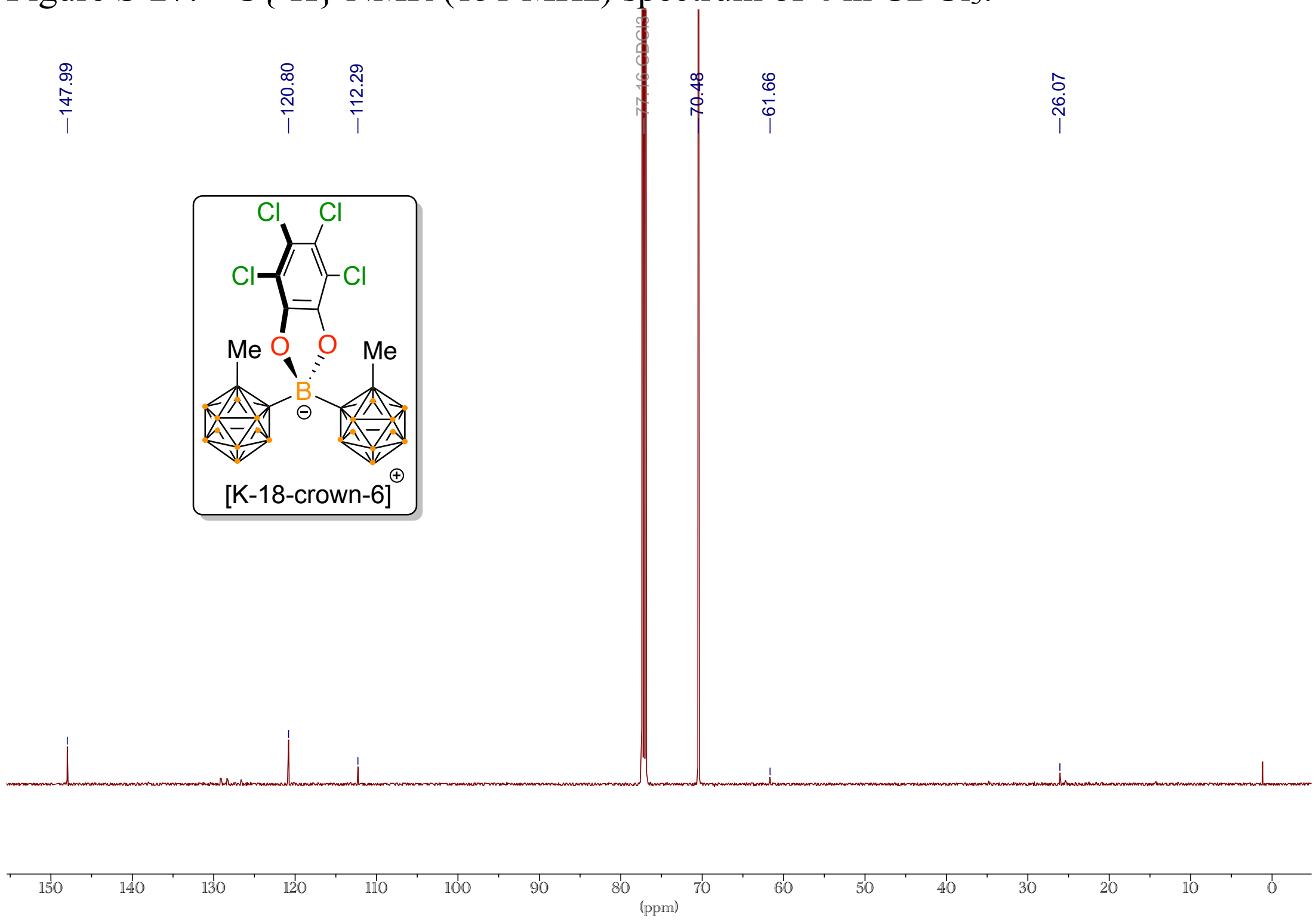

Figure S-28: $^{11}B\{^{1}H\}$ NMR (193 MHz) spectrum of **4** in $CDCl_3$.

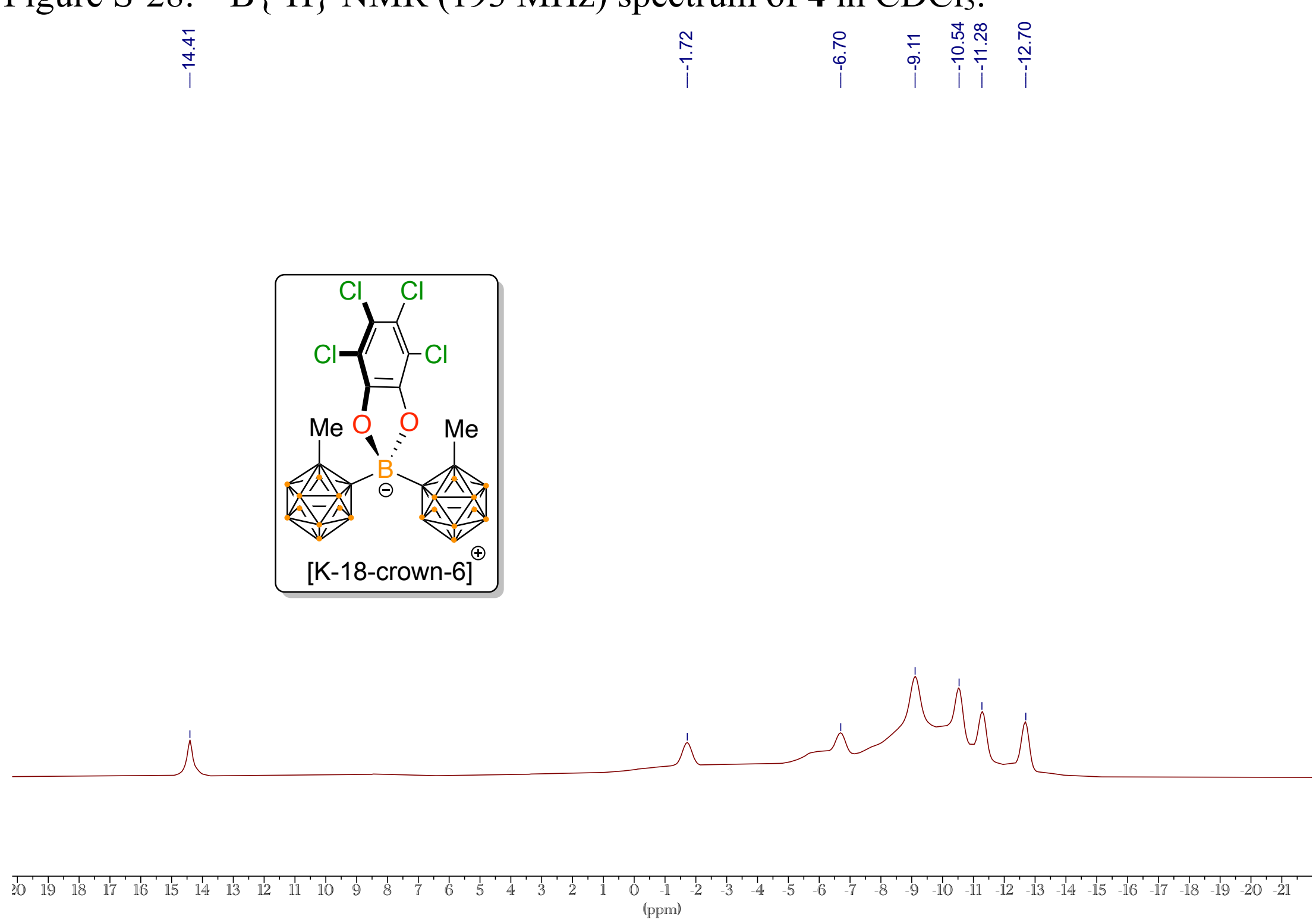

Figure S-29: ^{11}B NMR (193 MHz) spectrum of **4** in $CDCl_3$.

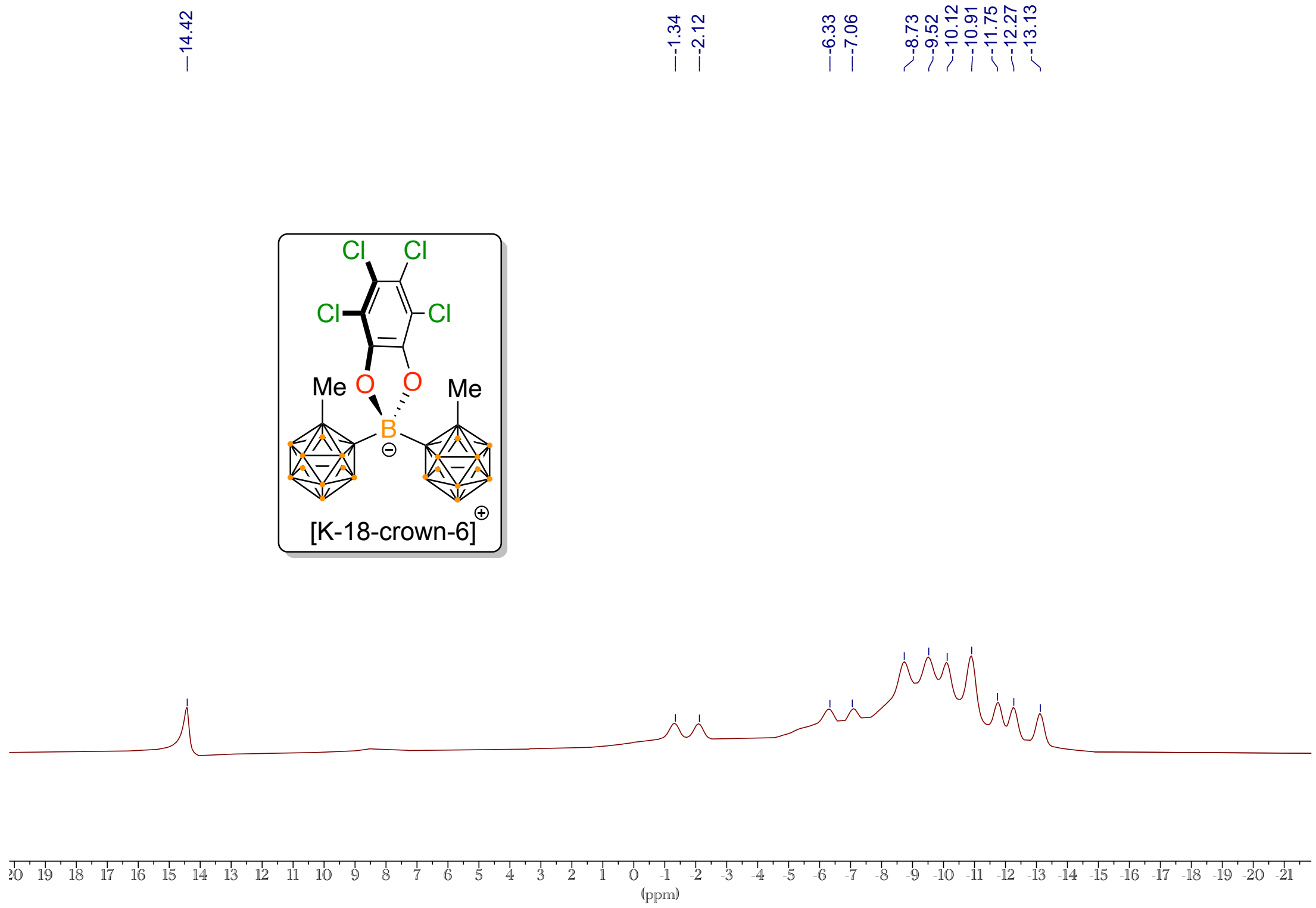

Figure S-30: ^{11}B{^{1}H} NMR (128 MHz) spectrum of **[K][H$_2$B^{Me}*o*Cb$_2$]** in C_6D_6.

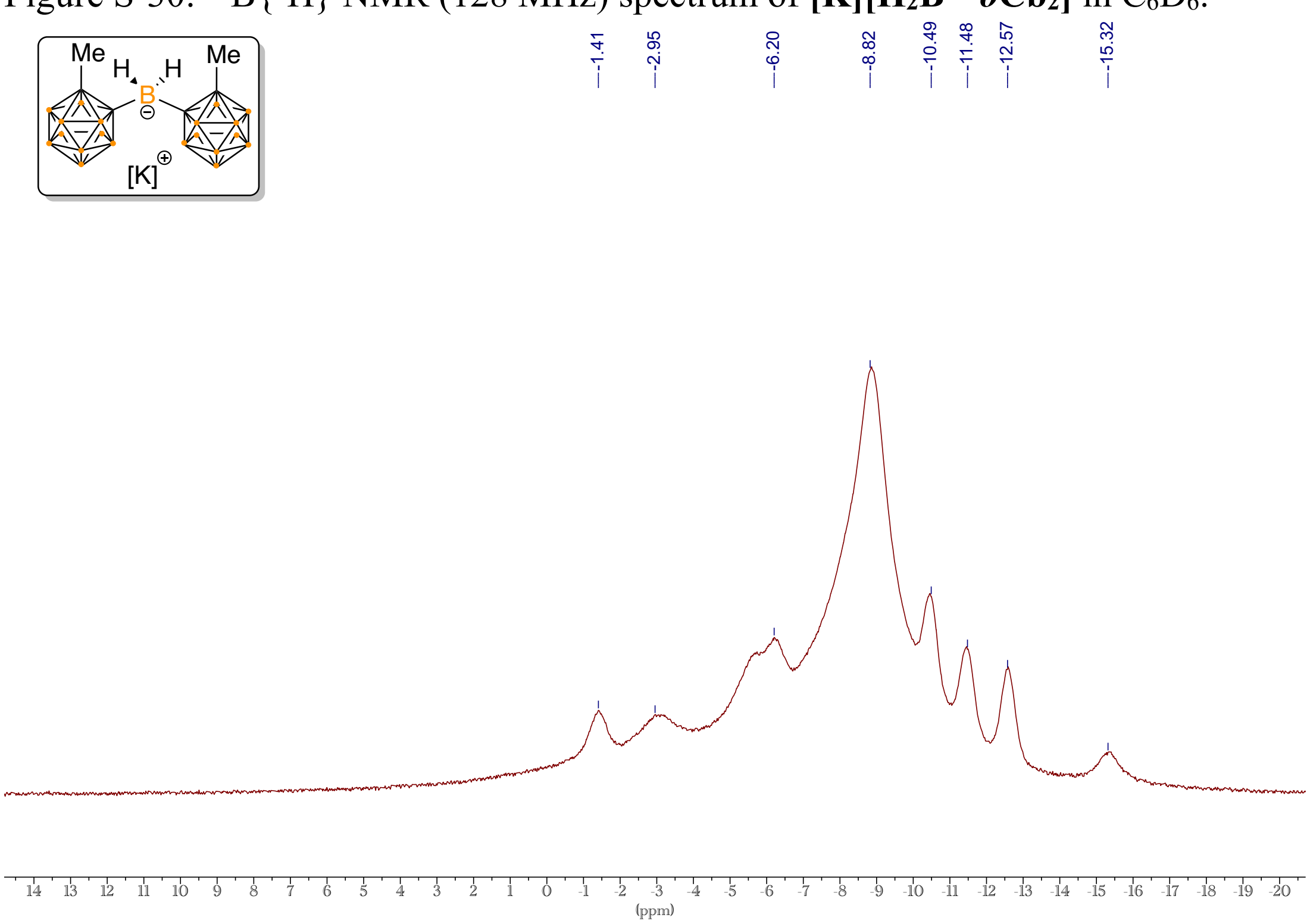

Figure S-31: ^{11}B NMR (128 MHz) spectrum of **[K][H$_2$B^{Me}*o*Cb$_2$]** in C_6D_6.

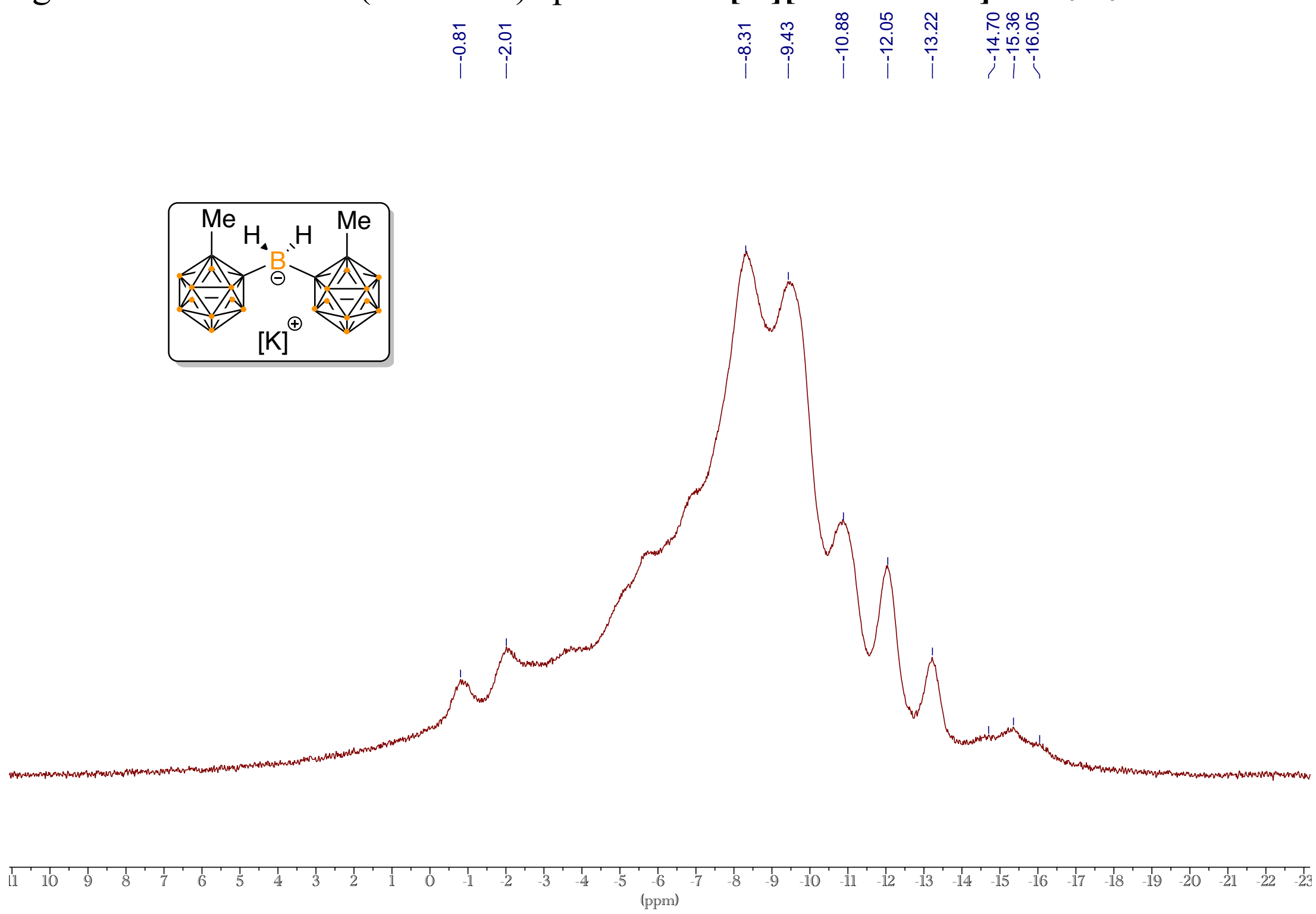

Figure S-32: ^{11}B{^{1}H} NMR (128 MHz) spectrum of the crude reaction mixture of BrB^{Ph}*o*Cb$_2$ and 2.5 eq. of K metal in toluene. Doped with drops of C_6D_6 to enable the spectrometer to lock.

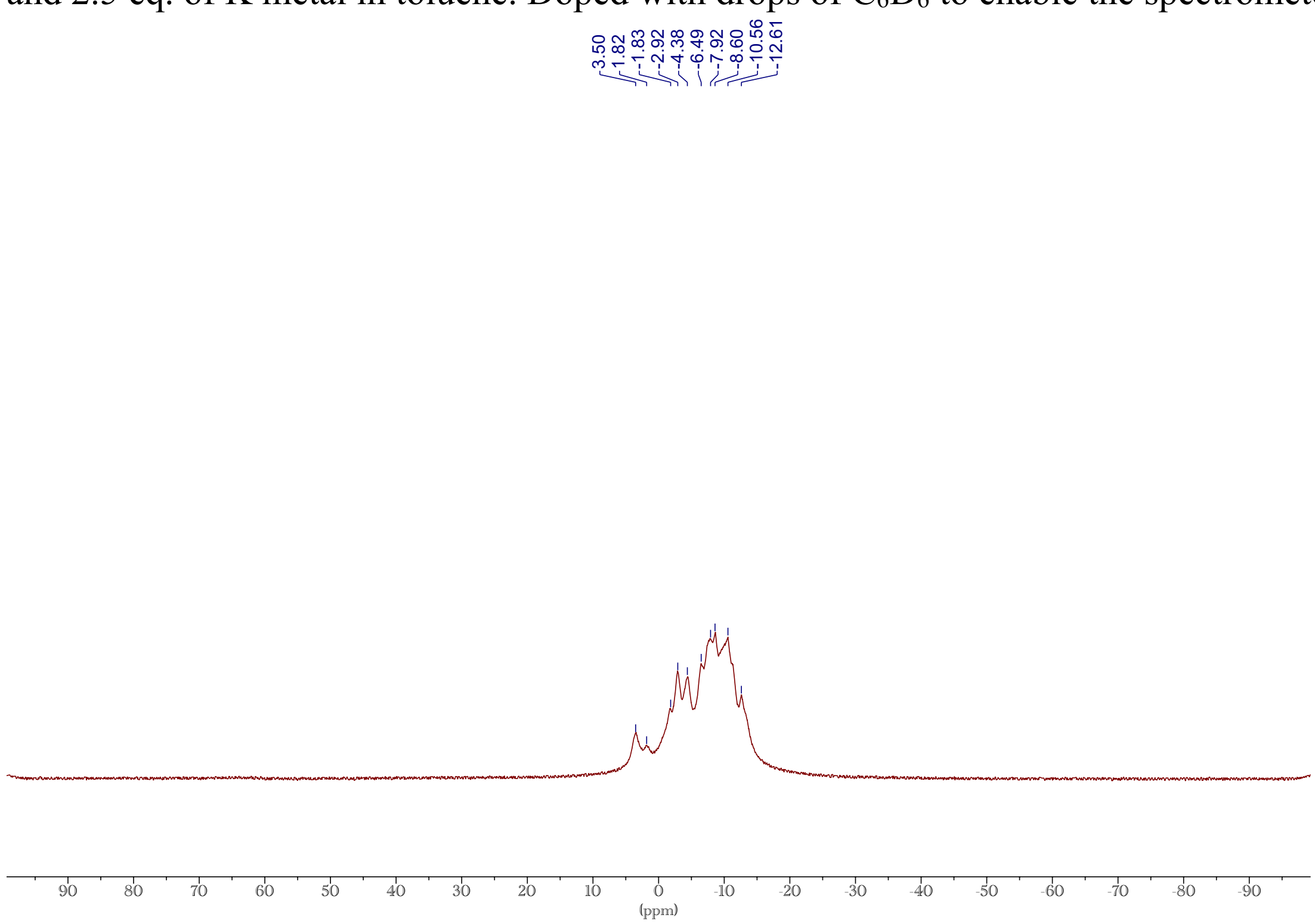

Figure S-33: ^{11}B NMR (128 MHz) spectrum of the crude reaction mixture of $BrB^{Ph}o Cb_2$ and 2.5 eq. of K metal in toluene. Doped with drops of C_6D_6 to enable the spectrometer to lock.

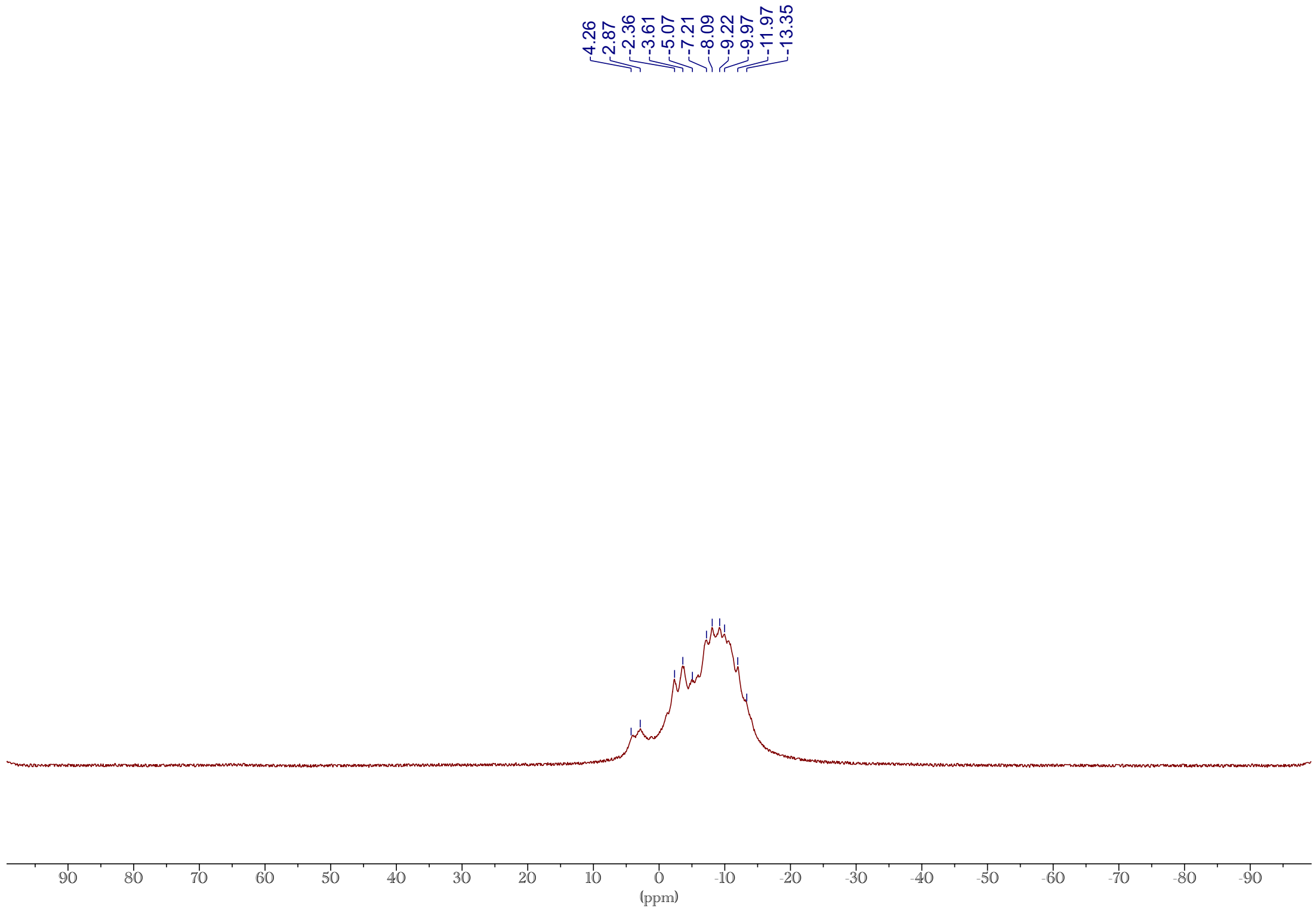

Figure S-34: $^{11}B\{^1H\}$ NMR (128 MHz) spectrum of the crude reaction mixture of $BrB^{TMS}o Cb_2$ and 2.5 eq. of K metal in toluene. Doped with drops of C_6D_6 to enable the spectrometer to lock.

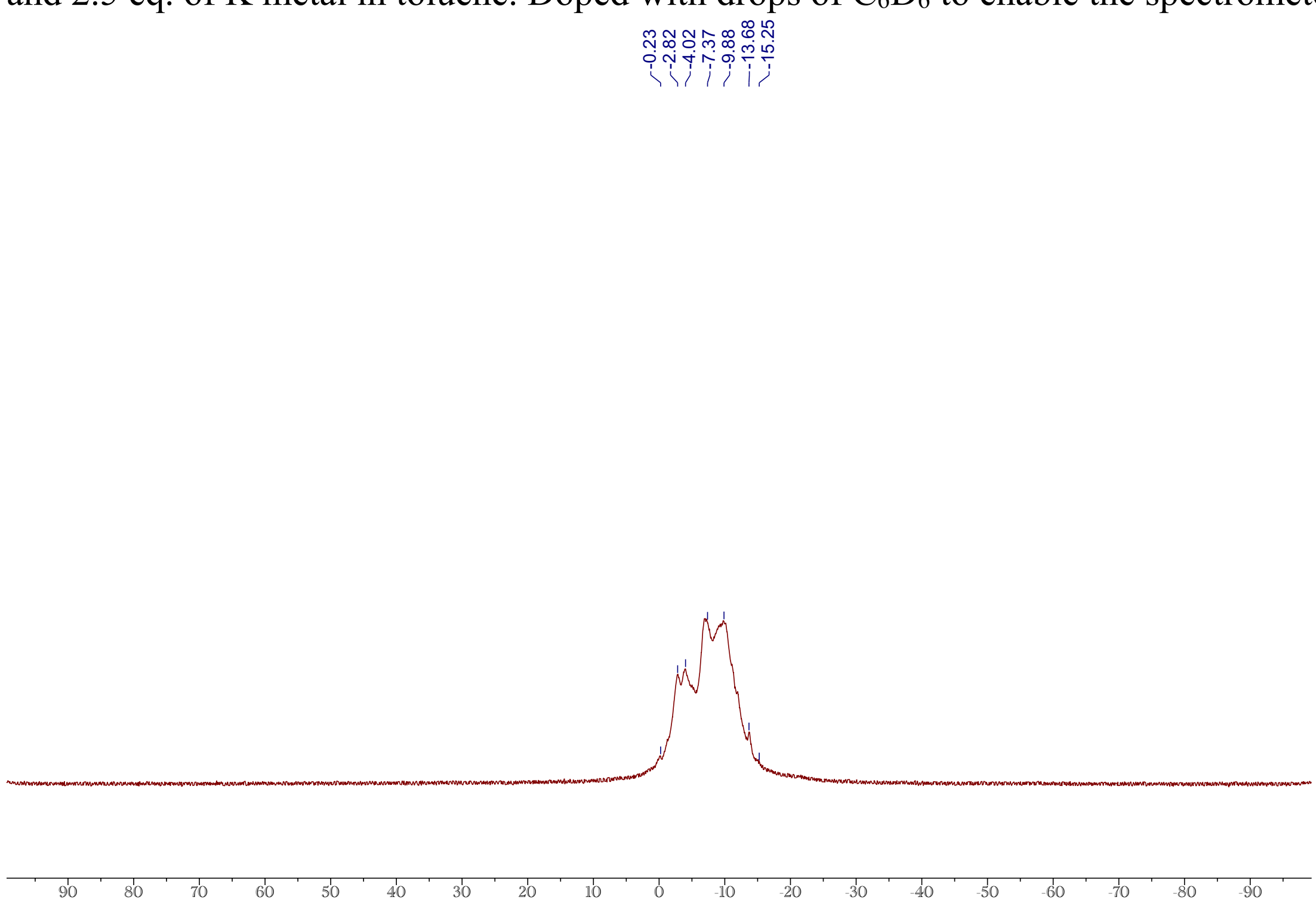

Figure S-35: ^{11}B NMR (128 MHz) spectrum of the crude reaction mixture of BrB^{TMS}*o*Cb_2 and 2.5 eq. of K metal in toluene. Doped with drops of C_6D_6 to enable the spectrometer to lock.

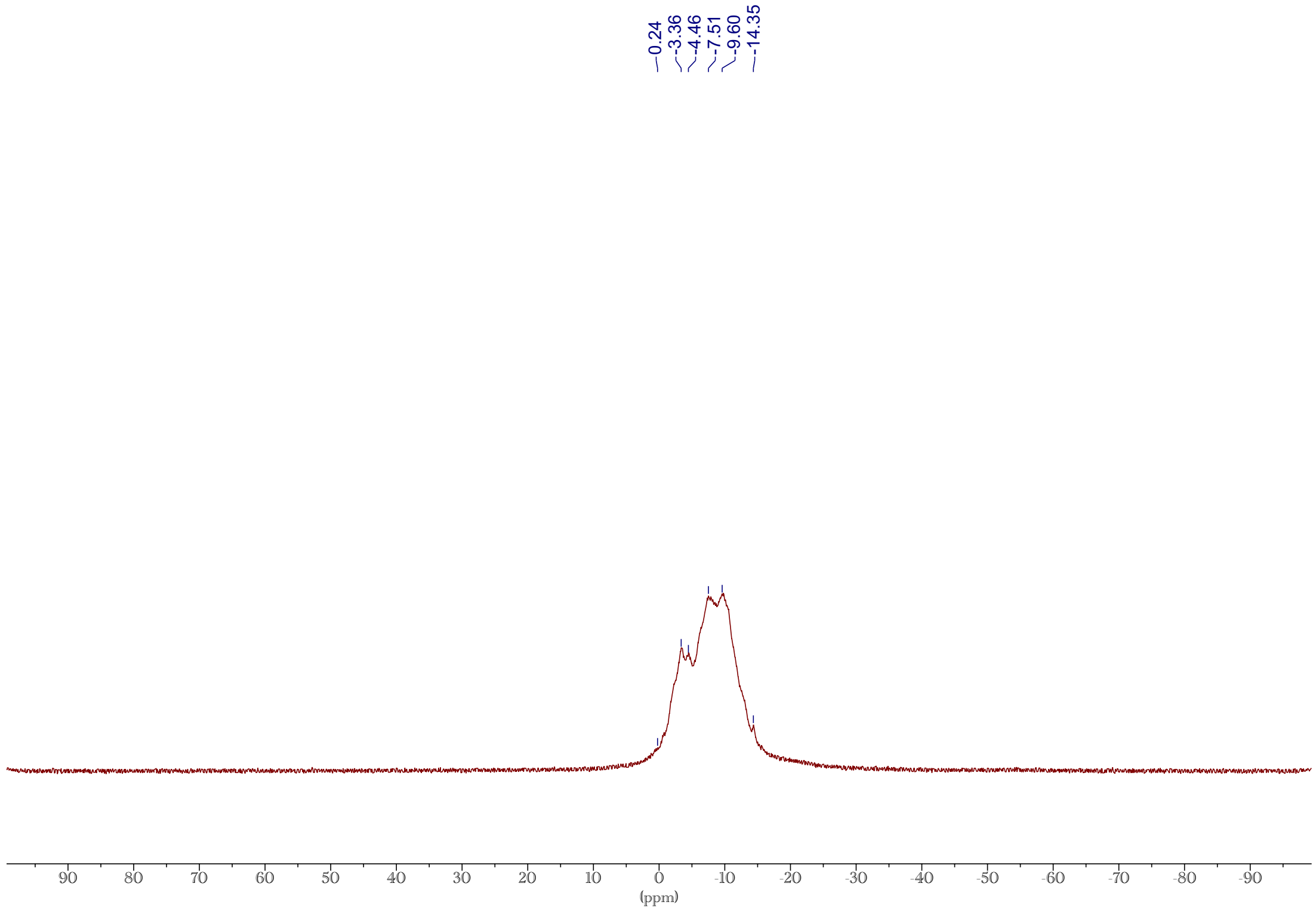

Figure S-36: $^{11}B\{^1H\}$ NMR (128 MHz) spectrum of the crude reaction mixture (BrB^{Me}*o*Cb_2 and 2.5 eq. of K metal in toluene). Doped with drops of C_6D_6 to enable the spectrometer to lock.

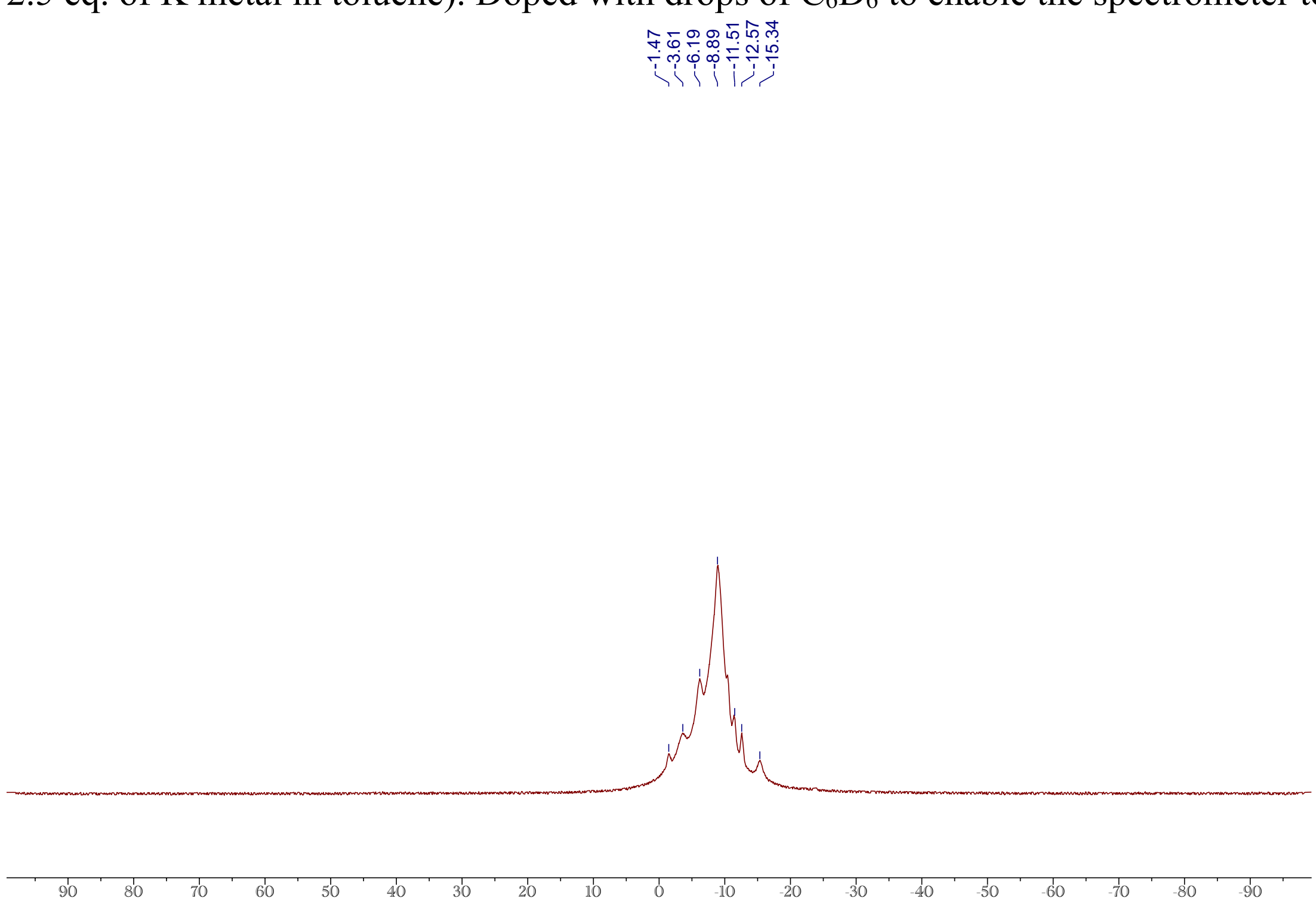

Figure S-37: ^{11}B NMR (128 MHz) spectrum of the crude reaction mixture of BrB^{Me}*o*Cb$_2$ and 2.5 eq. of K metal. Doped with drops of C_6D_6 to enable the spectrometer to lock.

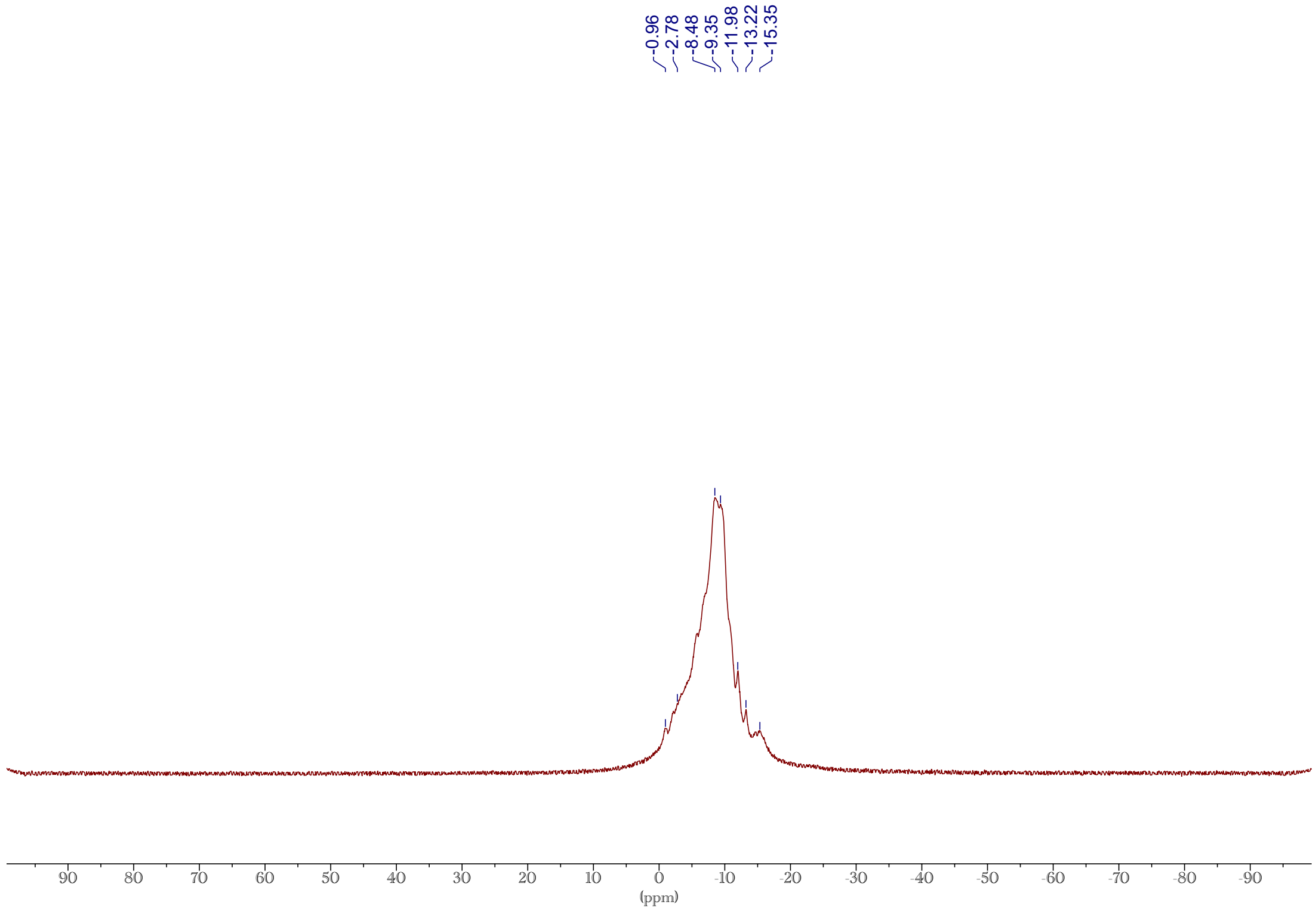

Figure S-38: $^{11}B\{^{1}H\}$ NMR (128 MHz) spectrum of the crude reaction mixture (BrB^{Me}*o*Cb$_2$ and 2.5 eq. of K metal) with few drops of C_7D_8 at low temp (–50 ºC).

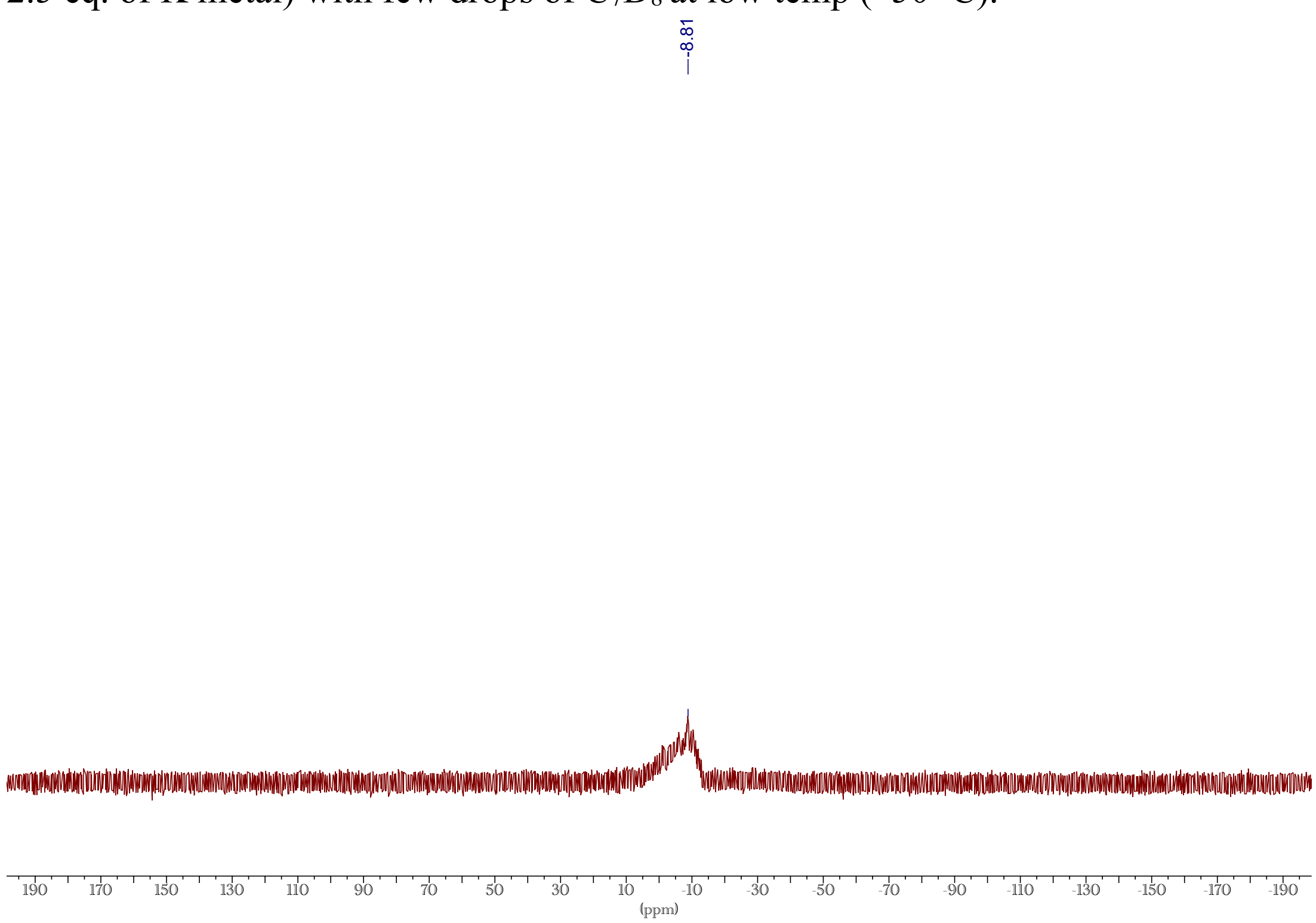

Figure S-39: $^{11}B\{^{1}H\}$ NMR (128 MHz) spectrum of the crude reaction mixture of $BrB^{Me}oCb_2$ and 2.5 eq. of K metal with few drops of C_7D_8 at low temp (–50 ºC).

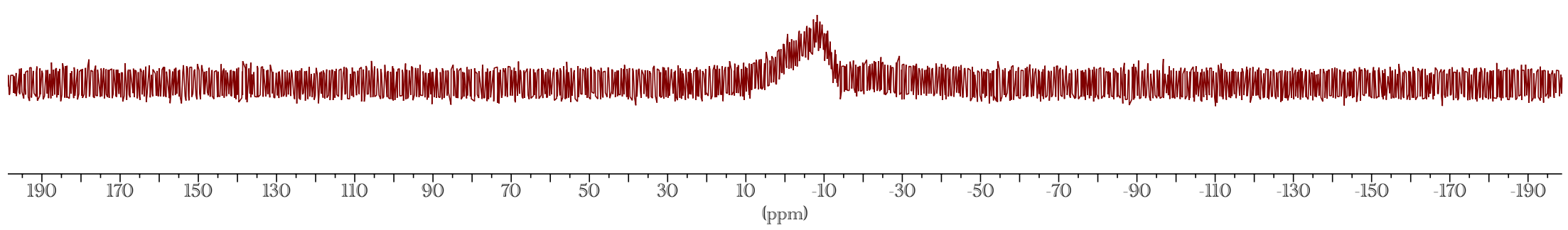

Figure S-40: Solid state structure of $\mathbf{BrB^{TMS}\boldsymbol{o}Cb_2}$. Ellipsoids depicted at the 50% probability level and hydrogen atoms omitted for clarity. Selected bond lengths (Å) and angles (°): $\mathbf{BrB^{TMS}\boldsymbol{o}Cb_2}$: B(1)–C(1) 1.600(7), B(1)–C(3) 1.589(7), B(1)–Br(1) 1.910(5), C(1)–B(1)–C(3) 126.8(3), C(1)–B(1)–Br(1) 116.4(3).

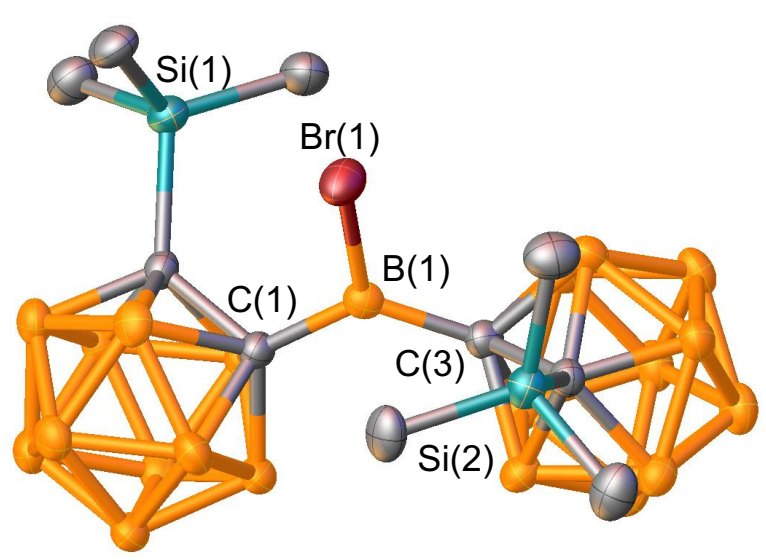

Figure S-41: X-ray structures of **K-crown[1]**, **K[2]**, and **4** with cations.

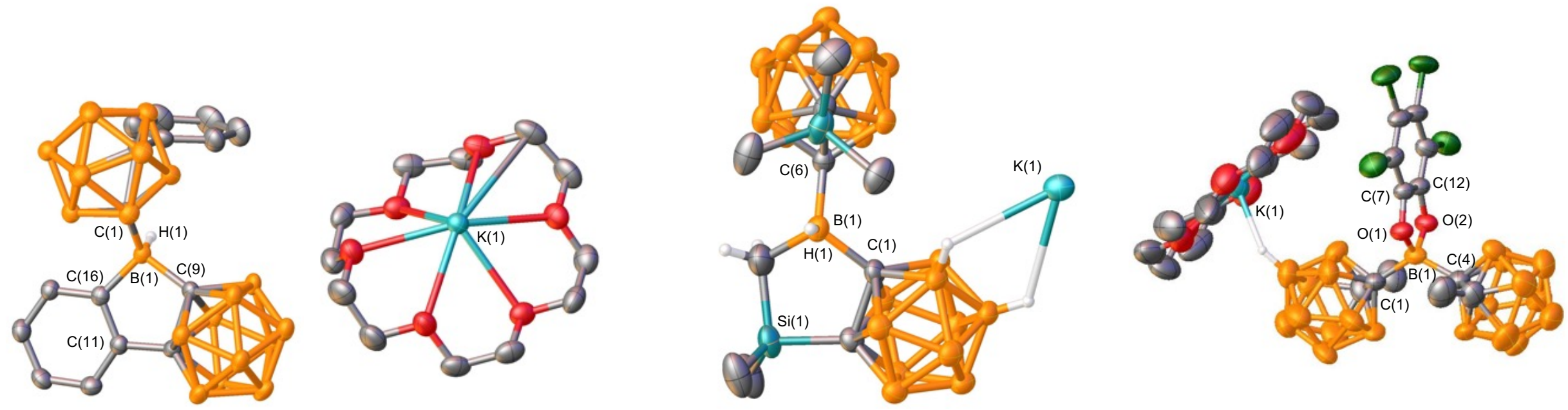

Figure S-42: Linear polymerized X-ray structures of **4**.

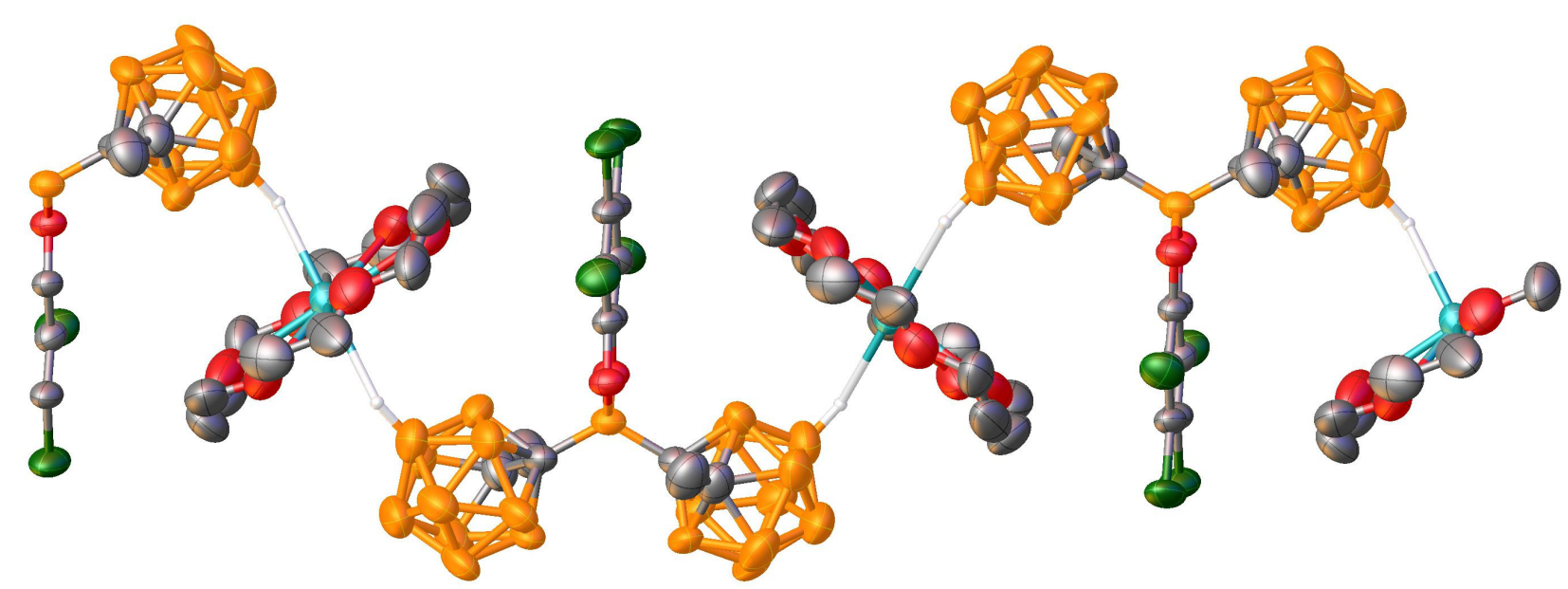

Figure S-43: X-ray structures of **[K][H_2B^{Me}*o*Cb_2]**. Ellipsoids depicted at the 50% probability level and hydrogen atoms omitted for clarity except on the central boron and the ones showing interaction with the cation. Selected bond lengths (Å) and angles (°): **[K][H_2B^{Me}*o*Cb_2]**: B(1)–C(1) 1.644(3), B(1)–C(4) 1.652(3), C(1)–B(1)–C(4) 121.71(15).

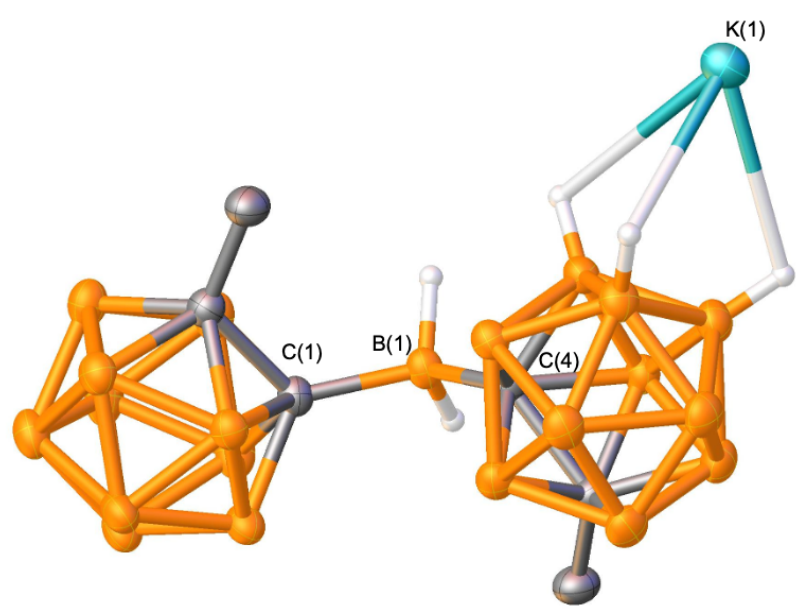

Table S-1: X-ray crystallographic details.

Compound	K-crown[1]	$BrB^{TMS}oCb_2$	K[2]	3	4	$[K][H_2B^{Me}oCb_2]$
**CCDC**	2482457	2482458	2520596	2482460	2504475	2482461
**Empirical Formula**	$C_{68}H_{132}B_{42}K_2O_{18}$	$C_{10}H_{38}B_{21}Si_2Br$	$C_{41}H_{100}B_{42}Si_4K_2$	$Cu_2P_2B_{42}C_{55}H_{90}$	$C_{24}H_{42}KB_{21}O_8Cl_4K$	$C_6H_{28}B_{21}K_1$
**FW (g/mol)**	1769.95	521.797	1237.78	1394.30	879.654	366.39
**Crystal System**	triclinic	monoclinic	monoclinic	monoclinic	monoclinic	orthorhombic
**Space Group**	*P1*	*P2₁/n*	*P2₁/c*	*P2₁/n*	*I2/a*	*Pbca*
**a (Å)**	11.9698(8)	14.195(2)	13.0614(2)	11.3130(10)	15.206(4)	13.5854(12)
**b (Å)**	14.6251(9)	10.4892(14)	32.0813(5)	13.3497(2)	12.018(4)	13.7659(15)
**c (Å)**	16.1758(11)	18.721(3)	19.8194(3)	24.8346(3)	24.163(9)	22.604(2)
**α (deg)**	64.822(2)	90	90	90	90	90
**β (deg)**	76.409(2)	92.413(6)	103.8242(18)	90.8640(10)	97.854(8)	90
**γ (deg)**	72.142(2)	90	90	90	90	90
**V (Å^3)**	2421.2(3)	2785.1(7)	8064.3(2)	3750.22(8)	4374.0(2)	4227.2(7)
**Z**	1	4	4	2	4	8
**Dc (g cm^{-3})**	1.214	1.244	1.020	1.235	1.336	1.151
**Radiation λ (Å)**	0.71073	0.71073	1.54184	1.54184	0.71073	0.71073
**Temp**	150 K	150 K	150 K	150 K	150 K	150 K
**R1 [I>2(σ)I]^a**	0.0585	0.0650	0.1320	0.0435	0.0970	0.0497
**wR2 (F^2)^a**	0.1667	0.1977	0.4161	0.1349	0.3100	0.1633
**GOF (S)^a**	1.029	1.051	1.760	1.049	1.070	1.048

^{a} $R1(F[\mathrm{I} > 2(\mathrm{I})]) = \sum\|F_o| - |F_c\|/\sum|F_o|$; $wR2(F^2$ [all data]$) = \{[w(F_o^2 - F_c^2)^2]/[w(F_o^2)^2]\}^{1/2}$; S(all data) $= [w(F_o^2 - F_c^2)^2/(n - p)]^{1/2}$ (n = no. of data; p = no. of parameters varied; $w = 1/\sigma^2(F_o^2) + (aP)^2 + bP]$ where $P = (F_o^2 + 2F_c^2)/3$ and a and b are constants suggested by the refinement program.

3. Theoretical Calculations

3.1. Computational Methods

All calculations were performed with the Gaussian 16 Rev.C.01 software.[5] The long-range corrected hybrid density functional of ωB97X-D[6] was implemented with the Karlsruhe basis sets of valence triple-zeta with two sets of polarization functions (def2-TZVPP).[7, 8] Geometry optimizations were performed prior to harmonic frequency and population analysis runs. Both gas phase (vacuum) and toluene phase using implicit solvent model based on solvation model based on density (SMD) developed by Truhlar and co-workers were computed.[9] The vibrational contributions to the free-energy were implemented through calculating harmonic frequencies. Harmonic vibrational frequency calculations were used to confirm the nature of stationary points as minima or transition states and to obtain thermal corrections to Gibbs free energies. Frontier molecular orbitals were analyzed from the converged electronic wavefunctions of the optimized structures; the orbitals were not computed from frequency calculations. Wiberg bond index (WBI), natural population, Fukui-function, wavefunction-stability, broken-symmetry, natural-orbital occupation, and protonation-site analyses were carried out at the same level unless otherwise stated. All molecular structure calculations were computed at singlet and triplet states for comparison. Population analyses based on Natural Bond Orbital (NBO)[10] and the restrained electrostatic potential (RESP)[11] were implemented to assess bond orders and charge states of the centered boron and other atoms within each carborane complexes. All visualizations were assessed with the VMD software.[12] The frontier molecular orbitals and condensed Fukui functions are calculated with the Multiwfn software[13].

3.2. Closed-Shell vs. Open-Shell Singlet Character

Evaluation of possible open-shell singlet character was assessed. Because an open-shell singlet/diradicaloid electronic structure could affect the relative energies of the boryl anion and C–H insertion transition states, we performed wavefunction-stability analyses, broken-symmetry unrestricted singlet calculations, and natural-orbital occupation analyses for the boryl anions ground states. The closed-shell singlet solutions for $B^{Me}oCb_2^-$, $B^{Ph}oCb_2^-$, and $B^{TMS}oCb_2^-$ were stable with respect to internal and external instabilities. Broken-symmetry unrestricted singlet calculations initiated from spin-polarized initial guesses collapsed back to closed-shell solutions, with final $\langle S^2 \rangle$ values of 0.000–0.002. Natural-orbital occupation numbers are close to the ideal closed-shell limit of 2.0/0.0 for the highest occupied natural orbital (HONO)/lowest unoccupied natural orbital (LUNO) pair and far from the approximately 1.0/1.0 occupation pattern expected for a singlet diradical. The calculated HONO and LUNO occupations are 1.986/0.014 for $B^{Me}\textit{o}Cb_2^-$, 1.982/0.018 for $B^{Ph}\textit{o}Cb_2^-$, and 1.980/0.020 for $B^{TMS}\textit{o}Cb_2^-$. This confirms that the boryl anion ground state is not a singlet diradical (open-shell singlet), but rather it is a closed-shell singlet.

3.3. Evaluation of possible B–B-coupled diborane formation

The possibility of reductive B–B coupling was considered computationally by optimizing the neutral diborane $^{Me}oCb_2B–B^{Me}oCb_2$ at the ωB97X-D/def2-TZVPP/SMD(toluene) level. We note that direct comparison of this diborane with $BrB^{Me}oCb_2$ alone is not a balanced redox reaction unless the reductant and bromide-containing byproducts are explicitly included; therefore, such a thermodynamic comparison was not used as a quantitative criterion. Instead, the optimized structure was used to assess whether a structurally reasonable B–B-bonded product is accessible. The optimized diborane is highly congested and displays an elongated B–B distance of 2.04 Å with a low B–B Wiberg bond index of 0.43. These values indicate a weak and sterically

compromised B–B interaction. These calculations argue against significant reductive B–B coupling under the reaction conditions.

3.4. Computational Assessment of Basicity

Because the free boryl anion is transient and could not be isolated for direct experimental basicity measurement, relative protonation free energies were computed at the ωB97X-D/def2-TZVPP/SMD(toluene) level. Protonation at the central boryl boron is strongly preferred. Relative to central-boron protonation, protonation at the carborane cage carbon is higher in free energy by 18.7 kcal mol^{-1}, while protonation at the methyl substituent is higher by 26.4 kcal mol^{-1}. These results, together with the B-centered HOMO, natural charge at boron of −0.42e, and condensed Fukui value f_{B}^{-} = 0.35, support assignment of B^{Me}*o*Cb_2^- as a B-centered base/nucleophile. The carboranyl substituents nevertheless moderate its reactivity through electron-withdrawing stabilization and steric protection, consistent with successful trapping by $Cu(PPh_3)Cl$ and tetrachloro-o-benzoquinone but decomposition with CO_2 and fluorobenzene.

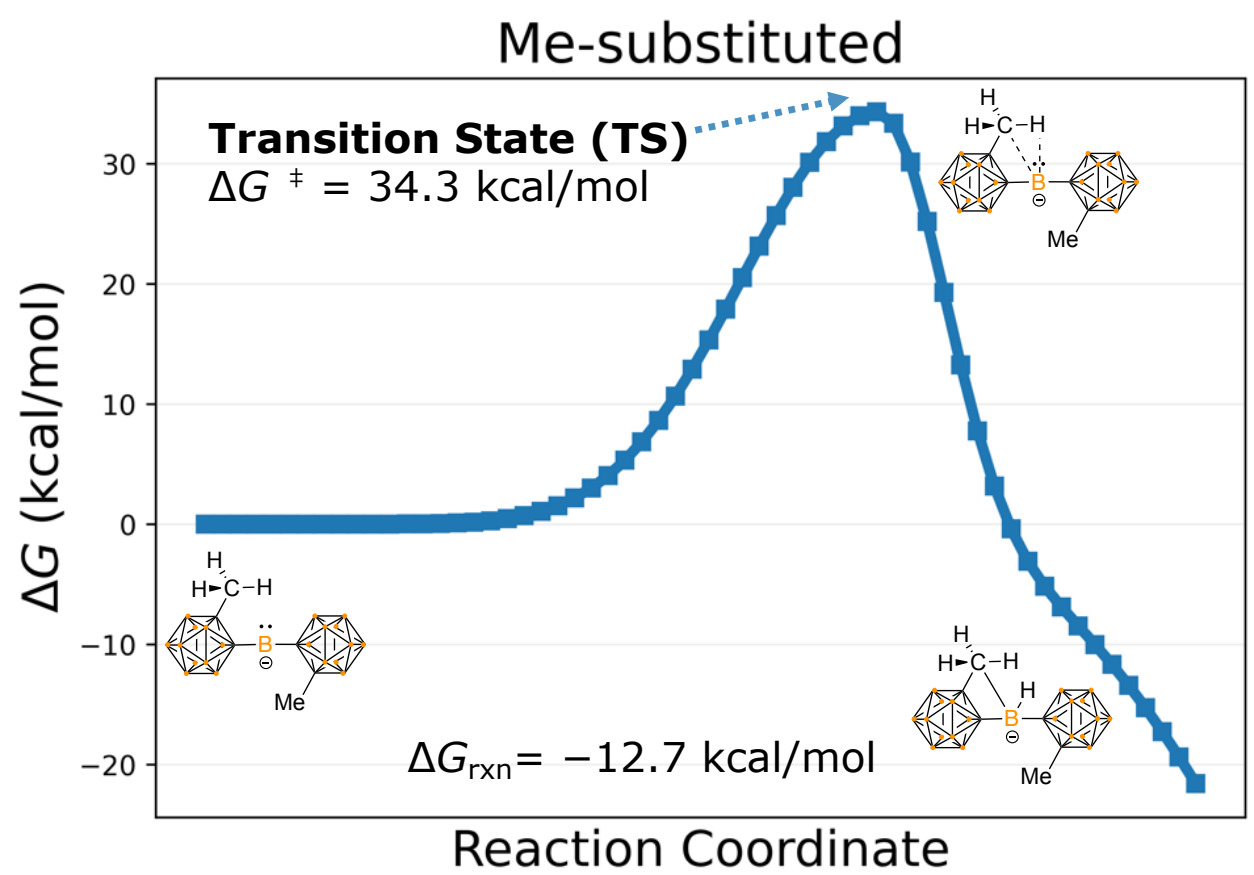

Figure S-36: Free-energy diagrams (ΔG in kcal/mol) for intramolecular C-H insertion in case of $B^{Me}oCb_2$ and cyclization to form a four-membered boracycle exhibit the highest free-energy of activation ($\Delta G^{\ddagger}$) of 34.3 kcal/mol, yet the most thermodynamically favorable product with ΔG_{rxn} of −12.7 kcal/mol.

Table S-2. The optimized structures with XYZ cartesian coordinates, HOMO, and LUMO, all calculated at the wB97X-D/def2-TZVPP level of theory.

[B^{Ph}*o*Cb_2]$^-$ singlet, gas phase

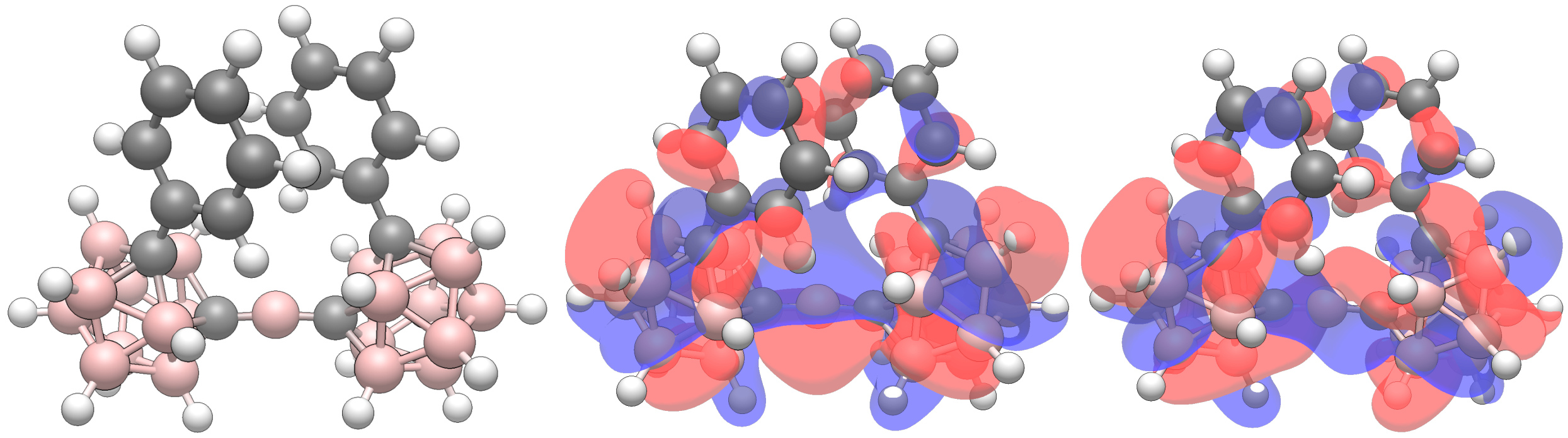

C	1.423874	-1.777890	0.095351
C	2.747126	0.115021	-0.226408
C	-1.423859	-1.777907	-0.095188
C	-2.143161	1.426932	0.575681
C	-2.747122	0.115031	0.226395
B	2.320426	-2.490819	1.254867
H	1.737059	-3.109680	2.075593
B	-2.453045	-2.906642	0.503709
H	-1.937526	-3.823748	1.040311
B	-3.892983	-2.955909	-0.532363
H	-4.371785	-4.003964	-0.808333
B	-3.777384	-1.591497	-1.685396
H	-4.166378	-1.686256	-2.799950
B	0.000007	-1.691260	0.000076
B	4.019974	-0.086763	0.797635
H	4.500901	0.862861	1.309462
B	4.101434	-0.472254	-0.930486
H	4.618146	0.194436	-1.758612
B	2.332006	-0.630551	1.227491
H	1.668759	-0.041559	2.005386
B	-3.953568	-2.232711	1.097144
H	-4.466030	-2.766880	2.021383
B	3.893000	-2.955837	0.532661
H	4.371807	-4.003861	0.808736
B	4.882274	-1.535457	0.260481
H	6.063474	-1.587836	0.339072
B	2.453069	-2.906679	-0.503426
H	1.937558	-3.823841	-1.039942
B	3.777385	-1.591312	1.685559
H	4.166372	-1.685959	2.800124
B	-4.101422	-0.472184	0.930540
H	-4.618133	0.194586	1.758604
B	-2.439884	-1.218334	1.235909

H	-1.917271	-1.045966	2.279178
B	3.953592	-2.232799	-1.096917
H	4.466064	-2.767056	-2.021100
B	-4.019977	-0.086862	-0.797619
H	-4.500914	0.862707	-1.309537
B	2.439903	-1.218445	-1.235794
H	1.917297	-1.046182	-2.279084
B	-4.882264	-1.535509	-0.260317
H	-6.063464	-1.587902	-0.338895
B	-2.332007	-0.630681	-1.227433
H	-1.668770	-0.041761	-2.005391
B	-2.320417	-2.490953	-1.254627
H	-1.737053	-3.109891	-2.075297
C	-2.700055	2.624832	0.106925
C	-2.154297	3.852047	0.466115
C	-1.022866	3.910269	1.277431
C	-0.443974	2.727676	1.724868
C	-1.001921	1.500188	1.383353
H	-0.586388	4.866501	1.541680
H	0.455873	2.752693	2.327622
H	-0.541570	0.593878	1.746955
H	-3.577593	2.590275	-0.524211
H	-2.611916	4.766630	0.104041
C	2.143154	1.426881	-0.575824
C	1.001910	1.500047	-1.383500
C	0.443952	2.727496	-1.725135
C	1.022834	3.910139	-1.277817
C	2.154268	3.852007	-0.466498
C	2.700038	2.624833	-0.107188
H	3.577578	2.590345	0.523948
H	0.541565	0.593697	-1.747010
H	-0.455897	2.752446	-2.327889
H	0.586347	4.866341	-1.542159
H	2.611879	4.766631	-0.104515

[B^{Ph}*o*Cb$_2$]$^-$ singlet, toluene phase (with SMD)

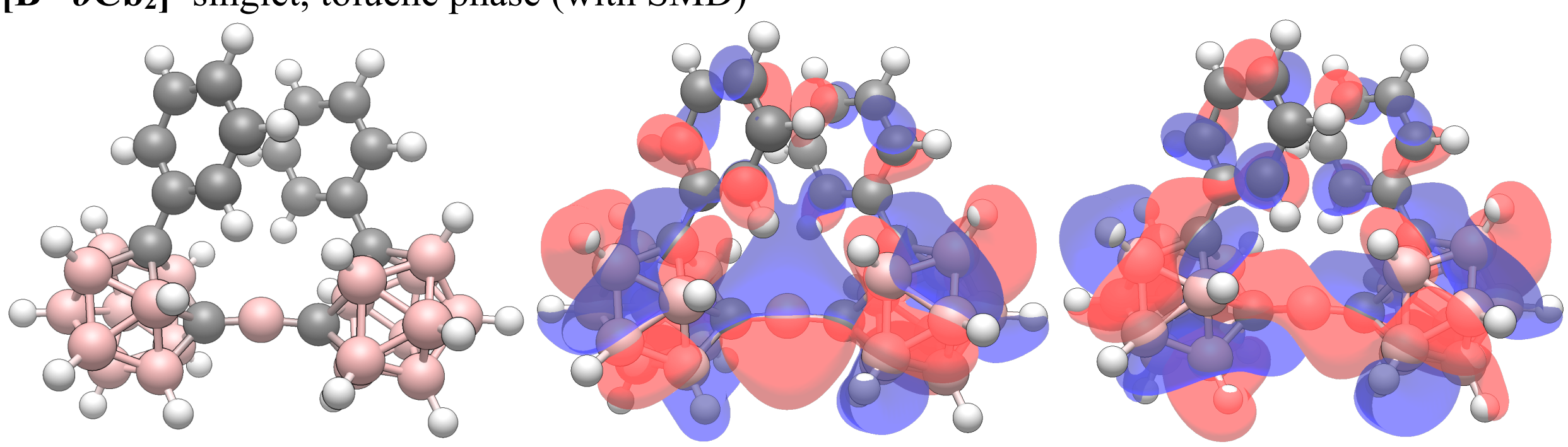

C	1.393692	-1.784799	0.099284

C	2.751799	0.092134	-0.221935
C	-1.453447	-1.751465	-0.091525
C	-2.136547	1.442962	0.575311
C	-2.754648	0.138094	0.223947
B	2.280388	-2.512340	1.256243
H	1.690648	-3.124147	2.077605
B	-2.484385	-2.883355	0.499962
H	-1.974439	-3.802849	1.037013
B	-3.919387	-2.924959	-0.542842
H	-4.405146	-3.969097	-0.820721
B	-3.788622	-1.562338	-1.694901
H	-4.175744	-1.652991	-2.810249
B	-0.026754	-1.676372	0.005671
B	4.022582	-0.136431	0.799168
H	4.524284	0.800956	1.311606
B	4.093226	-0.521419	-0.928387
H	4.624008	0.133198	-1.756772
B	2.323409	-0.650834	1.228157
H	1.671735	-0.053620	2.008698
B	-3.984895	-2.202570	1.085538
H	-4.508199	-2.733252	2.005561
B	3.842307	-3.002700	0.531024
H	4.305561	-4.058359	0.804687
B	4.855732	-1.599634	0.258837
H	6.035738	-1.675949	0.335871
B	2.402826	-2.928506	-0.502181
H	1.874191	-3.838737	-1.037487
B	3.751912	-1.637502	1.684643
H	4.142104	-1.741637	2.797982
B	-4.116492	-0.440897	0.919587
H	-4.634438	0.227569	1.744745
B	-2.466509	-1.199631	1.237252
H	-1.951153	-1.034877	2.284221
B	3.912225	-2.278202	-1.096730
H	4.414659	-2.820405	-2.021964
B	-4.020984	-0.055181	-0.807900
H	-4.491440	0.896796	-1.322696
B	2.416314	-1.236215	-1.229580
H	1.897809	-1.057437	-2.273096
B	-4.898843	-1.496540	-0.276031
H	-6.079685	-1.539622	-0.362417
B	-2.336437	-0.613979	-1.230700
H	-1.667020	-0.029382	-2.005438
B	-2.341474	-2.471055	-1.255429
H	-1.753528	-3.093587	-2.069524
C	-2.666256	2.645897	0.086815

C	-2.114238	3.867390	0.456271
C	-1.002107	3.913809	1.295413
C	-0.446679	2.725256	1.758209
C	-1.011766	1.503135	1.407314
H	-0.564289	4.865888	1.571931
H	0.434916	2.742426	2.387898
H	-0.572482	0.592484	1.786649
H	-3.529403	2.623267	-0.564753
H	-2.552186	4.786249	0.081704
C	2.174827	1.417455	-0.569602
C	1.050902	1.515126	-1.399482
C	0.525434	2.755163	-1.749434
C	1.119068	3.925104	-1.286251
C	2.229857	3.842122	-0.448282
C	2.743387	2.602797	-0.081657
H	3.607001	2.552090	0.567725
H	0.581863	0.618999	-1.777958
H	-0.355520	2.801178	-2.378666
H	0.711901	4.891039	-1.561897
H	2.697909	4.746021	-0.073643

[B^{Ph}*o*Cb$_2$]$^-$ triplet, gas phase

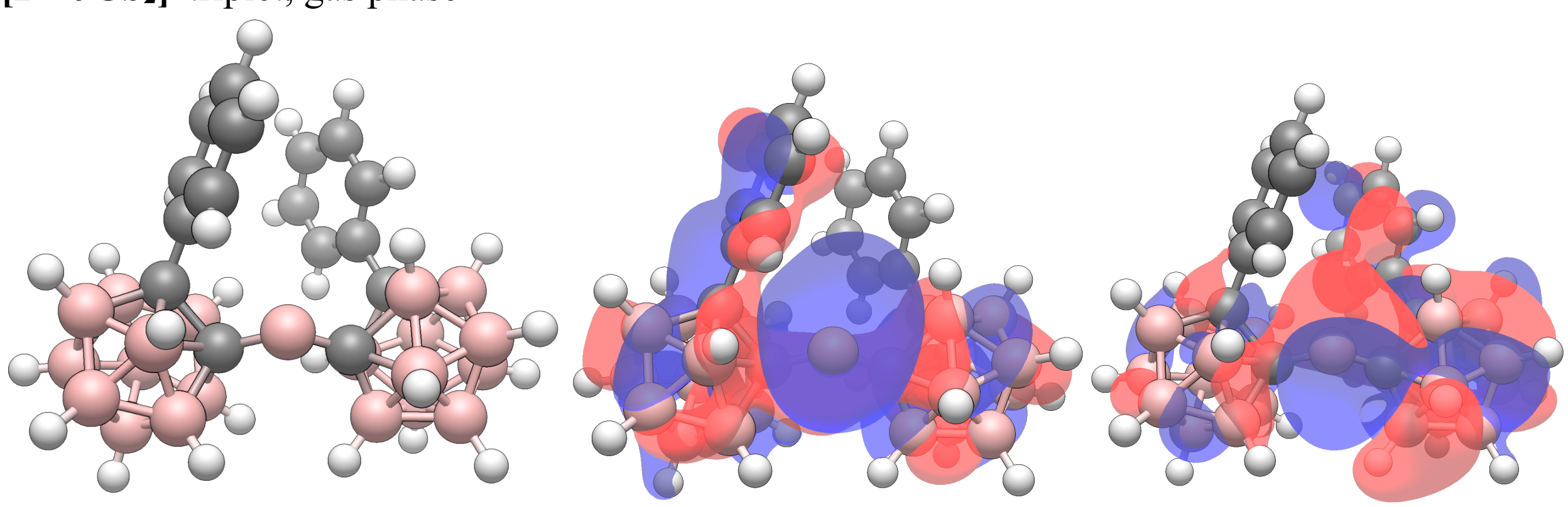

C	-1.447218	-1.357195	0.250454
C	-2.591850	-0.056461	0.223999
C	-2.159981	1.255434	0.812462
C	1.302885	-1.415408	0.762321
C	2.050900	1.394295	-0.811244
C	2.603193	0.119420	-0.313933
B	-1.634676	-2.298171	-1.173783
H	-0.669745	-2.799204	-1.619593
B	2.019150	-2.792529	0.234962
H	1.313505	-3.728415	0.078368
B	3.677117	-2.834675	0.886160
H	4.133447	-3.855285	1.282113
B	3.987843	-1.254646	1.666266
H	4.657462	-1.148636	2.638871

B	-0.122423	-1.096732	1.009409
B	-3.499526	-0.115559	-1.207688
H	-3.841484	0.904746	-1.688299
B	-4.184351	-0.652529	0.334220
H	-4.965748	0.015574	0.913228
B	-1.767880	-0.533319	-1.219483
H	-0.946262	0.189980	-1.639492
B	3.383563	-2.493772	-0.845103
H	3.614170	-3.287141	-1.694920
B	-3.270267	-2.968898	-1.100155
H	-3.510449	-4.029038	-1.567996
B	-4.426157	-1.621812	-1.131054
H	-5.497388	-1.698379	-1.629296
B	-2.296059	-2.827088	0.379252
H	-1.790341	-3.693076	0.999204
B	-2.931005	-1.534152	-2.101207
H	-2.930896	-1.542275	-3.284176
B	3.688993	-0.785396	-1.146408
H	4.040249	-0.370428	-2.197676
B	1.973561	-1.362297	-0.887586
H	1.210174	-1.344703	-1.783700
B	-4.025899	-2.413848	0.417223
H	-4.795356	-3.064962	1.036839
B	4.061614	-0.012715	0.422663
H	4.692663	0.981012	0.546705
B	-2.864304	-1.394121	1.254067
H	-2.732957	-1.279311	2.414141
B	4.673753	-1.643533	0.060300
H	5.830889	-1.820495	-0.130446
B	2.504774	-0.260274	1.354696
H	2.125833	0.567634	2.106364
B	2.401116	-2.013582	1.817302
H	1.990806	-2.356284	2.872585
C	-2.006453	2.370581	-0.015870
C	-1.617241	3.596945	0.510505
C	-1.354306	3.724548	1.871259
C	-1.481358	2.614008	2.700083
C	-1.881261	1.387633	2.176163
H	-1.967177	0.532687	2.828421
H	-1.036488	4.677561	2.279422
H	-1.259212	2.693310	3.758033
H	-2.178555	2.278017	-1.078508
H	-1.495890	4.444743	-0.151878
C	1.531203	1.498339	-2.110300
C	1.050597	2.710186	-2.594306
C	1.079913	3.850204	-1.792783

C	1.586252	3.758719	-0.497651
C	2.059232	2.546155	-0.010415
H	2.444115	2.483483	0.998315
H	0.705697	4.795563	-2.170613
H	1.603579	4.633953	0.142447
H	1.510514	0.620040	-2.741008
H	0.653115	2.763150	-3.602179

[B^{Ph}*o*Cb$_2$]$^-$ triplet, toluene phase (with SMD)

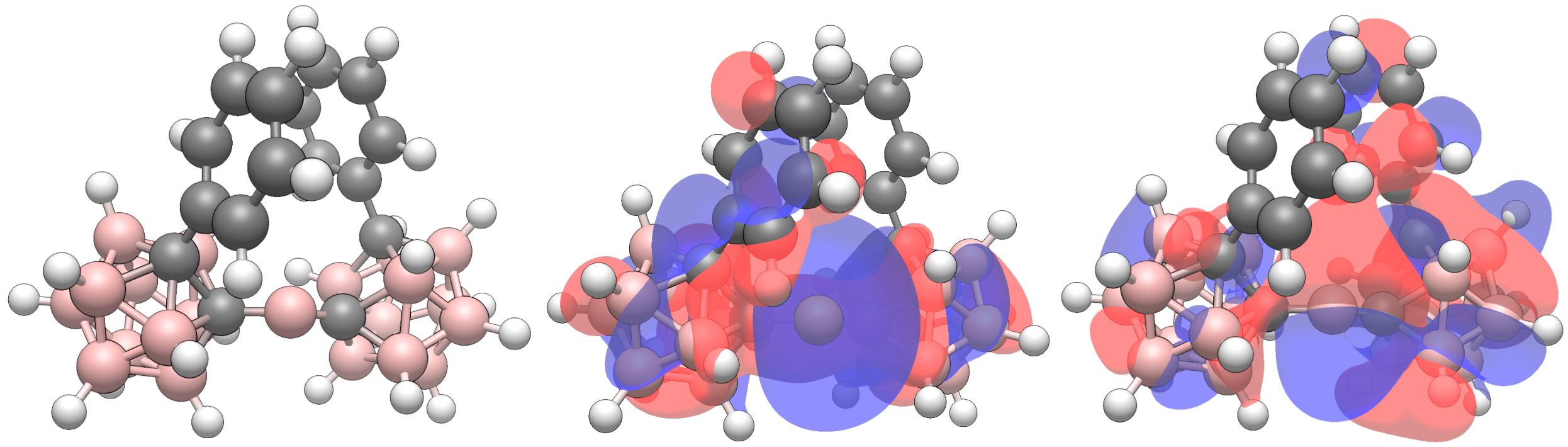

C	-1.436904	-1.337049	0.227530
C	-2.617366	-0.076510	0.196440
C	-2.239151	1.253239	0.784194
C	1.300874	-1.383305	0.771429
C	2.123625	1.420127	-0.788997
C	2.650089	0.135542	-0.288310
B	-1.583651	-2.288539	-1.194336
H	-0.601466	-2.759343	-1.634698
B	1.998190	-2.772335	0.250943
H	1.280207	-3.696999	0.084520
B	3.643931	-2.846993	0.924131
H	4.077311	-3.876355	1.323602
B	3.972431	-1.274674	1.711753
H	4.630687	-1.184388	2.693819
B	-0.123987	-1.045199	0.996383
B	-3.516625	-0.168103	-1.239367
H	-3.889621	0.839628	-1.721372
B	-4.189629	-0.726594	0.300370
H	-4.996707	-0.085602	0.873195
B	-1.772426	-0.529321	-1.244776
H	-0.969086	0.213278	-1.666463
B	3.381296	-2.494934	-0.809341
H	3.608340	-3.292760	-1.656155
B	-3.194571	-3.013018	-1.126227
H	-3.396416	-4.081017	-1.593629
B	-4.392870	-1.704943	-1.163619
H	-5.458268	-1.818796	-1.666208
B	-2.231996	-2.836769	0.356233

H	-1.697428	-3.683417	0.978281
B	-2.898663	-1.569254	-2.128153
H	-2.892883	-1.581146	-3.310702
B	3.727582	-0.793339	-1.102956
H	4.108776	-0.388474	-2.147138
B	1.995558	-1.334131	-0.866127
H	1.246945	-1.300284	-1.773556
B	-3.973577	-2.481435	0.386972
H	-4.723535	-3.157120	1.003284
B	4.090549	-0.031444	0.472732
H	4.744505	0.944718	0.611314
B	-2.851294	-1.422291	1.228298
H	-2.723184	-1.303693	2.387365
B	4.674935	-1.672997	0.116373
H	5.830930	-1.874519	-0.056629
B	2.511545	-0.253001	1.377246
H	2.140316	0.578503	2.127728
B	2.371754	-2.005639	1.839115
H	1.942873	-2.347197	2.887302
C	-2.071201	2.358561	-0.055661
C	-1.731072	3.601172	0.467771
C	-1.537806	3.756101	1.837909
C	-1.688473	2.657770	2.679643
C	-2.037809	1.414887	2.158388
H	-2.148336	0.573138	2.824866
H	-1.262002	4.722389	2.244983
H	-1.528563	2.761034	3.746676
H	-2.196460	2.249221	-1.123578
H	-1.602028	4.443058	-0.201211
C	1.630786	1.535906	-2.097969
C	1.167069	2.754612	-2.581936
C	1.182353	3.886839	-1.768461
C	1.662805	3.783599	-0.464056
C	2.123646	2.565257	0.021895
H	2.491135	2.496525	1.037006
H	0.819809	4.836727	-2.145608
H	1.672831	4.654061	0.182442
H	1.617184	0.664334	-2.738613
H	0.792933	2.818777	-3.597916

[B^{TMS}*o*Cb$_2$]$^-$ singlet, gas phase

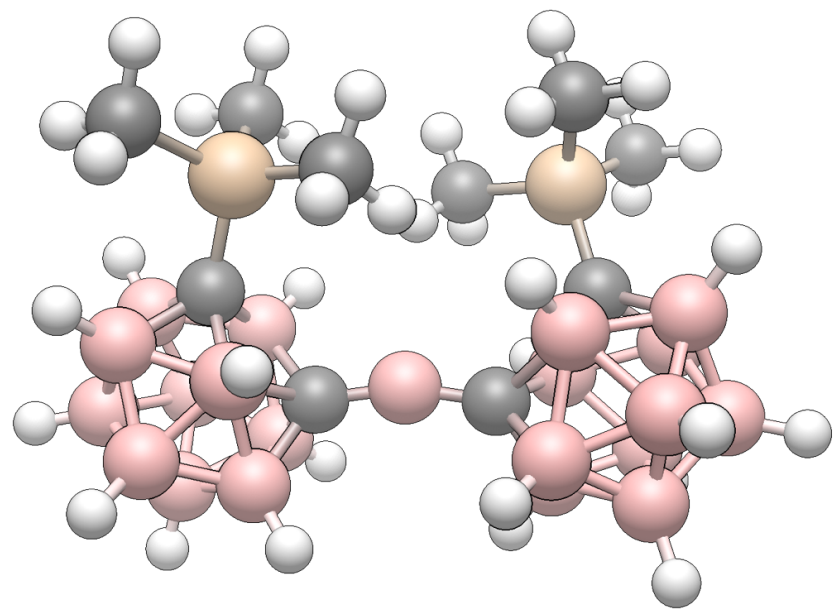

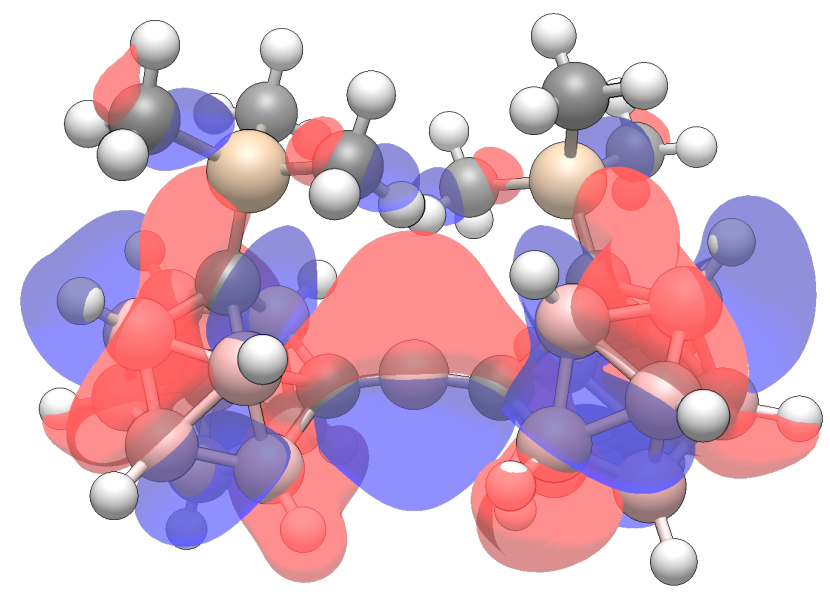

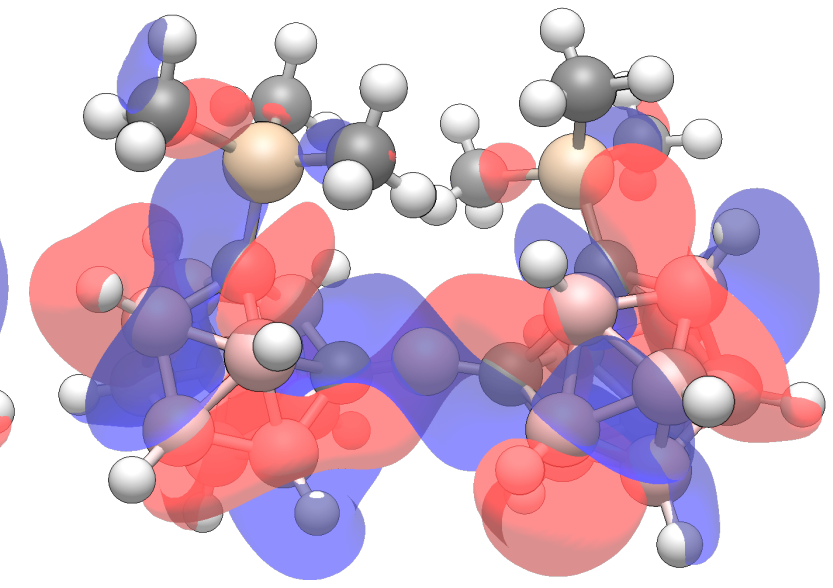

C	1.407057	-1.400679	0.165242
C	3.009937	0.293234	-0.091664
Si	2.597980	2.031180	-0.674421
C	-1.407051	-1.400682	-0.165248
Si	-2.597984	2.031179	0.674420
C	-3.009937	0.293232	0.091665
B	2.104417	-2.148697	1.428643
H	1.380479	-2.637674	2.225612
B	-2.320299	-2.684382	0.295317
H	-1.722678	-3.551776	0.830375
B	-3.649576	-2.869711	-0.867860
H	-3.945572	-3.959620	-1.227787
B	-3.641187	-1.445311	-1.952859
H	-3.917476	-1.547663	-3.100283
B	0.000003	-1.200699	-0.000003
B	4.169447	-0.034851	1.032455
H	4.737582	0.878201	1.529927
B	4.306199	-0.519353	-0.661771
H	4.986055	-0.000252	-1.477973
B	2.390381	-0.308047	1.331239
H	1.744230	0.378267	2.041938
B	-3.933558	-2.248282	0.781234
H	-4.445876	-2.884499	1.639126
B	3.649580	-2.869707	0.867862
H	3.945577	-3.959616	1.227788
B	4.848544	-1.619301	0.622115
H	6.001552	-1.834508	0.793557
B	2.320307	-2.684379	-0.295319
H	1.722688	-3.551773	-0.830380
B	3.641185	-1.445308	1.952862
H	3.917471	-1.547660	3.100286
B	-4.306195	-0.519359	0.661776
H	-4.986050	-0.000260	1.477980
B	-2.579937	-1.033027	1.082195
H	-2.171097	-0.834418	2.173418
B	3.933567	-2.248277	-0.781231
H	4.445889	-2.884493	-1.639122
B	-4.169451	-0.034856	-1.032450

H	-4.737590	0.878195	-1.529920
B	2.579945	-1.033025	-1.082198
H	2.171109	-0.834416	-2.173422
B	-4.848541	-1.619308	-0.622109
H	-6.001550	-1.834517	-0.793547
B	-2.390385	-0.308047	-1.331240
H	-1.744237	0.378268	-2.041940
B	-2.104416	-2.148697	-1.428647
H	-1.380481	-2.637673	-2.225619
C	-0.978388	2.004376	1.628722
C	-3.952155	2.661417	1.825046
C	-2.504613	3.184509	-0.811449
H	-1.785200	2.835042	-1.552571
H	-2.210545	4.190987	-0.496582
H	-3.481444	3.252435	-1.298042
H	-4.042899	2.030720	2.712784
H	-4.923451	2.673882	1.323742
H	-3.721867	3.680997	2.152362
H	-1.110228	1.496561	2.587289
H	-0.626312	3.020837	1.826614
H	-0.192014	1.475408	1.090551
C	3.952148	2.661419	-1.825049
C	2.504609	3.184511	0.811447
C	0.978383	2.004372	-1.628721
H	3.481441	3.252440	1.298038
H	1.785198	2.835043	1.552572
H	2.210538	4.190988	0.496581
H	4.923442	2.673895	-1.323742
H	3.721854	3.680995	-2.152373
H	4.042900	2.030716	-2.712782
H	1.110224	1.496560	-2.587290
H	0.626301	3.020832	-1.826609
H	0.192012	1.475397	-1.090550

[B^{TMS}*o*Cb$_2$]$^-$ singlet, toluene phase (with SMD)

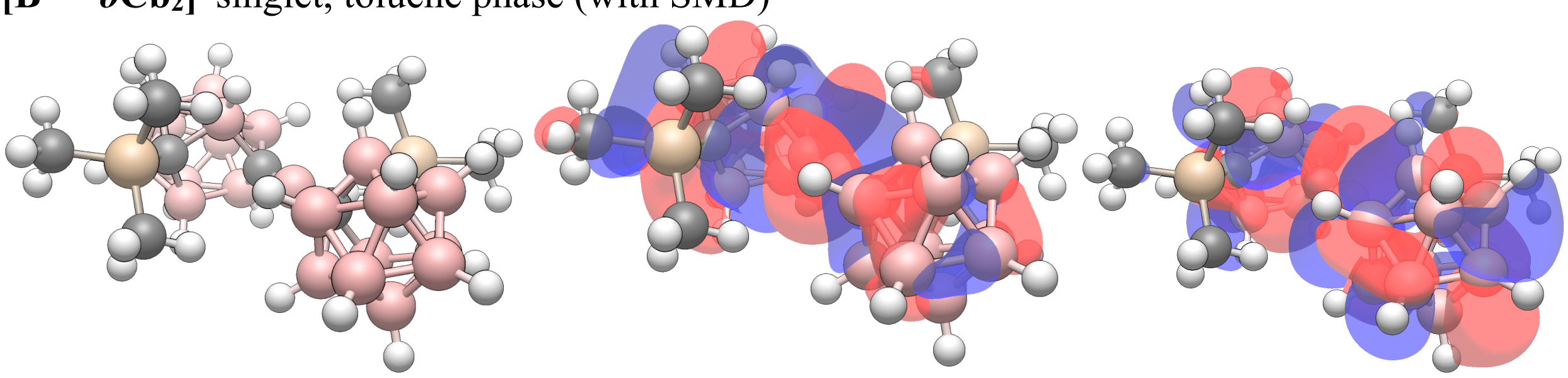

C	1.589579	-0.879511	0.439286
C	2.776766	0.099848	-0.229135
Si	2.515477	1.014013	-1.916897
C	-1.046562	-1.445670	-0.552510

Si	-2.519771	1.962682	0.993641
C	-2.878130	0.303139	0.166380
B	1.634312	-0.802206	2.148397
H	0.608473	-0.879676	2.730589
B	-1.968136	-2.739098	-0.284216
H	-1.394671	-3.759969	-0.085651
B	-3.443970	-2.701324	-1.219694
H	-3.778956	-3.710993	-1.752509
B	-3.709674	-1.091603	-1.975213
H	-4.120896	-1.074912	-3.092158
B	0.220375	-1.134571	-0.198084
B	3.683925	0.847764	1.012419
H	4.105319	1.928466	0.803029
B	4.318438	-0.549755	0.134533
H	5.171859	-0.420956	-0.660672
B	1.935075	0.652767	1.166338
H	1.150347	1.514216	1.045942
B	-3.479539	-2.415241	0.544140
H	-3.805487	-3.233908	1.345449
B	3.204370	-1.493737	2.598580
H	3.335441	-2.072365	3.621856
B	4.473172	-0.468928	1.897288
H	5.529030	-0.305516	2.406343
B	2.287046	-2.211106	1.267864
H	1.689712	-3.227565	1.246266
B	2.982878	0.280187	2.533803
H	2.956645	0.964193	3.496572
B	-3.975827	-0.736620	0.813553
H	-4.529356	-0.445865	1.823090
B	-2.130226	-1.144473	0.782162
H	-1.619855	-1.112310	1.845819
B	4.033420	-1.999747	1.100329
H	4.760014	-2.930272	1.035684
B	-4.275913	0.048105	-0.729519
H	-5.073947	0.910084	-0.872014
B	2.969086	-1.620705	-0.261861
H	2.840030	-2.172384	-1.289191
B	-4.670943	-1.653557	-0.539827
H	-5.809471	-1.991787	-0.569589
B	-2.619098	0.109503	-1.386536
H	-2.105937	0.945501	-2.052028
B	-2.029736	-1.715032	-1.824558
H	-1.505031	-2.007370	-2.848448
C	-1.316305	2.939204	-0.082308
C	-1.762399	1.755272	2.710877
C	-4.109538	2.971712	1.184279

H	-4.548484	3.210723	0.207328
H	-3.911799	3.916502	1.706332
H	-4.863083	2.419860	1.759508
H	-0.781629	1.279513	2.665649
H	-2.401734	1.143014	3.352603
H	-1.639438	2.732051	3.194781
H	-0.469799	2.310527	-0.373751
H	-0.925578	3.815033	0.448786
H	-1.795111	3.283209	-1.002966
C	4.026759	0.674316	-2.971838
C	2.368999	2.851554	-1.580683
C	0.983551	0.354621	-2.754843
H	3.288061	3.258526	-1.152966
H	1.547143	3.087282	-0.906628
H	2.181069	3.374665	-2.523574
H	4.937901	1.097164	-2.538547
H	3.877542	1.135825	-3.953459
H	4.187312	-0.396012	-3.130143
H	0.980962	-0.731789	-2.860971
H	0.940530	0.770910	-3.770528
H	0.061484	0.652636	-2.250762

[B^{TMS}*o*Cb$_2$]$^-$ triplet, gas phase

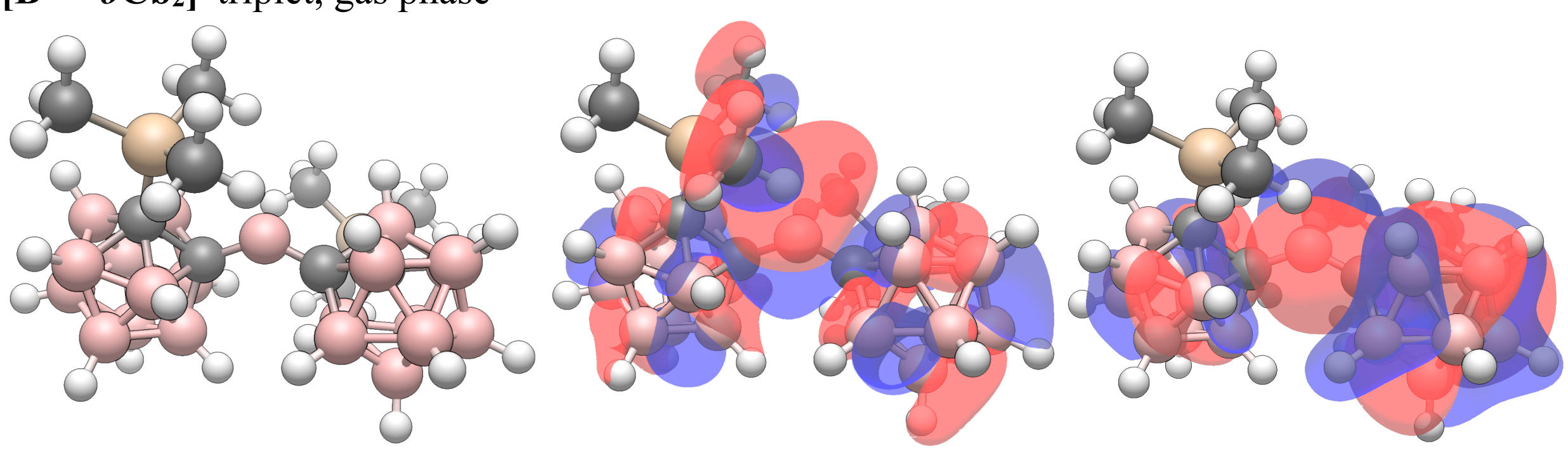

C	-1.358624	-0.374072	0.469194
C	-2.918109	0.009386	-0.074629
Si	-3.132137	1.454333	-1.327932
C	1.092846	0.974354	0.385192
Si	2.995126	-1.673019	-1.203349
C	3.023840	-0.119718	-0.153116
B	-1.253225	-2.056994	0.744927
H	-0.221648	-2.583764	0.531645
B	1.501829	1.495377	1.882645
H	0.622893	1.567718	2.670071
B	2.838302	2.663390	1.720229
H	2.845993	3.645533	2.386085
B	3.374951	2.631620	0.015110
H	3.760829	3.601611	-0.547559

B	-0.218179	0.507525	-0.116888
B	-3.887008	-1.389407	-0.177895
H	-4.691924	-1.439892	-1.040297
B	-4.059069	-0.249519	1.160136
H	-4.986776	0.477800	1.219325
B	-2.177536	-1.426253	-0.631904
H	-1.797212	-1.478389	-1.741571
B	3.126285	1.024858	2.376063
H	3.333026	0.828200	3.526635
B	-2.403567	-2.413434	2.050372
H	-2.199691	-3.293393	2.815436
B	-4.033007	-1.995691	1.478790
H	-5.008088	-2.566887	1.832478
B	-1.437169	-0.931723	2.096915
H	-0.530059	-0.682903	2.800608
B	-2.851691	-2.722727	0.351422
H	-2.970902	-3.808186	-0.104798
B	3.986134	0.077711	1.157381
H	4.710320	-0.812440	1.455673
B	2.169078	-0.102451	1.316141
H	1.710521	-1.108155	1.728928
B	-3.147500	-0.871003	2.545414
H	-3.485230	-0.630910	3.653601
B	4.138310	1.077016	-0.301961
H	4.978764	0.956995	-1.127042
B	-2.453733	0.406418	1.537355
H	-2.261110	1.531552	1.814771
B	4.321077	1.818535	1.302106
H	5.380613	2.201200	1.674105
B	2.388986	1.326418	-0.784660
H	2.090845	1.379327	-1.929033
B	1.658686	2.507059	0.391616
H	0.902908	3.351898	0.048976
C	-2.492616	3.064583	-0.608325
C	-4.985713	1.621203	-1.626780
C	-2.294798	1.046717	-2.957709
H	-5.520247	1.875505	-0.708995
H	-5.415067	0.697942	-2.022037
H	-5.163913	2.418547	-2.355894
H	-3.030995	3.332834	0.303819
H	-2.643282	3.866718	-1.339036
H	-1.427168	3.015236	-0.375891
H	-1.222972	0.895295	-2.817365
H	-2.439740	1.878140	-3.655854
H	-2.717347	0.145645	-3.409012
C	1.431866	-1.744348	-2.248277

C	3.080429	-3.194098	-0.096458
C	4.487479	-1.663673	-2.358150
H	0.536331	-1.807882	-1.629617
H	1.452453	-2.628322	-2.894563
H	1.339247	-0.858516	-2.880700
H	2.205196	-3.253998	0.554083
H	3.969189	-3.165179	0.539270
H	3.119813	-4.106441	-0.700559
H	4.463585	-0.795560	-3.021999
H	4.497753	-2.568130	-2.975748
H	5.421881	-1.622754	-1.792220

$[B^{TMS}$*o*$Cb_2]^-$ triplet, toluene phase (with SMD)

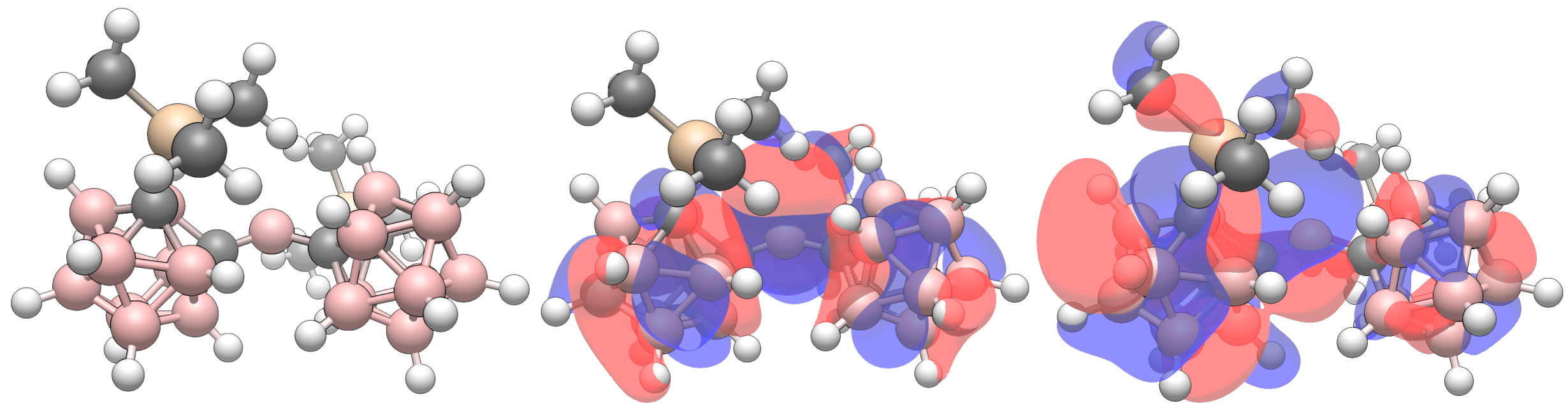

C	1.352389	0.363185	0.458838
C	2.922649	0.006595	-0.069627
Si	3.191561	-1.430583	-1.329902
C	-1.089491	-0.987683	0.361065
Si	-3.020877	1.670807	-1.203057
C	-3.038106	0.107742	-0.159191
B	1.211752	2.040836	0.743908
H	0.174060	2.551031	0.522288
B	-1.485416	-1.516127	1.858687
H	-0.602178	-1.593006	2.640675
B	-2.817443	-2.686936	1.701267
H	-2.818940	-3.673270	2.361371
B	-3.368026	-2.647714	0.001149
H	-3.755883	-3.617258	-0.561358
B	0.222649	-0.521614	-0.140793
B	3.866907	1.423001	-0.150493
H	4.682016	1.494410	-1.000289
B	4.041349	0.277372	1.182019
H	4.980368	-0.432674	1.249812
B	2.163641	1.433293	-0.626969
H	1.793095	1.485833	-1.739588
B	-3.106619	-1.052598	2.365322
H	-3.305687	-0.863011	3.518886
B	2.338895	2.407228	2.065562
H	2.110213	3.278147	2.833748

B	3.980832	2.020422	1.511659
H	4.940429	2.606335	1.882320
B	1.398440	0.909869	2.090363
H	0.487465	0.640593	2.781579
B	2.802096	2.734912	0.374152
H	2.908730	3.825603	-0.071611
B	-3.983277	-0.104216	1.159885
H	-4.710771	0.777073	1.473226
B	-2.163572	0.082020	1.297729
H	-1.708932	1.088424	1.711547
B	3.102043	0.874441	2.559618
H	3.428729	0.633971	3.670795
B	-4.142919	-1.096320	-0.302161
H	-4.993493	-0.981510	-1.116952
B	2.443405	-0.408538	1.534112
H	2.263778	-1.537601	1.802684
B	-4.307157	-1.846280	1.299292
H	-5.359845	-2.238792	1.680925
B	-2.392902	-1.331546	-0.797096
H	-2.104600	-1.381390	-1.943617
B	-1.650117	-2.521358	0.365331
H	-0.895407	-3.363483	0.015475
C	2.559623	-3.046688	-0.621753
C	5.048796	-1.562610	-1.591436
C	2.373654	-1.024195	-2.966854
H	5.575097	-1.824993	-0.670490
H	5.476872	-0.632339	-1.973213
H	5.250789	-2.349500	-2.326788
H	3.101143	-3.326131	0.285845
H	2.714624	-3.841307	-1.360608
H	1.492900	-3.014489	-0.388727
H	1.293336	-0.906435	-2.859382
H	2.557553	-1.844435	-3.670136
H	2.781267	-0.109756	-3.406249
C	-1.454400	1.749451	-2.238997
C	-3.111319	3.179653	-0.084078
C	-4.510620	1.654901	-2.355202
H	-0.558355	1.798762	-1.618892
H	-1.467713	2.648433	-2.865612
H	-1.361845	0.881547	-2.896836
H	-2.240441	3.244209	0.573219
H	-4.005073	3.155966	0.546068
H	-3.147194	4.095479	-0.684381
H	-4.485039	0.793328	-3.028629
H	-4.522980	2.562299	-2.969542
H	-5.449517	1.613958	-1.795441

[B^{Me}*o*Cb$_2$]$^-$ singlet, gas phase

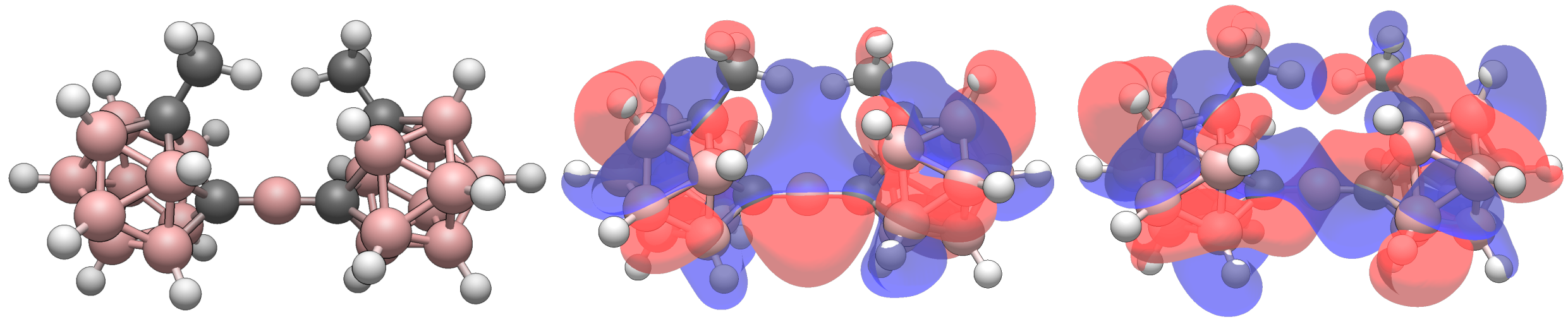

C	1.431318	-0.771108	0.013001
C	2.566275	1.191514	-0.340931
C	1.772522	2.424262	-0.661174
H	1.871003	3.173084	0.127915
H	0.711782	2.169325	-0.753593
H	2.092394	2.861232	-1.609737
C	-1.431339	-0.771098	-0.013024
C	-1.772492	2.424217	0.661226
H	-0.711755	2.169261	0.753618
H	-2.092342	2.861162	1.609808
H	-1.870977	3.173063	-0.127839
C	-2.566267	1.191491	0.340960
B	2.439918	-1.415408	1.122496
H	1.951634	-2.083069	1.966543
B	-2.515579	-1.821161	0.639123
H	-2.048501	-2.781949	1.142842
B	-4.006183	-1.749114	-0.321870
H	-4.586662	-2.752875	-0.566992
B	-3.845944	-0.402362	-1.490341
H	-4.301013	-0.478385	-2.581413
B	-0.000007	-0.743692	-0.000012
B	3.912979	1.123186	0.599983
H	4.307655	2.136853	1.068323
B	3.920027	0.743024	-1.127811
H	4.324716	1.459562	-1.979228
B	2.303478	0.433488	1.117130
H	1.625475	0.963736	1.928022
B	-3.920503	-1.024463	1.308520
H	-4.435408	-1.511013	2.257863
B	4.006168	-1.749118	0.321833
H	4.586642	-2.752887	0.566935
B	4.860412	-0.249159	0.004742
H	6.044989	-0.208669	0.015333
B	2.515569	-1.821140	-0.639169
H	2.048499	-2.781918	-1.142915
B	3.845930	-0.402390	1.490334
H	4.300991	-0.478446	2.581406

B	-3.920020	0.742992	1.127833
H	-4.324700	1.459515	1.979267
B	-2.324299	-0.146392	1.367859
H	-1.721570	0.017826	2.371828
B	3.920495	-1.024426	-1.308540
H	4.435399	-1.510958	-2.257893
B	-3.912971	1.123194	-0.599953
H	-4.307636	2.136876	-1.068270
B	2.324293	-0.146343	-1.367854
H	1.721569	0.017901	-2.371822
B	-4.860416	-0.249156	-0.004742
H	-6.044992	-0.208655	-0.015328
B	-2.303481	0.433496	-1.117128
H	-1.625483	0.963763	-1.928010
B	-2.439936	-1.415393	-1.122530
H	-1.951646	-2.083034	-1.966588

[B^{Me}*o*Cb_2]$^-$ singlet, toluene phase (with SMD)

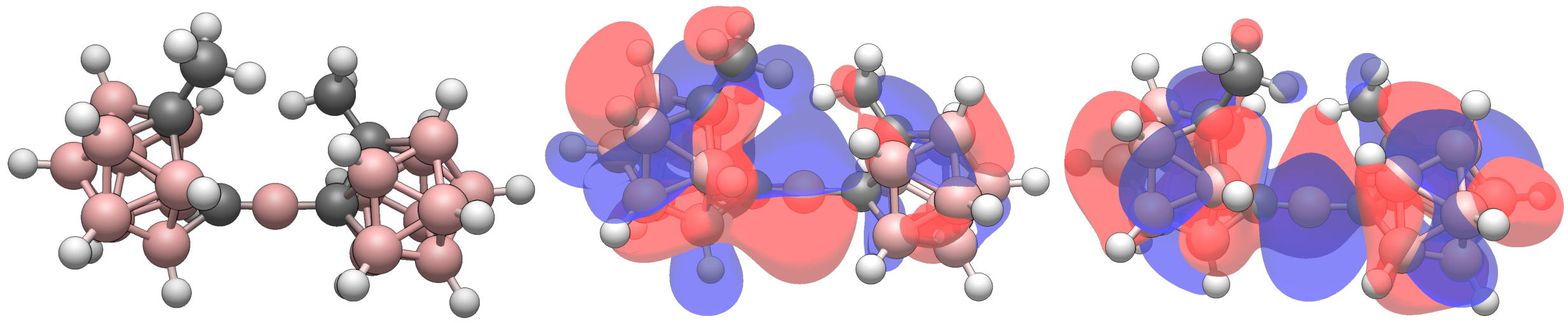

C	1.630326	-0.542842	0.004472
C	2.275781	0.988486	-0.490929
C	1.313029	2.018197	-1.033857
H	1.308431	2.902779	-0.397932
H	0.299800	1.617893	-1.075927
H	1.608664	2.307923	-2.042110
C	-1.222082	-0.919983	-0.117073
C	-1.920017	2.524727	0.782521
H	-0.857029	2.364626	0.986862
H	-2.372476	2.920978	1.695893
H	-1.996546	3.289843	0.004843
C	-2.580100	1.234325	0.343359
B	2.530135	-1.144123	1.315282
H	1.953596	-1.764666	2.133351
B	-2.378520	-1.910523	0.415912
H	-1.992382	-2.968971	0.784943
B	-3.878357	-1.728580	-0.462535
H	-4.455648	-2.714388	-0.789242
B	-3.851747	-0.249817	-1.490549
H	-4.333603	-0.324688	-2.573654
B	0.140247	-0.784856	-0.074450

B	3.598492	1.398692	0.476334
H	3.757356	2.544673	0.704761
B	3.804333	0.762637	-1.161507
H	4.106526	1.472610	-2.053158
B	2.171317	0.604266	1.175424
H	1.361580	1.160401	1.820216
B	-3.729172	-1.148531	1.219109
H	-4.166825	-1.733164	2.156120
B	4.167443	-1.408247	0.679027
H	4.847010	-2.274881	1.108883
B	4.818936	0.155298	0.159996
H	5.971127	0.417312	0.210585
B	2.759863	-1.782264	-0.324713
H	2.325914	-2.834407	-0.626064
B	3.790807	0.061531	1.623623
H	4.200729	0.247077	2.716201
B	-3.827712	0.614606	1.200701
H	-4.208080	1.206333	2.156225
B	-2.132509	-0.198815	1.197559
H	-1.534414	-0.062630	2.214820
B	4.150116	-0.974695	-1.050867
H	4.813052	-1.515254	-1.866184
B	-4.056088	1.180372	-0.445812
H	-4.627092	2.183960	-0.716870
B	2.511083	-0.425288	-1.453402
H	1.925562	-0.498075	-2.470602
B	-4.801653	-0.329617	0.056014
H	-5.984759	-0.405906	0.124909
B	-2.475058	0.736916	-1.155259
H	-1.831650	1.329173	-1.958352
B	-2.339129	-1.208688	-1.285350
H	-1.964607	-1.787384	-2.252168

[B^{Me}*o*Cb$_2$]$^-$ triplet, gas phase

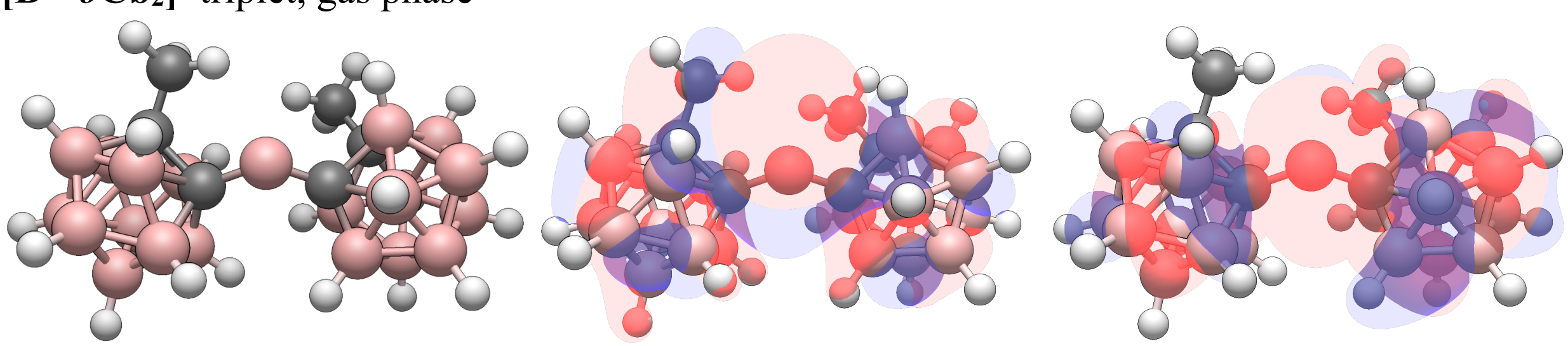

C	-1.456414	0.167713	-0.307345
C	-2.583124	-1.132909	-0.410719
C	-2.114503	-2.396673	-1.094839
H	-1.984251	-3.200353	-0.370187
H	-1.157183	-2.206074	-1.590272

H	-2.843614	-2.705815	-1.843983
C	1.320691	0.354144	-0.648060
C	1.953087	-2.464747	0.654697
H	0.925296	-2.470796	0.273441
H	1.912058	-2.643323	1.731356
H	2.495604	-3.282923	0.175168
C	2.596354	-1.142597	0.351901
B	-1.741141	1.024223	1.148104
H	-0.816773	1.516152	1.680667
B	1.928028	1.726901	0.026878
H	1.164887	2.611905	0.203195
B	3.619417	1.903668	-0.512210
H	4.046995	2.973577	-0.793423
B	4.058196	0.417603	-1.400985
H	4.792096	0.424815	-2.332020
B	-0.076079	-0.024040	-0.993412
B	-3.574152	-1.176380	0.959101
H	-3.906875	-2.241990	1.346801
B	-4.168952	-0.556195	-0.587226
H	-4.901533	-1.204686	-1.249819
B	-1.856334	-0.741131	1.094696
H	-1.053632	-1.485103	1.525249
B	3.245384	1.412128	1.164865
H	3.390938	2.144335	2.085422
B	-3.379032	1.683067	1.014349
H	-3.659947	2.712819	1.526053
B	-4.518268	0.324067	0.906533
H	-5.619083	0.366590	1.341017
B	-2.320371	1.634524	-0.411634
H	-1.788574	2.541526	-0.945031
B	-3.082136	0.199921	1.959118
H	-3.150211	0.150281	3.139271
B	3.607596	-0.308952	1.337033
H	3.900921	-0.811723	2.369224
B	1.893683	0.229978	1.044828
H	1.085449	0.072855	1.889020
B	-4.036642	1.205378	-0.573070
H	-4.781497	1.879730	-1.198443
B	4.100241	-0.926334	-0.255294
H	4.764374	-1.893663	-0.422283
B	-2.807073	0.241378	-1.394562
H	-2.591054	0.133197	-2.546364
B	4.624180	0.688598	0.274936
H	5.758589	0.900301	0.549138
B	2.616068	-0.664012	-1.281116
H	2.327072	-1.466329	-2.101124

B	2.441910	1.106281	-1.584518
H	2.066176	1.522144	-2.625602

[B^{Me}*o*Cb$_2$]$^-$ triplet, toluene phase (with SMD)

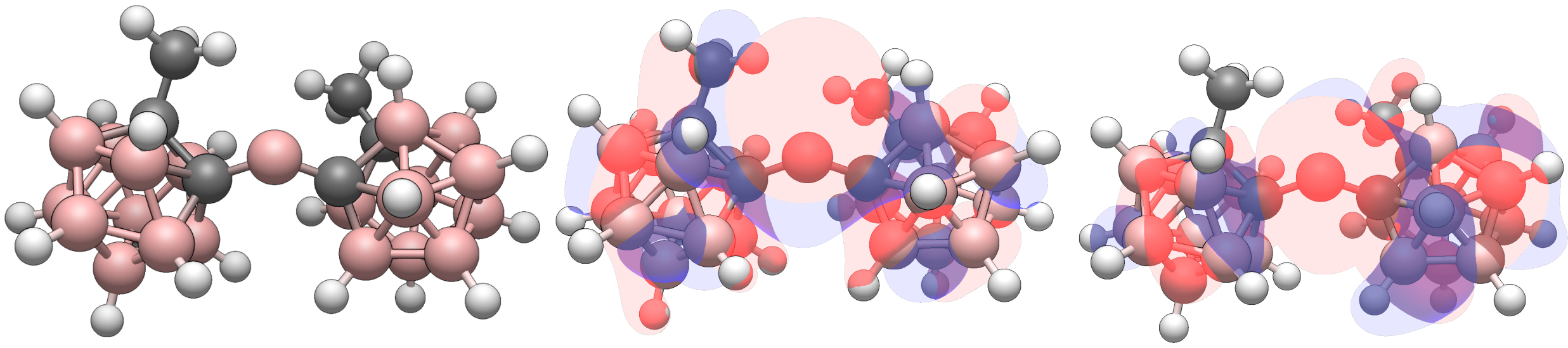

C	-1.458725	0.144826	-0.316849
C	-2.596115	-1.133813	-0.429092
C	-2.155522	-2.404365	-1.119374
H	-1.991171	-3.201353	-0.394240
H	-1.224745	-2.226049	-1.665261
H	-2.919515	-2.723262	-1.828812
C	1.312606	0.337986	-0.646538
C	1.994508	-2.488098	0.675034
H	0.972730	-2.524198	0.279295
H	1.942317	-2.660266	1.752341
H	2.560682	-3.299587	0.211487
C	2.613435	-1.155216	0.369551
B	-1.724480	1.002308	1.141972
H	-0.792270	1.479007	1.674553
B	1.901965	1.713976	0.033835
H	1.130367	2.592252	0.207275
B	3.591866	1.911050	-0.498271
H	4.008228	2.985688	-0.778546
B	4.053062	0.429508	-1.382440
H	4.790817	0.447445	-2.310242
B	-0.077386	-0.051160	-0.999663
B	-3.587457	-1.168865	0.941642
H	-3.938534	-2.229009	1.324663
B	-4.174736	-0.530754	-0.600183
H	-4.919338	-1.162924	-1.262797
B	-1.864493	-0.761021	1.081777
H	-1.070217	-1.516133	1.507495
B	3.216845	1.414355	1.176284
H	3.349125	2.149922	2.096220
B	-3.349779	1.687616	1.012684
H	-3.613152	2.718815	1.530113
B	-4.508528	0.346648	0.897981
H	-5.607495	0.404239	1.334011
B	-2.293414	1.628997	-0.413935
H	-1.744456	2.528841	-0.941827

B	-3.075413	0.195301	1.949373
H	-3.144187	0.141468	3.128918
B	3.605518	-0.300219	1.353543
H	3.906132	-0.790443	2.388725
B	1.882912	0.210313	1.046864
H	1.074302	0.045505	1.888855
B	-4.015210	1.228652	-0.576696
H	-4.749594	1.918377	-1.196754
B	4.113145	-0.911822	-0.235463
H	4.796848	-1.864630	-0.401202
B	-2.803893	0.249480	-1.405877
H	-2.586907	0.146578	-2.557338
B	4.609650	0.710933	0.294208
H	5.739252	0.942238	0.571936
B	2.623377	-0.670774	-1.261909
H	2.349829	-1.475244	-2.084549
B	2.430772	1.098752	-1.575018
H	2.060844	1.511303	-2.619545

References:

(1) Akram, M. O.; Tidwell, J. R.; Dutton, J. L.; Martin, C. D. Bis (1-Methyl-ortho-Carboranyl) Borane. *Angew. Chem. Int. Ed.* **2023**, *62* (34), e202307040.
(2) Costa, G.; Reisenhofer, E.; Stefani, L. Complexes of copper (I) with triphenylphosphine. *J. Inorg. Nucl. Chem.* **1965**, *27* (12), 2581-2584.
(3) Li, Y.; Tamizmani, M.; Akram, M. O.; Martin, C. D. Carborane–arene fused boracyclic analogues of polycyclic aromatic hydrocarbons accessed by intramolecular borylation. *Chem. Sci.* **2024**, *15* (20), 7568-7575.
(4) Tsuji, M. On attempts at generation of carboranyl carbocation. *J. Org. Chem.* **2003**, *68* (25), 9589-9597.
(5) *Gaussian 16 Rev. C.01*; Wallingford, CT, 2016.
(6) Chai, J.-D.; Head-Gordon, M. Long-range corrected hybrid density functionals with damped atom–atom dispersion corrections. *Phys. Chem. Chem. Phys.* **2008**, *10* (44), 6615-6620.
(7) Weigend, F. Accurate Coulomb-fitting basis sets for H to Rn. *Phys. Chem. Chem. Phys.* **2006**, *8* (9), 1057-1065.
(8) Weigend, F.; Ahlrichs, R. Balanced basis sets of split valence, triple zeta valence and quadruple zeta valence quality for H to Rn: Design and assessment of accuracy. *Phys. Chem. Chem. Phys.* **2005**, *7* (18), 3297-3305.
(9) Marenich, A. V.; Cramer, C. J.; Truhlar, D. G. Universal Solvation Model Based on Solute Electron Density and on a Continuum Model of the Solvent Defined by the Bulk Dielectric Constant and Atomic Surface Tensions. *J. Phys. Chem. B* **2009**, *113* (18), 6378-6396.
(10) Glendening, E. D.; Landis, C. R.; Weinhold, F. Natural bond orbital methods. *WIREs Computational Molecular Science* **2012**, *2* (1), 1-42.
(11) Bayly, C. I.; Cieplak, P.; Cornell, W.; Kollman, P. A. A well-behaved electrostatic potential based method using charge restraints for deriving atomic charges: the RESP model. *J. Phys. Chem.* **1993**, *97* (40), 10269-10280.
(12) Humphrey, W.; Dalke, A.; Schulten, K. VMD: Visual molecular dynamics. *Journal of Molecular Graphics* **1996**, *14* (1), 33-38.
(13) Lu, T. A comprehensive electron wavefunction analysis toolbox for chemists, Multiwfn. *J. Chem. Phys.* **2024**, *161* (8).
